# Metabolomics and Lipidomics Explore Phenotype-Specific Molecular Signatures for Phenylketonuria

**DOI:** 10.3390/ijms26157171

**Published:** 2025-07-25

**Authors:** Buket Yurteri Şahiner, Ali Dursun, Basri Gülbakan

**Affiliations:** 1 Department of Pediatric Basic Sciences, Pediatric Metabolism Division, Institute of Child Health, Hacettepe University, Sıhhıye, Ankara 06100, Türkiye; yurterib@gmail.com; 2 Department of Pediatrics, Division of Pediatric Metabolism, Faculty of Medicine, Hacettepe University, Sıhhıye, Ankara 06100, Türkiye; adursun@hacettepe.edu.tr

**Keywords:** phenylketonuria, untargeted metabolomics, untargeted lipidomics, biomarker discovery

## Abstract

Phenylketonuria (PKU) is a monogenic disorder caused by pathogenic variants in the gene encoding phenylalanine hydroxylase (PAH), an enzyme essential for phenylalanine (Phe) metabolism. It is characterized by elevated Phe levels, leading to a wide spectrum of clinical phenotypes. These phenotypes are characterized by varying Phe accumulation, dietary tolerance, and heterogeneous cognitive and neurological outcomes, but current monitoring methods, focused primarily on blood Phe levels, are limited in capturing this variability. In this study, we applied mass spectrometry-based advanced quantitative amino acid analyses, untargeted metabolomics, and lipidomics analyses. We examined the plasma metabolite and lipid profiles in a total of 73 individuals with various PKU phenotypes against healthy controls to see how the metabolome and lipidome of the patients change in different phenotypes. We investigated whether novel markers could be associated with metabolic control status. By elucidating the metabolic and lipid fingerprints of PKU’s phenotypic variability, our findings may provide novel insights that could inform the refinement of dietary and pharmacological interventions, thereby supporting the development of more personalized treatment strategies.

## 1. Introduction

Phenylalanine (Phe), an essential amino acid for protein synthesis and growth, is metabolized by the enzyme phenylalanine hydroxylase (PAH). This enzyme, using cofactors BH4, oxygen, and Fe^2+^, converts L-Phe to L-Tyrosine. Deficient PAH activity leads to Phe accumulation and results in hyperphenylalaninemia (HPA). Elevated Phe levels result from over 200 pathogenic variants in the PAH gene, defects in BH4 metabolism, or mutations in DNAJC12, which supports phenylalanine hydroxylase folding [1,2]. Based on the blood Phe levels at diagnosis, clinical phenotypes are classified as classical PKU, moderate PKU, mild PKU, and mild HPA [3,4]. PKU is the most severe form and is characterized by minimal PAH activity with average blood Phe > 1200 µmol/L, and a dietary tolerance < 20 mg/kg/day Phe intake. This group of patients is at risk of severe and irreversible intellectual disability due to Phe neurotoxicity without strict dietary control to maintain their Phe levels between 120 and 360 µmol/L [5]. In moderate PKU, Phe ranges between 900–1200 µmol/L and still requires dietary restriction (20–50 mg/kg/day) of Phe intake. Mild PKU and mild HFA (blood Phe 600–900 µmol/L) can be managed with more flexible diets. Another distinct phenotype is BH4-responsive HPA and is identified by a ≥30% reduction in blood Phe level following a BH4 loading test [6]. Advancements in newborn screening and analytical methods allow early detection of all PKU forms, initiating timely dietary and pharmacological interventions that enable near-normal development and lives [7]. However, despite these advances, effective PKU monitoring remains a persistent challenge, demanding periodic Phe assessment, particularly during the critical neurodevelopmental period [8,9,10]. At elevated levels, Phe competes with large neutral amino acids for transport across the blood–brain barrier and into other tissues, leading to amino acid imbalances [11,12], which in turn contribute to myelin damage and oligodendrocyte dysfunction [13,14]. Inhibited activity of certain enzymes such as pyruvate kinase, tyrosine hydroxylase, tryptophan hydroxylase, and HMG-CoA reductase as a result of Phe accumulation has also been linked to neurological dysfunction [15]. These metabolic disruptions cumulatively lead to oxidative stress, disruption of lipid and protein metabolism, calcium homeostasis, and neurotransmitter synthesis in the brain [16].

The current diagnostic toolkit for PKU relies on measurement of blood phenylalanine (Phe), tyrosine (Tyr), and Phe/Tyr ratios and downstream metabolites associated with dopamine and serotonin metabolism [17]. In PKU management, these tests need to be frequently repeated to monitor metabolic control and evaluate clinical outcomes. However, while still being the gold standard, phenotypic differences, different cognitive outcomes, and metabolic control status at identical treatment regimens cannot always be fully elucidated by Phe measurements [18].

Omics approaches, such as metabolomics and lipidomics, have emerged as highly valuable tools for understanding various diseases, including inborn errors of metabolism (IEM). First, as metabolites are the end products of downstream metabolic pathways, omics-wide analyses can be useful for the discovery of new biomarkers, alternative to canonical biomarkers, and can expand the diagnostic toolkit. Second, these analyses offer a holistic perspective and can better reflect the clinical phenotypes and treatment response. Third, they are very useful to elucidate the associated biochemical pathways affected as a result of the metabolic defect. Owing to all these advantages, metabolomics and lipidomics are also becoming part of the diagnostic and prognostic work-up in IEM.

Metabolomics and lipidomics can offer a more comprehensive view, specifically for PKU, by expanding the existing biomarkers to a broader metabolic profile and delineating affected pathways not discernible by isolated Phe measurements alone. As Phe is usually measured at discrete intervals, it does not show the long-term adherence to dietary intervention alone. This limitation can be addressed by either using a fingerprick test for daily at-home Phe assessment, analogous to seven-point blood glucose profiling [19,20], or by clinical biomarkers similar to how HbA1c is used to monitor glucose fluctuations in diabetes. Stemming from this idea, several studies have been conducted using omics analyses. In untargeted metabolomics analyses measured in blood, arginine, tyrosine, 2-aminobutyric acid, propionylcarnitine, carnitine, 2-aminoadipic acid, amino hippuric acid, urocanic acid, trimethylamine N-oxide, butyrylcarnitine, tyrosine, glutamine, and creatinine were found to be differential in addition to the canonical PKU markers of phenylalanine, phenylpyruvic acid [21]. PKU patients were suggested to have a very specific sub-metabolome, highlighting unique metabolic signatures [22]. Urinary metabolomics studies also showed altered phenylacetic acid, phenyllactic acid, n-phenylacetylglutamine, n-acetylphenylalanine, phenylpyruvic acid, urocanic acid, o-hydroxyphenyl acetic acid, butyrylcarnitine, and imidazole lactic acid [23,24].

Most notably, Amadori rearrangement products (ARPs), namely Phe-hexose, Met-hexose, and citrulline-hexose, have recently been proposed as potential biomarkers for inborn errors of metabolism, including PKU, confirmed by infrared ion spectroscopy, nuclear magnetic resonance (NMR) spectroscopy, and mass spectrometry (MS) [25]. Adult PKU patients were found to have unique phenylalanine adducts in their plasma, specifically n-lactoyl-phenylalanine [26], which was later proven to perform better than Phe in clinical scores by targeted metabolomics [27]. Most recently, metabolomics and lipidomics were used to assess the molecular changes in early treated PKU patients, pointing to new metabolic perturbations including glyoxylate and dicarboxylate metabolism [28].

In addition, lipidomic analyses demonstrated abnormal phospholipid profiles in different studies in PKU patients. These are abnormal levels of omega-6 to omega-3 fatty acids, docosahexaenoic acid (DHA), arachidonic acid (AA), eicosapentaenoic acid (EPA) [29], free carnitine, and lower levels of fatty acids, γ-linolenic acid, and dihomo γ-linolenic acid [30]. Variation in phosphatidylcholines a polyunsaturated fatty acids (PUFA) has also been observed, underscoring the importance of further lipidomics studies in PKU [31,32].

Despite promising insights from prior metabolomics and lipidomics studies, a comprehensive evaluation comparing these profiles across different PKU phenotypes and metabolic control statuses is still lacking. We hypothesized that (i) distinct PKU phenotypes would exhibit unique metabolomic and lipidomic signatures; (ii) specific metabolites or lipid species would correlate with dietary adherence and metabolic control, potentially serving as additional novel biomarkers for clinical monitoring; (iii) metabolomics/lipidomics could explore affected metabolic pathways beyond the canonical Phe pathways. To test these experimentally and to address this gap, untargeted metabolomics and lipidomics alongside quantitative amino acid profiling were employed. This study expands on the previous findings, aiming to reveal the molecular differences among individuals with varying clinical subtypes of PKU by comparing their metabolome and lipidome profiles with healthy controls. The effect of metabolic control on omics profiles is studied, and a new putative biomarker candidate for metabolic control is discussed. These results are expected to provide new insights into disease management and treatment.

## 2. Results

### 2.1. Clinical Evaluation and Patient Stratification

**Figure 1:**
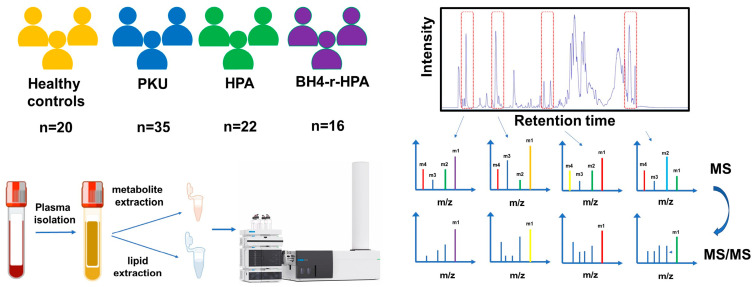
Schematic representation of the metabolomics/lipidomics analyses.

According to Guldberg [4] classification, of 73 phenylketonuria patients, 29 had classical PKU, 3 had moderate PKU, 3 had mild PKU, and 22 had mild hyperphenylalaninemia (HPA); 16 with BH4-responsive HPA (BH4-r-HPA) were included in this study. The control group involved 20 individuals without any metabolic disorders (Tables S1 and S2). Figure 1 summarizes the overall experimental workflow used in this study. It includes the patient cohort and healthy individuals, plasma sample isolation and sample preparation, untargeted metabolomics, and lipidomics data acquisition using LC-Q-TOF-MS.

PKU patients with Phe ≥ levels 10 mg/dL ≈ 605 µM (19 males, 16 females) had a median age of 10.29 years (IQR 4.45–16.12). Mild HPA patients with blood Phe levels ≤ 10 mg/dL ≈ 605 µM (10 males, 12 females) had a median age of 7.64 years (IQR 3.55–11.72). BH4-r-HPA patients (8 males, 8 females) had a median age of 8.5 years (IQR 4.63–12.37). Median BMI was 20.40 (IQR 15.42–25.37) for PKU, 17.48 (IQR 13.97–20.99) for HPA, and 20.09 (IQR 14.94–25.24) for BH4-r-HPA. Median body weight and height were 41.98 kg/138.44 cm (PKU), 27.31 kg/120.95 cm (HPA), and 35.52 kg/127.91 cm (BH4-r-HPA) (Table 1).

Venous blood was collected in the morning during fasting. The mean and median Phe levels were used to determine diet compliance and metabolic control status of the patients. All 35 PKU patients were under diet restriction, 26 had good control (median blood Phe level < 4 mg/dL ≈ 242 µM), and 9 had poor diet control (median blood Phe > 6 mg/dL ≈ 360 µM). A total of 22 patients with mild HPA were under a free diet, 18 patients had good control, and 4 patients had poor control. A total of 16 BH4-r-HPA patients were under a free diet, and only one BH4-r-HPA patient had poor control.

### 2.2. Quantitative Targeted Amino Acid Analysis Results

Plasma quantitative plasma amino acid profiling and omics analyses were evaluated in all patients. Plasma amino acid concentration levels shown in Figure S1 and Table S4 show variations in all measured amino acid concentrations. Specifically, L-threonine, taurine, L-ornithine, L-tryptophan, L-cystine, L-glycine, L-proline, L-asparagine, and phenylalanine were identified as the most statistically significant amino acids, determined by the Kruskal–Wallis test with *p* < 0.05.

### 2.3. Untargeted Metabolomics Results

**Figure 2:**
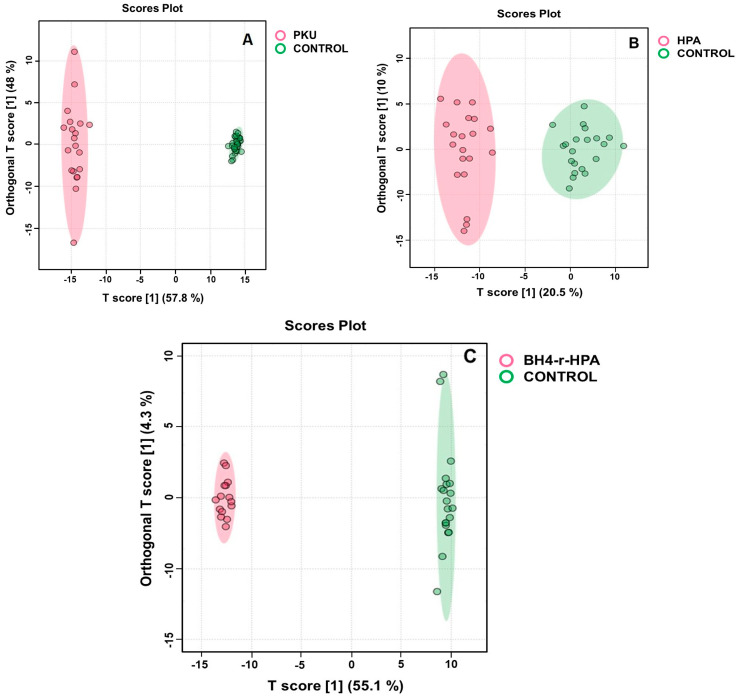
Orthogonal partial least squares discriminant analysis (OPLS-DA) of (**A**) PKU, (**B**) HPA, and (**C**) BH4-r-HPA subgroups against healthy controls.

**Figure 3:**
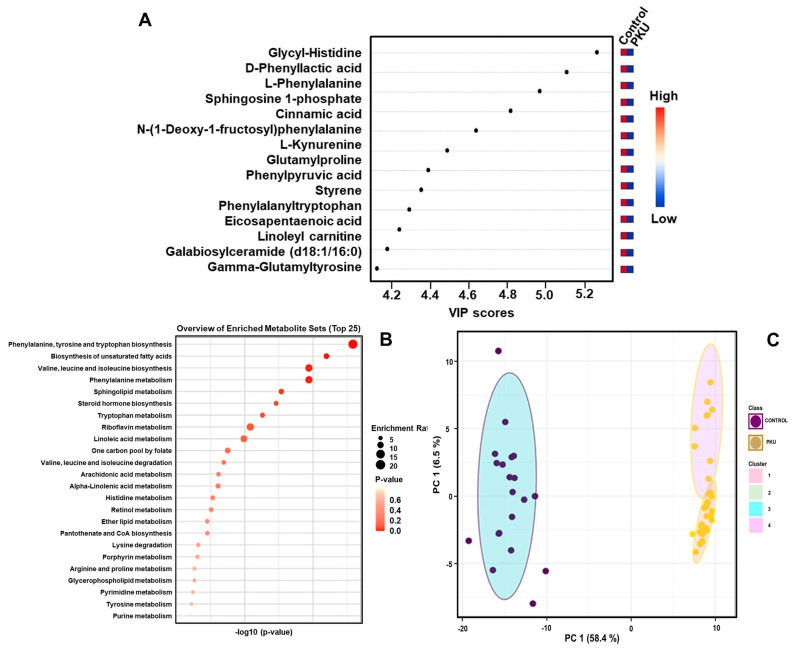
(**A**) Variable importance of projection (VIP) score plots, (**B**) pathway mapping, and (**C**) K-means clustering plots of PKU patients.

In the untargeted metabolomics analyses of patients (which consist of 29 classical PKU, 3 moderate PKU, and 3 mild PKU) and control groups, a total of 1490 features were detected between PKU patients and compared to healthy control samples after raw spectra processing and feature filtering based on abundance and variance. Principal component analysis (PCA) showed a clear separation, indicating significant perturbations in PKU patients compared to healthy individuals (Figure S2). OPLS-DA analysis showed that PKU patients displayed a clearly distinguishable metabolic profile compared to the control group, with R2Y and Q2 values of 0.888 and 0.843, respectively (Figure 2A and Figure S2A), showing the model was appropriate. A total of 63 metabolites were statistically found (*p* < 0.05, fold change > 2.0) between PKU patients and the control group by the Benjamini and Hochberg algorithm (Figure S3). A total of 15 metabolites with the highest VIP scores (Figure 3A) were selected to show the most significant metabolites.

In metabolomics analyses of mild HPA and BH4-r-HPA groups, a total of 1043 features were detected for the mild HPA cohort and 985 features for the BH4-r-HPA following raw spectra processing and feature filtering based on abundance and variance.

PCA analysis showed that patients were distinctively different from healthy controls based on their metabolic profiles, as shown in Figure S2B,C. To further model the data, we again used OPLS-DA analysis, and it yielded strong separation, with R2Y and Q2 values of 0.919 and 0.881 for mild HPA, and 0.935 and 0.876 for BH4-r-HPA patients, respectively (Figure 2B for HPA and Figure 2C for BH4-r-HPA).

**Figure 4:**
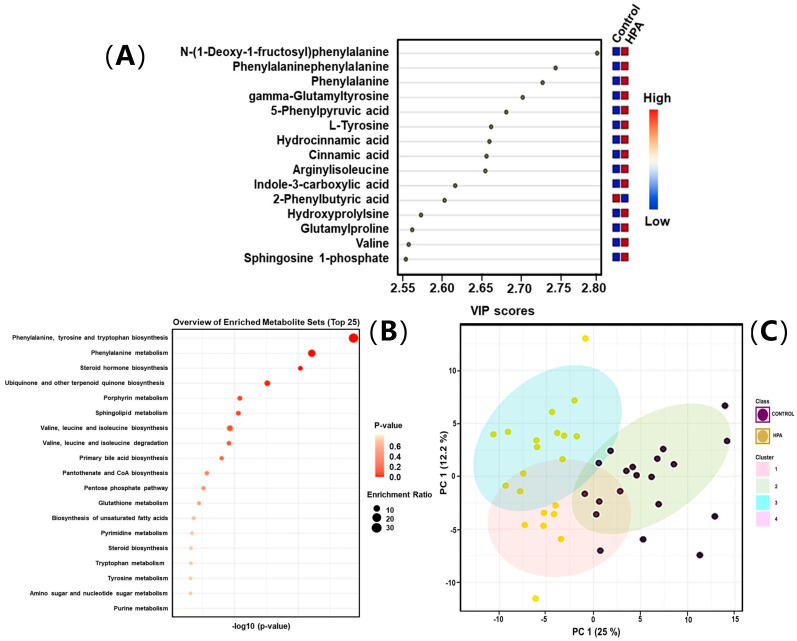
(**A**) Variable importance of projection (VIP) score plots, (**B**) pathway mapping, and (**C**) K-means clustering plots of Mild HPA patients.

**Figure 5:**
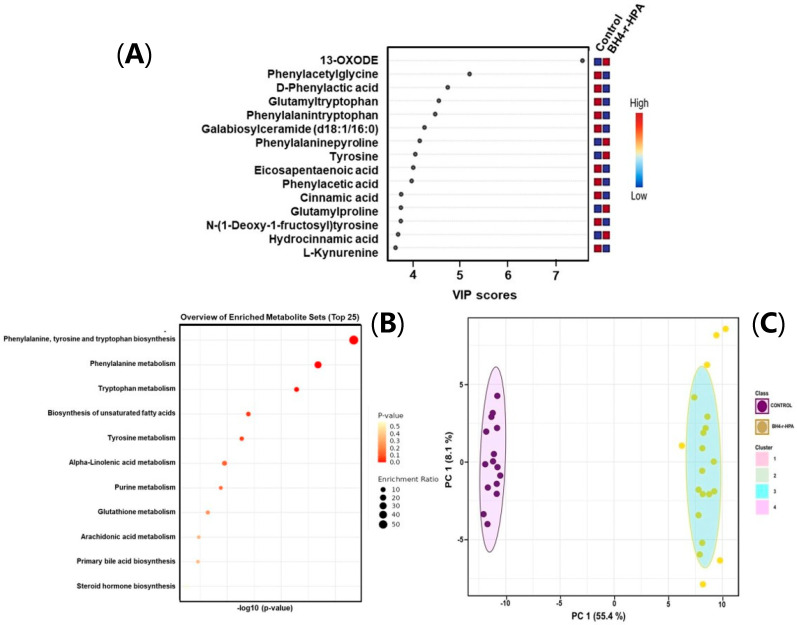
(**A**) Variable importance of projection (VIP) score plots, (**B**) pathway mapping, and (**C**) K-means clustering plots of BH4-r-HPA patients.

For the mild HPA, a total of 54 identified metabolites were statistically significant (*p*-value < 0.05, fold change > 2.0), 30 being upregulated and 24 downregulated (Figure S4). In the BH4-r-HPA group, 43 identified metabolites were statistically significant (*p*-value < 0.05, fold change > 2.0), comprising 28 upregulated and 15 downregulated metabolites (Figure S5). The most significant differences among these groups were depicted through VIP scores (Figure 4A for mild HPA, Figure 5A for BH4-r-HPA).

In the PKU group, glycyl-histidine, phenylalanyltyroptophan, d-phenyl lactic acid, d-phenylalanine, phenyl pyruvic acid, n-(1-deoxy-1-fructosyl) phenylalanine, cinnamic acid, sphingosine 1-phosphate, L-kynurenine, glutamyl proline, and styrene represented the top discriminating metabolites relative to healthy controls. In the mild HPA phenotype, cinnamic acid, N-(1-deoxy-1-fructosyl) phenylalanine, L-phenylalanine, arginylisoleucine, hydrocinnamic acid, sphingosine 1-phosphate, indole-3-carboxylic acid, valine, hydroxyprolylysine, L-tyrosine, phenylalanine-phenylalanine, gamma-glutamyltyrosine, 5-phenylpyruvic acid, glutamylproline, and 2-phenylbutyric acid metabolites showed differential alterations relative to healthy controls. In BH4-r-HPA patients, 13-OXODE, phenylacetylglycine, D-phenyllactic acid, glutamyltryptophan, phenylalanine-tryptophan, galabiosylceramide (d18:1/16:0), phenylalanine-proline, tyrosine, eicosopentaenoic acid, phenylacetic acid, cinnamic acid, glutamylproline, N-(1-deoxy-1-fructosyl) tyrosine, hydrocinnamic acid, and L-kynurenine metabolites were found to be differential relative to the healthy control group.

Metabolite enrichment analysis was applied to all three different clinical phenotypes for the differential metabolites, which are depicted in Figure 3B, Figure 4B and Figure 5B. For the PKU phenotype, phenylalanine, tyrosine, and tryptophan biosynthesis pathway, biosynthesis of unsaturated fatty acid pathway, valine, leucine and isoleucine pathways, and sphingolipid pathways were enriched. For mild HPA phenotype, similarly, the phenylalanine, tyrosine, and tryptophan biosynthesis pathway, phenylalanine pathway, steroid hormone pathway, ubiquinone pathway, and sphingolipid pathways were enriched. Finally, for the BH4-r-HPA, the phenylalanine, tyrosine, and tryptophan biosynthesis pathway, phenylalanine biosynthesis of unsaturated fatty acid pathway, and tryptophan pathway were the enriched pathways. It was notable that in all three clinical phenotypes, phenylalanine, tyrosine, phenylalanine pathway, sphingolipid metabolism pathway, tryptophan metabolism pathway, steroid hormone biosynthesis, and fatty acid were shared across different phenotypes. Of these pathways, enrichment of phenylalanine, tryptophan pathways overlap with the amino acids that were found to be significant in quantitative analysis.

We noticed that PKU patients exhibited two different subclusters within the PCA plots. To capture this more clearly, we performed K-means clustering analyses in all patient subgroups (PKU, mild HPA, and BH4-r-HPA patients). This revealed two distinct subgroups for PKU (Figure 3C) and also in HPA patients (Figure 4C) indeed existed, though not for BH4-r-HPA patients (Figure 5C). To determine the cause of the presence of two subclasses in PKU and mild HPA groups, we found that the patients contributing to this discrimination were patients under poor and good metabolic control. Afterwards, these subclusters were compared to the control groups.

In good metabolic control PKU patients, phenylvaleric acid, p-hydroxyphenylacetic acid, methyltetrehydrofolate, phenylalaninetyrosine, indole acetic acid, L-tyrosine, L valine, and glutamyltryrptophan decreased, and cinnamic acid, L-phenylalanine, stearoylcarnitine, N-docosohexanoyl phenylalanine, sphingosine phosphate, L-palmitoylcarnitine, and galabiosylceramide increased relative to the control group.

**Figure 6:**
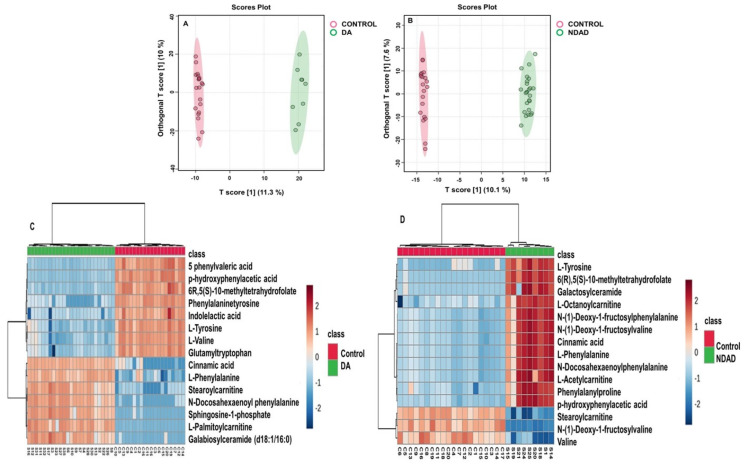
Orthogonal partial least squares discriminant analysis (OPLS-DA) modeling and heatmaps of PKU patients against healthy controls. (**A**,**C**): Good metabolic control. (**B**,**D**): Poor metabolic control.

In poor metabolic control PKU patients, phenylalanine, p-hydroxyphenyl acetic acid, n-(1-deoxy-1-fructosyl) phenylalanine, n-(1-deoxy-1-fructosyl) valine, and n-docosohexanoyl phenylalanine increased, while n-(1-deoxy-1-fructosyl) tyrosine, stearoylcarnitine, and valine decreased compared to the control group. These results are demonstrated in Figure 6A–D. We also performed MS/MS analysis for n-(1-deoxy-1-fructosyl) phenylalanine to validate this metabolite (Figure S8).

**Figure 7:**
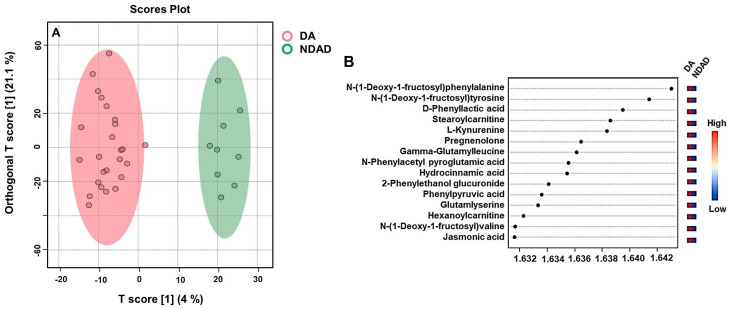
(**A**) Orthogonal partial least squares discriminant analysis (OPLS-DA) modeling of PKU patients with good metabolic control versus poor metabolic control (**A**) and VIP score plot of the group comparison (**B**).

When poor (which is also denoted as non-dietary adherent-NDAD) and good metabolic control groups (also denoted as dietary adherent-DA) of PKU patients were compared, we found that n-(1-deoxy-1-fructosyl) phenylalanine, gamma-glutamylleucine, d-phenylactic acid, phenylpyruvic acid, glutamylserine, n-(1-deoxy-1-fructosyl) valine, and n-phenylacetyl pyroglutamic acid were increasing, while n-(1-deoxy-1-fructosyl) tyrosine, stearoylcarnitine, L-kynurenine, pregnenolone, hydrocinnamic acid, 2-phenylethanol glucuronide, hexanoylcarnitine, and jasmonic acid were decreasing (Figure 7A,B).

**Figure 8:**
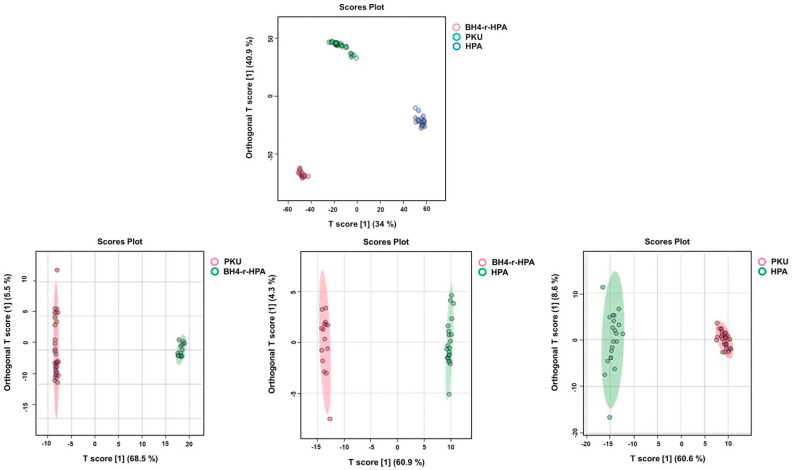
Partial least squares discriminant analysis (PLS-DA) of a 3-group comparison and comparison of each clinical phenotype with respect to each other.

Finally, the comparison of three clinical phenotypes (PKU, mild HPA, and BH4-r-HPA) with healthy individuals by PCA and OPLS-DA analyses clearly demonstrated that the clinical phenotypes were distinguishable based on their metabolome profiles (Figure 8, Figures S6 and S7).

**Figure 9:**
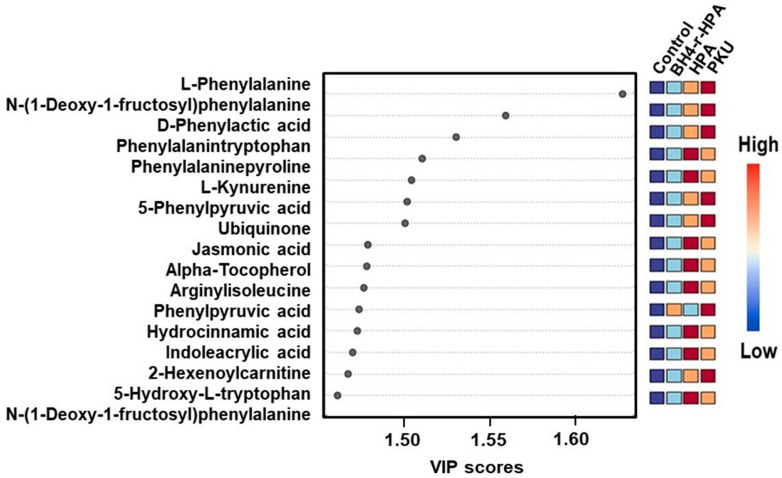
Variable importance of projection (VIP) score plot of a 4-group comparison of the metabolomics data.

The most significant differences among these groups were identified by VIP scores from the PLS-DA analysis. Metabolites shared across different groups included L-phenylalanine, n-(1-deoxy-1-fructosyl) phenylalanine, d-phenyllactic acid, phenylalanyltryptophan, phenylalanine-proline, L-kynurenine, 5-phenylvaleric acid, ubiquinone, jasmonic acid, alpha-tocopherol, phenylpyruvic acid, hydrocinnamic acid, indoleacrylic acid, 2-hexenoylcarnitine, 5-hydroxy-L-tryptophan, and n-(1-deoxy-1-fructosyl) valine (Figure 9, Figures S9 and S10), This shows that, irrespective of the clinical phenotype, metabolites related to Phe metabolism are common in PKU.

### 2.4. Untargeted Lipidomics Analysis Results

**Figure 10:**
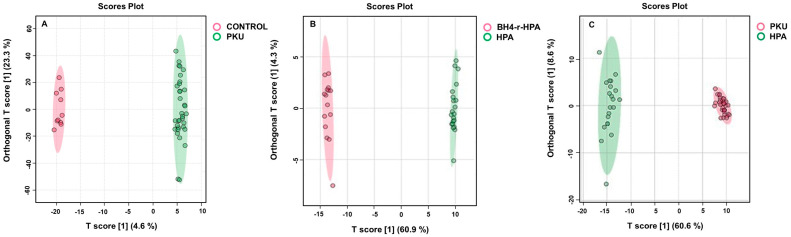
Orthogonal partial least squares discriminant analysis (OPLS-DA) of the lipidomics data (**A**) PKU, (**B**) HPA, and (**C**) BH4-r-HPA subgroups against healthy controls.

In lipidomic analysis of the PKU, mild HPA and BH4-r-HPA phenotypes, differences among groups and control groups were depicted by OPLS-DA, similar to metabolomics data (Figure 10A–C).

**Figure 11:**
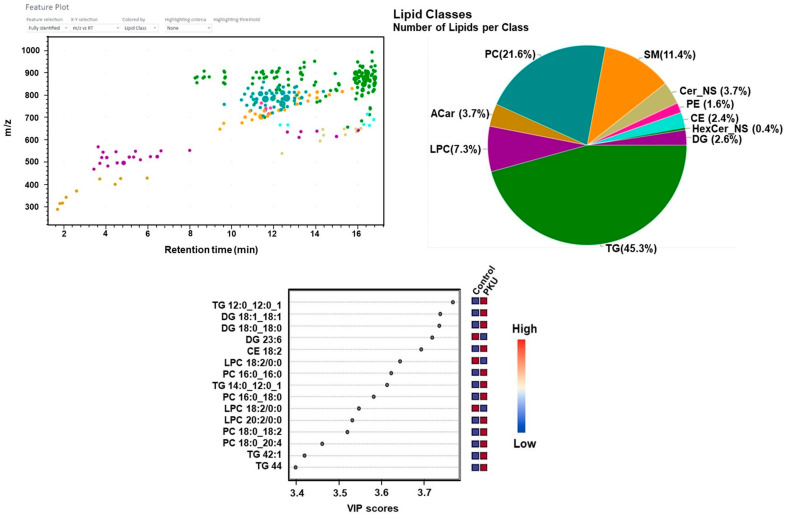
Feature plots, variable importance of projection (VIP) score plots for PKU lipidomic analyses.

Triglycerides were the most prevalent lipid class in PKU, mild HPA, and BH4-r-HPA groups. When the lipidomics data of the PKU group were analyzed, we found that 45.7% of the total identified lipids, followed by phosphatidylcholine (21.2%), sphingomyelin (11.4%), and lysophosphatidylcholine (7.3%) (Figure 11).

**Figure 12:**
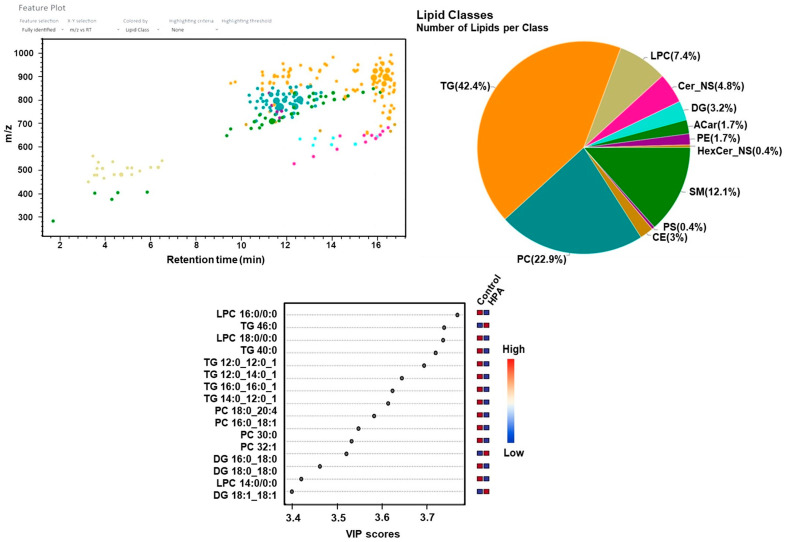
Feature plots, variable importance of projection (VIP) score plots for mild HPA lipidomic analyses.

In HPA patients, the most prevalent lipid classes were again triglycerides (42.4%), followed by phosphatidylcholines (22.3%), sphingomyelins (13.4%), and lysophosphatidylcholines (7.6%) (Figure 12).

**Figure 13:**
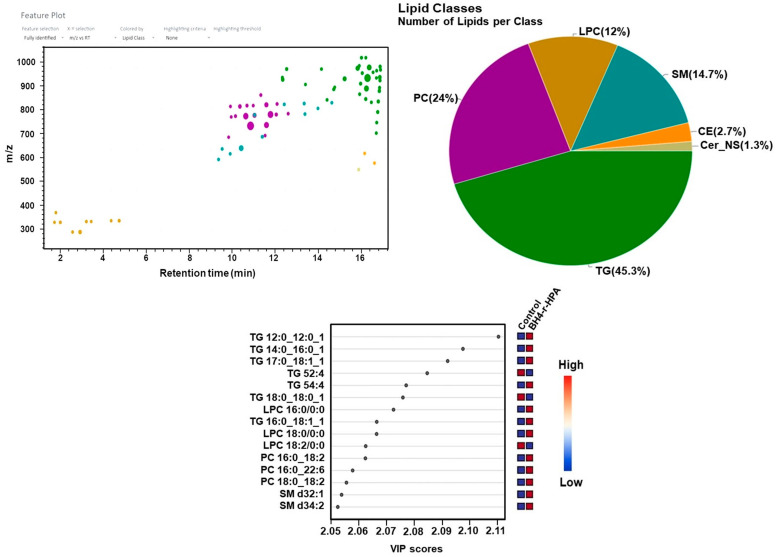
Feature plots, variable importance of projection (VIP) score plots for BH4-r-HPA lipidomic analyses.

In BH4-responsive HPA patients, the most prevalent lipid classes were triglycerides (45.3%), followed by phosphatidylcholines (24%), sphingomyelins (14.7%), and lysophosphatidylcholines (12%) (Figure 13).

For PKU, the differentially expressed lipids included increased TG 42:1, TG 44:00, DG 18:1/18:1, DG 18:0/18:0, PC (18:0/20:4), PC (16:0/18:0), PC (16:0/16:0), and LPC (20:2), while LPC (18:2) decreased. In the HPA patient group, increased TG 46:00, DG 16:0/18:0, and DG 18:0/18:0 were observed. BH4-r-HPA patients showed increased PC (16:0/18:2), PC (16:0/22:6), PC (18:0/18:2), SM d32:1, SM d34:2, LPC (18:0), LPC (18:2), TG 52:4, and TG 54:4.

**Figure 14:**
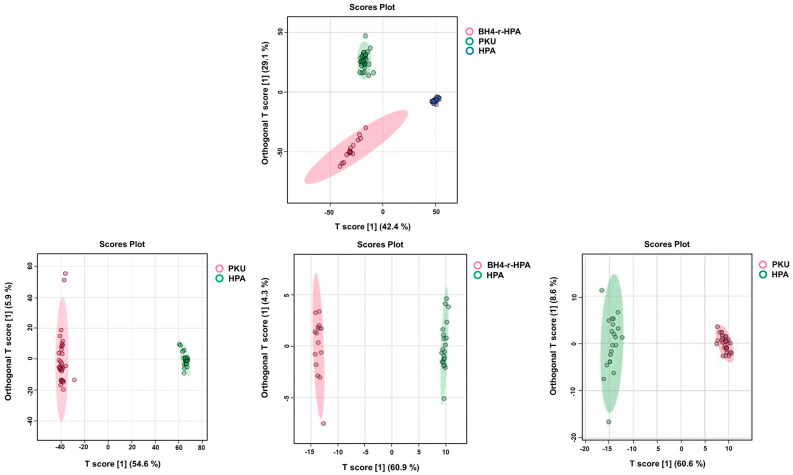
Partial least squares discriminant analysis (PLS-DA) of a 3-group comparison for lipidomics data and comparison of each clinical phenotype with each other.

Next, we compared PKU, mild HPA, and BH4-r-HPA patients and each clinical phenotype with each other to reveal if shared lipidomic profiles exist across phenotypes. It was notable that, similar to the metabolomic profiles, the lipidomic data also showed significant differences among all three clinical phenotypes (Figure 14).

When dietary adherent PKU patients (with good metabolic control) were compared to healthy controls, the R2Y and Q2 values of the OPLS-DA model were 0.572 and 0.284, respectively. For dietary non-adherent PKU patients (with poor metabolic control) versus healthy controls, the R2Y and Q2 values were 0.566 and 0.334, respectively. Based on these poor Q2 scores, we concluded that there was no significant discrimination based on their lipid profiles to monitor metabolic control status.

## 3. Discussion

PKU is a neurometabolic disease effectively treated if the dietary intervention is initiated timely in the infancy period. This effectively eliminates the direct neurotoxic effects of high Phe levels and mitigates its clinical complications. Keeping Phe in the optimal target ranges is crucial, and frequent assessment of blood Phe is thus critical. However, while phenylalanine itself is a useful prognostic marker, new metabolites and lipid markers that could complement Phe and Phe/Tyr measurements could help understand the clinical outcomes. In this context, targeted amino acid profiling, untargeted metabolomics, and untargeted lipidomics experiments were performed in classical phenylketonuria (PKU), mild hyperphenylalaninemia (HPA), and BH4-responsive HPA (BH4-r-HPA) clinical subgroups in this study.

In targeted metabolomics analyses, quantitative amino acid profiles were performed in the plasma samples of the patients in all 73 patients and 20 controls. We found that L-threonine, taurine, L-ornithine, L-tryptophan, L-cystine, L-glycine, L-proline, L-asparagine, and phenylalanine were differentially altered. This revealed that all measured amino acids were differentially altered. L-threonine, taurine, L-ornithine, L-tryptophan, L-cystine, L-glycine, L-proline, L-asparagine, and phenylalanine were found to be the most statistically significant, as calculated by the Kruskal–Wallis test with a *p*-value of <0.05. Phe and Phe/Tyr ratios were higher in PKU patients compared to HPA and BH4-rHPA groups, as expected.

Elevated phenylalanine is the hallmark of PKU, and, as expected, it is altered in patient samples. As Phe cannot be fully converted to tyrosine, the Phe/Tyr ratio is significantly different. Threonine is an essential amino acid that plays a role in protein synthesis, glycine generation, and mucin formation, which is crucial for gastrointestinal health and the gut microbiome [33]. As PKU patients follow a strict diet, this increase might be a secondary result of an altered gut microbiome. Threonine is also uptaken across the blood–brain barrier via the LAT1 transporter, and increased phenylalanine levels might competitively inhibit threonine. Differential taurine levels might be linked to an oxidative stress response, as it is upregulated as an antioxidant defense mechanism. This metabolite was previously studied in oligodendrocyte precursor cells in multiple sclerosis, and it was found that taurine levels increased by 20-fold during oligodendrocyte differentiation and maturation. It was recently reported that elevated Phe affects and impairs oligodendrocytes and contributes to myelin damage; therefore, changes in taurine levels [34] might be a secondary effect of Phe increases due to differences in taurine levels or due to dietary protein restriction. L-tryptophan is a large neutral amino acid that competes with Phe for transport across the blood–brain barrier, and when Phe is elevated in PKU, large neutral amino acid transporters are likely saturated and reduce the uptake of large neutral amino acids, which may explain the increased L-tryptophan levels. Chronic oxidative stress as a result of high levels of Phe exposure is an important factor in the pathophysiology of phenylketonuria (PKU). The altered aminocids L-cystine, L-glycine, L-proline, and L-asparagine are amino acids linked to oxidative stress. Therefore, oxidative stress in PKU could potentially impact the circulating levels of these metabolites.

In a recent study, kynurenine, tyrosine, asparagine, and proline were identified as differential amino acids between the PKU and control groups [35]. Our results agree with this study and expand the results with different subgroups of PKU and highlight the importance of quantitative measurements in PKU beyond the routine Phe and Phe/Tyr measurements.

In untargeted metabolomics experiments, glycyl-histidine, phenylalanyltyroptophan, d-phenyl lactic acid, d-phenylalanine, phenylpyruvic acid, n-(1-deoxy-1-fructosyl) phenylalanine, cinnamic acid, sphingosine 1-phosphate, L-kynurenine, glutamyl proline, and styrene metabolites were found in the PKU patient group.

D-phenyllactic acid, d-phenylalanine, and p-hydroxyphenyl acetic acid are characteristic metabolites found to be altered in PKU. These metabolites are involved in the phenylalanine synthesis and degradation pathways of phenylalanine. The detected Phe conjugation products of phenylalanyltyroptophan, d-phenyl lactic acid, and phenylpyruvic acid might be due to compensatory mechanisms to counterbalance the Phe toxicity and alternative catabolic routes.

Increased glycyl-histidine, a dipeptide, is probably caused by accumulated Phe or phenylpyruvate that might block dipeptidyl peptidases or aminopeptidases, which might block peptide cleavage. As glycine also increased in patients, that might also drive the formation of this dipeptide. Cinnamic acid and styrene are gut microbial metabolites that might be linked to impaired Phe metabolism [36,37]. Altered inflammatory states in PKU could potentially lead to dysregulation of S1P levels or receptor expression.

However, as the sample sizes of the moderate PKU and mild PKU were too low to allow for statistically meaningful results, we have not further done a stratified metabolomics analysis with CPKU, moderate PKU, and mild PKU samples.

In the mild HPA phenotype, cinnamic acid, n-(1-deoxy-1-fructosyl) phenylalanine, L-phenylalanine, arginylisoleucine, hydrocinnamic acid, sphingosine 1-phosphate, indole-3-carboxylic acid, valine, hydroxyprolylysine, L-tyrosine, phenylalanine-phenylalanine, gamma-glutamyltyrosine, 5-phenylpyruvic acid, glutamylproline, and 2-phenylbutyric acid, and in BH4-r-HPA patients, 13-OXODE, phenylacetylglycine, D-phenyllactic acid, glutamyltryptophan, phenylalanine-tryptophan, galabiosylceramide (d18:1/16:0), phenylalanine-proline, tyrosine, eicosapentaenoic acid, phenylacetic acid, cinnamic acid, glutamylproline, N-(1-deoxy-1-fructosyl) tyrosine, hydrocinnamic acid, and L-kynurenine metabolites showed differential alterations relative to healthy controls.

Similar to PKU, in both mild HPA patients and BH4-r-HPA patients, most of the metabolites are involved in the phenylalanine synthesis and degradation pathways of phenylalanine. Therefore, independent of the clinical phenotype, metabolites related to Phe metabolism are common in PKU. Tryptophan shares the same large neutral amino acid transporter (LAT1) to cross the blood–brain barrier as phenylalanine. High levels of phenylalanine can competitively inhibit tryptophan uptake into the brain, thereby affecting serotonin and melatonin synthesis. Therefore, this explains the enrichment of the tryptophan pathway.

Long-chain polyunsaturated fatty acids (LCPUFAs) are crucial for brain development and myelin formation. Phenylalanine build-up impacts fatty acid metabolism, possibly due to deficiencies in essential fatty acids (which can be exacerbated by dietary restrictions) or altered enzyme activities involved in their synthesis or desaturation. Tyrosine is a precursor for catecholamines (dopamine, norepinephrine, epinephrine), which are neurotransmitters and are also involved in steroid hormone regulation, so this might also explain the shared enrichment of the steroid hormone biosynthesis.

Some of these metabolites, namely glutamyl-Phe (Glu-Phe), phenylpyruvic acid, p-hydroxyphenylacetic acid, and n-lactoyl-phenylalanine, were previously reported in treated PKU patients [26]. In that report, n-lactoyl-phenylalanine was proposed as a better prognostic marker in adult PKU patients. However, we did not detect this metabolite in any of our subgroups, which might suggest that this might have a potential correlation with age, given that the average age in our patient cohort was 10 years. However, a controlled study comparing pediatric and adult PKU patient groups is necessary to definitively establish that n-lactoyl-phenylalanine is indeed an age-related biomarker.

We also found through K-means clustering analyses based on global metabolomics data that both PKU patients and mild HPA patients were clustering into two subgroups, but not in BH4-r-HPA. When we carefully inspected these patients, we found that this subclustering was correlated with their metabolic control status. A total of nine patients with PKU, four patients with mild HPA, but only one patient with BH4-r-HPA, were under poor metabolic control. Therefore, this explains why we see this trend only in PKU patients and mild HPA patients but not in BH4-r-HPA.

PKU patients were previously found to be grouped into different sub-metabolomic clusters based on their metabolic phenotyping. Our study is in partial agreement with that study as that study employed a targeted approach, whereas we used an untargeted approach that extends the number of metabolic features [22].

This result prompted us to investigate whether distinct and differential metabolites exist between these clusters. When we analyzed the metabolomics data with respect to metabolic control status compared to the control group, we found that in dietary non-adherent patients with poor metabolic control, n-(1-deoxy-1-fructosyl) phenylalanine was found to be increased with the highest VIP scores, whereas n-(1-deoxy-1-fructosyl) tyrosine was decreased. This was a particularly interesting finding, as n-(1-deoxy-1-fructosyl) phenylalanine metabolites were previously annotated as Phe-hexose adducts by infrared ion spectroscopy (IRIS) and MS and were linked to inborn errors of amino acid metabolism, including PKU [25]. These metabolites were termed as Amadori rearrangement products, formed through non-enzymatic reactions between fructose and amino acids. It was argued in that study that Amadori rearrangement products were readily formed under conditions of aminoacidemia, making them very relevant not only for PKU but also for other IEMs where amino acids steadily accumulate.

Based on our results, we hypothesize that in dietary non-adherent patients with poor metabolic control, elevated levels of excess Phe drive the formation of Amadori rearrangement products, even in normoglycemic conditions. Amadori rearrangement products are relatively stable metabolites but eventually give rise to advanced glycation end-product (AGE) formation over time under chronic metabolic stress. AGEs have long been as the primary hallmarks of chronic diseases, in particular diabetes, cardiovascular diseases, and neurodegenerative diseases. AGEs are pro-inflammatory molecules that can bind to specific receptors (RAGE), activating downstream signaling pathways that lead to the production of inflammatory cytokines. Phe-ARPs could therefore activate these pathways, contributing to chronic inflammation in PKU and oxidative stress [38,39], both of which play a significant role in the pathogenesis of PKU [40].

Clinically, this may have two major implications. First, ARP, especially N-(1-deoxy-1-fructosyl) phenylalanine, could serve as longer-term markers of metabolic control, analogous to HbA1c in diabetes, offering a complementary metric to episodic Phe measurements. Unlike phenylalanine, which reflects acute dietary compliance, ARPs may better represent cumulative exposure to metabolic imbalance and thus be more predictive of long-term outcomes. This could facilitate the stratification of PKU patients based on their risk of complications and their response to various therapeutic interventions. This could lead to tailored treatment protocols, where patients with higher ARP burdens might receive more aggressive dietary management or novel pharmacological therapies. Second, their formation may implicate oxidative stress and carbonyl stress as underlying pathophysiological mechanisms in PKU, contributing to neurotoxicity, inflammation, and possibly to the cognitive deficits observed in poorly controlled patients. The non-enzymatic attachment of hexose to phenylalanine could lead to adducts forming on proteins, particularly those with accessible amino groups. Such modifications can alter protein structure, stability, and, ultimately, their biological function. This could impact neurotransmitter synthesis, myelin formation, or other critical metabolic pathways, contributing to the neurocognitive and developmental issues observed in poorly controlled PKU patients.

While our results are promising due to the relatively small sample size of the patient cohort, they still need to be validated with a larger and longitudinal patient cohort with poor metabolic control to assess the true biological significance and clinical utility of these metabolites for PKU. This marker holds great potential to be correlated with neurocognitive scores, dietary adherence, and treatment response. ARPs are relatively stable intermediates, and their biochemical stability and detectability by mass spectrometry make them attractive candidates for inclusion in routine metabolomic panels for PKU monitoring.

We evaluated the lipidomics profiles of PKU patients to assess differences across three clinical phenotypes. Triglycerides, phosphatidylcholine, sphingomyelin, and lysophosphatidylcholine were the most abundant lipids identified. Although their relative proportions were consistent across phenotypes, triglycerides were significantly elevated in all groups, constituting approximately 50% of the total lipidome.

In PKU patients, differentially expressed lipids compared to controls included elevated triglycerides (TG 42:1, TG 44:00), diacylglycerols (DG 18:1/18:1, DG 18:0/18:0), phosphatidylcholines (PC 18:0/20:4, PC 16:0/18:0, PC 16:0/16:0), and lysophosphatidylcholine (LPC 20:2), with decreased LPC 18:2. In the hyperphenylalaninemia (HPA) group, elevated TG 46:00, DG 16:0/18:0, and DG 18:0/18:0 were observed. In tetrahydrobiopterin-responsive HPA (BH4-r-HPA) patients, elevated lipids included PC 16:0/18:2, PC 16:0/22:6, PC 18:0/18:2, sphingomyelins (SM d32:1, SM d34:2), LPC 18:0, LPC 18:2, TG 52:4, and TG 54:4.

We observed elevated levels of various triglyceride (TG) species containing both saturated and unsaturated fatty acyl chains in our study. Similarly, Weerd et al. [26] reported that TGs were the dominant lipid species, with significant increases in TG 45:5, 50:1, 50:2, 56:7, and 58:7, consistent with our findings. However, the mechanisms driving these TG alterations remain unclear. A previous prospective observational study [41] linked elevated triglyceride levels in PKU patients to dietary factors, noting that PKU patients often consume high-glycemic-index foods and tend to have higher BMI.

The BMI of our patients was 20.40 ± 4.98, 17.48 ± 3.51, and 20.09 ± 5.15 for PKU, HPA, and BH4-r-HPA, respectively, all within the normal range. Thus, we found no association between BMI and elevated triglyceride levels. An NMR-based metabolomics study reported low cholesterol levels in PKU patients, attributed to impaired cholesterol synthesis due to downregulated expression of 3-hydroxy-3-methylglutaryl coenzyme A reductase [42]. However, total triglyceride levels in that study were elevated, consistent with our findings.

It is notable that the lipoprotein profiles of PKU patients are non-atherogenic, and patients do not exhibit clinical triglyceridemia. One potential explanation is that excess phenylalanine (Phe) and its secondary metabolites induce oxidative stress, interfering with hepatic enzymes and altering lipid metabolism. PKU patients follow a strict diet that significantly reduces consumption of saturated and unsaturated fatty acids, particularly long-chain polyunsaturated fatty acids (LC-PUFAs). This dietary restriction may impair mitochondrial β-oxidation or upregulate lipogenesis, potentially contributing to increased triglyceride synthesis.

Phosphatidylcholines (PCs), lysophosphatidylcholines (LPCs), and ceramides were also altered in PKU patients. PCs, particularly those containing polyunsaturated fatty acids (PUFAs), were more abundant in PKU patients. LPCs, known as pro-inflammatory mediators, are influenced by metabolic stress and oxidative stress associated with elevated Phe levels [40]. Ceramides, generated in response to cellular stress, including oxidative stress, may contribute to the neurocognitive and systemic complications observed in PKU. In a recent work, ER stress has been proposed as a mechanism to cope with the Phe stress [43]. As PCs are vital for maintaining ER membrane health, their dysregulation can lead to ER stress. LPCs, particularly at elevated levels, act as toxic lipid metabolites that directly induce ER stress. Ceramides are strong inducers and mediators of ER stress, playing a central role in lipotoxicity and ER stress-induced cell death.

The formation of ARPs and AGEs in metabolomics parallels the elevation of pro-inflammatory lipids in lipidomics. Both contribute to chronic inflammation in PKU, driven by oxidative stress from Phe accumulation. AGEs and LPCs activate similar inflammatory pathways (e.g., RAGE, NF-κB), amplifying cytokine production and potentially exacerbating neurocognitive and systemic complications. This shared inflammatory response links the two datasets, highlighting a common pathophysiological mechanism.

Both metabolomic and lipidomic changes converge on pathways critical for brain function. Phe accumulation inhibits LNAA transport, reducing tryptophan and tyrosine availability for neurotransmitter synthesis, which contributes to cognitive deficits. Simultaneously, lipidomic alterations, particularly in PUFAs and ceramides, impair myelin formation and maintenance, exacerbating neurological damage. Taurine’s role in oligodendrocyte support links metabolomic and lipidomic findings, as oxidative stress from Phe accumulation affects both amino acid and lipid profiles, contributing to myelin damage.

We investigated whether lipidomics profiles could differentiate PKU patients with good versus poor metabolic control, but found a poor correlation, indicating that lipid profiles were not discriminative for metabolic control status. This lack of separation may be influenced by technical limitations inherent to lipidomics analyses. For instance, ion suppression in mass spectrometry-based lipidomics, where co-eluting compounds compete for ionization, could obscure subtle differences in lipid species between patient groups. This phenomenon may reduce the sensitivity for detecting low-abundance lipids or specific lipid classes, which might be critical to metabolic dysregulation in PKU. While our findings align with previous reports of altered lipid metabolism in PKU, more sensitive analytical methods, such as targeted lipidomics or integration of an additional separation, such as ion mobility, may be necessary to elucidate subtle differences associated with metabolic control. The other limitation we think is the relatively limited size of the dietary non-adherent patients, which might hinder the detection of subtle lipid differences associated with metabolic control.

## 4. Materials and Methods

### 4.1. Study Design Overview

In this study, 73 patients, of whom 29 had phenylketonuria, 3 had moderate PKU, 3 had mild PKU, 22 had mild hyperphenylalaninemia, and 16 had BH4-responsive hyperphenylalaninemia, with an age range of 0–18 years, followed up at Hacettepe University Pediatric Metabolism Clinics (Table S1), were included. For the control group, healthy pediatric plasma samples were obtained commercially (Innovative Research, Inc., Novi, MI, USA), and plasma samples were stored at −80 °C until the analysis (Table S2). A total of 20 controls for metabolomics and 10 for lipidomics were included, and characteristics are summarized in Table 1. Ethical board approval was obtained from the Hacettepe University Non-invasive Clinical Research Ethics Committee (GO 21/958), and informed consent was obtained from participants or their legal guardians. At admission, blood aromatic amino acid measurements (Phe, Tyr, Trp) were measured for all patients, and BH4 metabolism disorders were excluded by performing blood DHPR and urine pterine analyses. Dietary adherence was clinically assessed by evaluating neurological development and growth and by median plasma Phe levels. During the follow-up of patients, the median Phe value was calculated by excluding the highest and lowest values of blood Phe levels. Patients were classified according to their median blood Phe levels as poor metabolic control (median blood Phe > 6 mg/dL ≈ 360 µM) or good metabolic control (median blood Phe level < 4 mg/dL ≈ 120 µM). The borderline (Phe level < 4–6 mg/dL ≈ 120–360 µM) group is classified as part of the good metabolic control group.

### 4.2. Sample Collection and Plasma Extraction

Blood samples were collected from patients while fasting during outpatient clinic visits into Vacutainer^®^ EDTA tubes. After leaving 30 min at room temperature, plasma was isolated by centrifugation (10 min, 1800× *g*, 4 °C), aliquoted, and stored at −80 °C until analysis. Pooled quality control (QC) samples were prepared by mixing 10 µL of each plasma sample.

### 4.3. Metabolite Extraction from Plasma Samples

Plasma samples from patients, healthy controls, and pooled QC samples stored at −80 °C were thawed on ice. For metabolite extraction, 100 μL of each sample was taken into a new tube and 400 μL of LC-MS grade cold methanol:water (4:1) was added, vortexed for 5 min, and stored at −20 °C overnight. Samples were then sonicated in an ice bath for 60 min, centrifuged at +4 °C, 2000× *g* for 30 min, and the supernatant was dried in a vacuum concentrator. Dried samples were reconstituted in 100 μL methanol:water (1:9), of which 50 μL was taken into LC vials for analysis. Blank samples were prepared identically.

### 4.4. Lipid Extraction from Plasma Samples

The Matyash protocol [44] was used for lipid extraction. A 20 μL plasma sample was mixed with 225 μL cold methanol, vortexed for 10 s, then 750 μL MTBE was added, and the samples were shaken at 4 °C for 6 min. After adding 188 μL LC-MS grade water and vortexing for 20 s, samples were centrifuged (14,000× *g*, 2 min, room temperature). The upper lipid phase was transferred to a clean tube, dried in a vacuum concentrator, and reconstituted in 100 μL methanol:toluene (9:1). Finally, 50 μL was transferred to LC vials for analysis.

### 4.5. Characterization of Metabolite and Lipid Profiles by Liquid Chromatography–Hybrid Quadrupole Time-of-Flight Mass Spectrometry (LC–QTOF-MS)

Untargeted plasma metabolomics was performed using an Agilent 1290 UHPLC system coupled to an Agilent 6546 QTOF-MS with a dual ESI source. (Agilent Technologies, Santa Clara, CA, USA) Continuous lock-mass correction and <2 ppm mass accuracy were achieved by spraying the reference masses *m*/*z* 121.0509 (protonated purine) and *m*/*z* 922.0098 (protonated hexakis(1H,1H,3H-tetrafluoropropoxy) phosphazene (HP-921)) with the reference nebulizer. Metabolites were separated on an Acquity HSS T3 C18 column (2.1 mm × 100 mm, 1.8 µm) using (A) water:formic acid (100:0.1, *v*/*v*) and (B) methanol:water:formic acid (99:10:0.1, *v*/*v*/*v*). The gradient program was started with 1% B for 1 min, ramping to 100% B over 20 min, held for 4 min, returned to 1% B in 1 min, and equilibrated for 4 min. The flow rate was 0.4 mL/min. The Q-TOF was operated in positive ion mode with a 3000 V capillary voltage, 60 psi nebulizer pressure, and 225 °C drying gas temperature delivered at 10 L/min. Data were acquired in profile mode (40–1700 *m*/*z*, 0.33 s scan rate) and extended dynamic range setting. DDA MS/MS spectra were collected from QC samples (CE = 25 V, Top 5 ions). Lipidomics analyses were performed by an Acquity CSH C18 column (2.1 mm × 100 mm, 1.8 µm) with (A) acetonitrile:water:formic acid + 10 mM ammonium formate (60:40:0.1, *v*/*v*/*v*) and (B) isopropanol:acetonitrile:formic acid (90:10:0.1, *v*/*v*/*v*). The gradient program was started with 1% B for 1 min, ramping to 100% B over 30 min, held for 10 min, returned to 1% B in 1 min, and equilibrated for 5 min with a flow rate was 0.4 mL/min. The Q-TOF settings for lipidomics were identical to those of metabolomics analyses. MS/MS spectra were obtained from QC samples using data-dependent iterative analysis. Each batch included control, patient, pooled QC, and blank plasma samples. The experimental workflow is summarized in Figure 1. The analytical sequence started with sample solvent (3×), blank extraction sample (3×), pooled QC sample for chromatographic conditioning, patient samples, or healthy plasma samples. A pooled QC injection was performed every 5 samples so that possible analytical variations could be identified and corrected. To correct for possible run order and batch effects on the results, the order of patient and control samples in the analytical study was completely randomized.

### 4.6. Data Analysis

LC-MS raw data were first manually inspected in MassHunter Qualitative Analysis Software (V10.0), converted to mzML via MSConvert, and processed in MetaboAnalyst 6.0 using the CentWave-Auto algorithm with adducts: [M+H]+, [M+Na]+, [M+K]+, [M+NH4]+, [2M+H]+, [2M+Na]+, [2M+K]+, [3M+H]+, [3M+Na]+, [3M+K]+, [M+H-H2O]+, [M+H+HCOOH]+, [M+Na+HCOOH]+, [M+K+HCOOH]+. Data were analyzed statistically for multivariate and univariate comparisons. Filtering parameters: features with RSD > 20 in QC samples, variant filter: standard deviation (%10), abundance filter (%10). A blank filter was also applied. Normalization used sum, log10 transformation, and auto scaling. Data were modeled with PCA and with OPLS-DA to compare metabolic profiles between patients and controls and among different phenotypes. The most important metabolites were extracted from VIP plots (>1.5) and also visualized by heatmap based on *t*-test. This was followed by metabolic pathway analysis to identify relevant metabolites and pathways. The ROC analysis was conducted in using the biomarker analysis module. All plots were extracted from MetaboAnalyst 6.0 as SVG and modified by Inkscape (V 1.4.2). Lipidomics data were analyzed identically for statistical analyses. Additionally, MassHunter Lipid Annotator (Agilent Technologies V1.0) was used for classifying lipids by their identity and composition, and plots were extracted as SVG and modified by Inkscape.

### 4.7. Quantitative Amino Acid Analysis

Plasma samples from patients and controls were analyzed using an Agilent 1290 UHPLC system coupled to an Agilent 6470 QqQ mass spectrometer (Agilent Technologies, Santa Clara, CA, USA) with a dual ESI source. Sample preparation followed the JASEM Quantitative Amino Acids LC-MS/MS Kit (Altium International Lab. Cih. A.Ş., Istanbul, Turkiye) protocol: 50 µL of the sample was mixed with 50 µL internal standard, agitated for 5 s, combined with 700 µL reagent-1 for protein precipitation, and centrifuged at 3600× *g* for 5 min. The supernatant was injected into the LC-MS/MS system. Chromatographic separation of underivatized amino acids used the kit’s mobile phases, column, and binary gradient elution at 0.7 mL/min and 30 °C. The gradient started at 25% A for 1 min, increased to 75% B over 3 min, held for 0.5 min, and equilibrated at 25% A for 5 min, with a total run time of 12.5 min. Amino acids were detected in positive ion MRM mode with optimized mass spectrometer settings: drying gas at 120 °C and 10 L/min, nebulizer at 35 psi, sheath gas at 320 °C and 11 L/min, capillary voltage at 2200 V, and compound-specific fragmentation voltages and collision energies (Table S3). Data acquisition, qualification, and quantification used Agilent Mass Hunter Acquisition (v10.1), Qualitative Analysis (v10.0), and Quantitative Analysis (v10.0) software. Amino acid measurements were exported to Excel, assessed for distribution via histograms, and analyzed in MetaboAnalyst using the Kruskal–Wallis test. Results comparing amino acid concentrations in controls and patients are shown in Figure S1 and Table S4.

## 5. Conclusions

This study highlights significant differences in the lipidomic and metabolic profiles of different subgroups of PKU, namely classical PKU, mild HPA, and BH4-r-HPA patients, compared to healthy individuals. Our results exemplify the power of metabolomics and lipidomics to provide new insights into even well-studied diseases such as PKU. To the best of our knowledge, this is the first study that compares untargeted metabolic profiles of different PKU subtypes and their metabolic control status. We showed that untargeted metabolomics could reveal new putative metabolites that could potentially be associated with dietary adherence. We plan to test these new metabolites in a targeted fashion, both as standalone markers and as part of a broader panel, and compare them to canonical Phe and Phe/Tyr ratios. The main limitation of the study, however, was the limited size of the dietary non-adherent patients (n = 9). Another limitation is that we could not perform a further stratification of the PKU group, as the sample size of the moderate PKU and mild PKU subgroups (n = 3) was relatively low, and we analyzed them under a PKU group, as they all had Phe > 10. As a future plan, we will expand our cohort with a larger patient population to include different centers from Türkiye. It will also be interesting to conduct a longitudinal study to monitor the same patients at discrete time intervals, observing how the total metabolome profile and the newly identified metabolites change over time, and how and if they correlate with metabolic control status. As we have not been able to see n-lactoyl-phenylalanine, which is reported to be exclusively increased in adult PKU patients in our study, this requires future investigation to compare adult and pediatric PKU populations to evaluate whether this is an age-related biomarker for PKU.

## Figures and Tables

**Table 1:** Summary of cohort characteristics.

Cohort Characteristics	PKU	HPA	BH4-Responsive HPA
n	35 (29 classical PKU, 3 moderate PKU, 3 mild PKU)	22 mild HPA	16
Age	10.29 ± 5.84 (IQR: 4.45–16.12)	7.64 ± 4.08 (IQR: 3.55–11.72)	8.5 ± 3.87 (IQR: 4.63–12.37)
BMI	20.40 ± 4.98 (IQR: 15.42–25.37)	17.48 ± 3.51 (IQR: 13.97–20.99)	20.09 ± 5.15 (IQR: 14.94–25.24)
Gender	19 Males, 16 Females	10 Males, 12 Females	8 Males, 8 Females
Metabolic Control status	26 (Good) 9 (Poor)	18 (Good) 4 (Poor)	15 (Good) 1 (Poor)
Phe levels at diagnosis (µM)	1574.5 ± 686.4 (IQR: 888.67–2261.0)	254.8 ± 93.8 (IQR: 161.0–348.7)	406.80 ± 151.3 (IQR: 256.1–558.14)
BW	41.98 ± 20.23 (IQR: 21.75–62.21)	27.31 ± 14.15 (13.17–41.46)	35.52 ± 19.90 (IQR: 15.62–55.42)
Height	138.44 ± 24.31 (IQR: 114.13–162.75)	120.95 ± 25.71 (IQR: 95.25–146.66)	127.91 ± 26.56 (IQR: 101.35–154.47)

## Data Availability

The data presented in this study are available upon reasonable request from the corresponding author, subject to written approval by the ethical board for data sharing. Due to patient privacy and ethical board restrictions, the data cannot be made publicly available.

## Supplementary Materials

The following supporting information can be downloaded at: https://www.mdpi.com/article/10.3390/ijms26157171/s1.

## Abbreviations

PKU	Phenylketonuria
HPA	Hyperphenylalaninemia
BH4-r-HPA	Tetrahydrobiopterin-Responsive Hyperphenylalaninemia
CPKU	Classical Phenylketonuria
Phe	Phenylalanine
Tyr	Tyrosine
Trp	Tryptophan
Phe/Tyr	Phenylalanine to Tyrosine ratio
BMI	Body Mass Index
BW	Body Weight
IQR	Interquartile Range
SD	Standard Deviation
LC-MS/MS	Liquid Chromatography–Mass Spectrometry
Q-TOF	Quadrupole Time-of-Flight
CE	Collision Energy
DDA	Data-Dependent Analysis
PCA	Principal Component Analysis
PLS-DA	Partial Least Squares Discriminant Analysis
OPLS-DA	Orthogonal Partial Least Squares Discriminant Analysis
VIP	Variable Importance in Projection
MS	Mass Spectrometry
NMR	Nuclear Magnetic Resonance
ESI	Electrospray Ionization
QC	Quality Control
TG	Triglyceride
DG	Diglyceride
PC	Phosphatidylcholine
LPC	Lysophosphatidylcholine
SM	Sphingomyelin
PUFA	Polyunsaturated Fatty Acids
AGE	Advanced Glycation End Products
ARPs	Amadori Rearrangement Products
HbA1c	Hemoglobin A1c (glycosylated hemoglobin)
IRIS	Infrared Ion Spectroscopy
MRM	Multiple Reaction Monitoring
UHPLC	Ultra-High-Performance Liquid Chromatography
*v*/*v*	Volume/Volume
*m*/*z*	Mass-to-Charge-Ratio

## References

[1] Elhawary N.A., AlJahdali I.A., Abumansour I.S., Elhawary E.N., Gaboon N., Dandini M., Madkhali A., Alosaimi W., Alzahrani A., Aljohani F. (2022). Genetic etiology and clinical challenges of phenylketonuria. Hum. Genom..

[2] Opladen T., López-Laso E., Cortès-Saladelafont E., Pearson T.S., Sivri H.S., Yildiz Y., Assmann B., Kurian M.A., Leuzzi V., Heales S. (2020). Consensus guideline for the diagnosis and treatment of tetrahydrobiopterin (BH4) deficiencies. Orphanet J. Rare Dis..

[3] Güttler F., Lou H., Fernandes J., Saudubray J.-M., Tada K. (1990). Phenylketonuria and Hyperphenylalaninemia. Inborn Metabolic Diseases: Diagnosis and Treatment.

[4] Guldberg P., Rey F., Zschocke J., Romano V., François B., Michiels L., Ullrich K., Hoffmann G.F., Burgard P., Schmidt H. (1998). A European multicenter study of phenylalanine hydroxylase deficiency: Classification of 105 mutations and a general system for genotype-based prediction of metabolic phenotype. Am. J. Hum. Genet..

[5] van Wegberg A.M.J., MacDonald A., Ahring K., Bélanger-Quintana A., Beblo S., Blau N., Bosch A.M., Burlina A., Campistol J., Coşkun T. (2025). European guidelines on diagnosis and treatment of phenylketonuria: First revision. Mol. Genet. Metab..

[6] Evers R.A.F., van Vliet D., van Spronsen F.J. (2020). Tetrahydrobiopterin treatment in phenylketonuria: A repurposing approach. J. Inherit. Metab. Dis..

[7] Moat S.J., George R.S., Carling R.S. (2020). Use of Dried Blood Spot Specimens to Monitor Patients with Inherited Metabolic Disorders. Int. J. Neonatal Screen..

[8] McWhorter N., Ndugga-Kabuye M.K., Puurunen M., Ernst S.L. (2022). Complications of the Low Phenylalanine Diet for Patients with Phenylketonuria and the Benefits of Increased Natural Protein. Nutrients.

[9] van Spronsen F.J., Blau N., Harding C., Burlina A., Longo N., Bosch A.M. (2021). Phenylketonuria. Nat. Rev. Dis. Primers.

[10] Rovelli V., Longo N. (2023). Phenylketonuria and the brain. Mol. Genet. Metab..

[11] Burlina A.P., Cazzorla C., Massa P., Polo G., Loro C., Gueraldi D., Burlina A.B. (2019). Large Neutral Amino Acid Therapy Increases Tyrosine Levels in Adult Patients with Phenylketonuria: A Long-Term Study. Nutrients.

[12] van Vliet D., van der Goot E., van Ginkel W.G., van Faassen H.J.R., de Blaauw P., Kema I.P., Heiner-Fokkema M.R., van der Zee E.A., van Spronsen F.J. (2022). The increasing importance of LNAA supplementation in phenylketonuria at higher plasma phenylalanine concentrations. Mol. Genet. Metab..

[13] Thau-Zuchman O., Pallier P.N., Savelkoul P.J.M., Kuipers A.A.M., Verkuyl J.M., Michael-Titus A.T. (2022). High phenylalanine concentrations induce demyelination and microglial activation in mouse cerebellar organotypic slices. Front. Neurosci..

[14] Rondelli V., Koutsioubas A., Di Cola E., Fragneto G., Grillo I., Del Favero E., Colombo L., Cantù L., Brocca P., Salmona M. (2022). Dysmyelination and glycolipid interference caused by phenylalanine in phenylketonuria. Int. J. Biol. Macromol..

[15] van Spronsen F.J., Hoeksma M., Reijngoud D.-J. (2009). Brain dysfunction in phenylketonuria: Is phenylalanine toxicity the only possible cause?. J. Inherit. Metab. Dis..

[16] Dobrowolski S.F., Phua Y.L., Vockley J., Goetzman E., Blair H.C. (2022). Phenylketonuria oxidative stress and energy dysregulation: Emerging pathophysiological elements provide interventional opportunity. Mol. Genet. Metab..

[17] Moat S.J., Schulenburg-Brand D., Lemonde H., Bonham J.R., Weykamp C.W., Mei J.V., Shortland G.S., Carling R.S. (2020). Performance of laboratory tests used to measure blood phenylalanine for the monitoring of patients with phenylketonuria. J. Inherit. Metab. Dis..

[18] Feldmann R., Schallert M., Nguyen T., Och U., Rutsch F., Weglage J. (2019). Children and adolescents with phenylketonuria display fluctuations in their blood phenylalanine levels. Acta Paediatr..

[19] Wada Y., Totsune E., Mikami-Saito Y., Kikuchi A., Miyata T., Kure S. (2023). A method for phenylalanine self-monitoring using phenylalanine ammonia-lyase and a pre-existing portable ammonia detection system. Mol. Genet. Metab. Rep..

[20] Messina M.A., Maugeri L., Spoto G., Puccio R., Ruggieri M., Petralia S. (2023). Fully Integrated Point-of-Care Platform for the Self-Monitoring of Phenylalanine in Finger-Prick Blood. ACS Sens..

[21] Wild J., Shanmuganathan M., Hayashi M., Potter M., Britz-McKibbin P. (2019). Metabolomics for improved treatment monitoring of phenylketonuria: Urinary biomarkers for non-invasive assessment of dietary adherence and nutritional deficiencies. Analyst.

[22] Moritz L., Klotz K., Grünert S.C., Hannibal L., Spiekerkoetter U. (2023). Metabolic phenotyping in phenylketonuria reveals disease clustering independently of metabolic control. Mol. Genet. Metab..

[23] Xiong X., Sheng X., Liu D., Zeng T., Peng Y., Wang Y. (2015). A GC/MS-based metabolomic approach for reliable diagnosis of phenylketonuria. Anal. Bioanal. Chem..

[24] Cannet C., Bayat A., Frauendienst-Egger G., Freisinger P., Spraul M., Himmelreich N., Kockaya M., Ahring K., Godejohann M., MacDonald A. (2023). Phenylketonuria (PKU) Urinary Metabolomic Phenotype Is Defined by Genotype and Metabolite Imbalance: Results in 51 Early Treated Patients Using Ex Vivo 1H-NMR Analysis. Molecules.

[25] van Outersterp R.E., Moons S.J., Engelke U.F.H., Bentlage H., Peters T.M.A., van Rooij A., Huigen M.C.D.G., de Boer S., van der Heeft E., Kluijtmans L.A.J. (2021). Amadori rearrangement products as potential biomarkers for inborn errors of amino-acid metabolism. Commun. Biol..

[26] Weerd J.C.V., Wegberg A., Boer T.S., Engelke U.F.H., Coene K.L.M., Wevers R.A., Bakker S.J.L., Blaauw P., Groen J., Spronsen F.J.V. (2024). Impact of Phenylketonuria on the Serum Metabolome and Plasma Lipidome: A Study in Early-Treated Patients. Metabolites.

[27] van Wegberg A.M.J., van der Weerd J.C., Engelke U.F.H., Coene K.L.M., Jahja R., Bakker S.J.L., Huijbregts S.C.J., Wevers R.A., Heiner-Fokkema M.R., van Spronsen F.J. (2024). The clinical relevance of novel biomarkers as outcome parameter in adults with phenylketonuria. J. Inherit. Metab. Dis..

[28] Dos Santos Y., Emond P., Schwartz I.V.D., Lefèvre A., Dupuy C., Chicheri G., Blasco H., Maillot F. (2025). Multimodal Metabolomic Analysis Reveals Novel Metabolic Disturbances in Adults with Early Treated Phenylketonuria. JIMD Rep..

[29] Stroup B.M., Nair N., Murali S.G., Broniowska K., Rohr F., Levy H.L., Ney D.M. (2018). Metabolomic Markers of Essential Fatty Acids, Carnitine, and Cholesterol Metabolism in Adults and Adolescents with Phenylketonuria. J. Nutr..

[30] Mütze U., Beblo S., Kortz L., Matthies C., Koletzko B., Bruegel M., Rohde C., Thiery J., Kiess W., Ceglarek U. (2012). Metabolomics of Dietary Fatty Acid Restriction in Patients with Phenylketonuria. PLoS ONE.

[31] Guerra I.M.S., Diogo L., Pinho M., Melo T., Domingues P., Domingues M.R., Moreira A.S.P. (2021). Plasma Phospholipidomic Profile Differs between Children with Phenylketonuria and Healthy Children. J. Proteome Res..

[32] Guerra I.M.S., Ferreira H.B., Neves B., Melo T., Diogo L.M., Domingues M.R., Moreira A.S.P. (2020). Lipids and phenylketonuria: Current evidences pointed the need for lipidomics studies. Arch. Biochem. Biophys..

[33] Sanjurjo P., Aldamiz L., Georgi G., Jelinek J., Ruiz J.I., Boehm G. (2003). Dietary threonine reduces plasma phenylalanine levels in patients with hyperphenylalaninemia. J. Pediatr. Gastroenterol. Nutr..

[34] Beyer B.A., Fang M., Sadrian B., Montenegro-Burke J.R., Plaisted W.C., Kok B.P.C., Saez E., Kondo T., Siuzdak G., Lairson L.L. (2018). Metabolomics-based discovery of a metabolite that enhances oligodendrocyte maturation. Nat. Chem. Biol..

[35] Matuszewska E., Matysiak J., Kałużny Ł., Walkowiak D., Plewa S., Duś-Żuchowska M., Rzetecka N., Jamka M., Klupczyńska-Gabryszak A., Piorunek M. (2024). Amino Acid Profile Alterations in Phenylketonuria: Implications for Clinical Practice. Metabolites.

[36] Pinheiro de Oliveira F., Mendes R.H., Dobbler P.T., Mai V., Pylro V.S., Waugh S.G., Vairo F., Refosco L.F., Roesch L.F.W., Schwartz I.V.D. (2016). Phenylketonuria and Gut Microbiota: A Controlled Study Based on Next-Generation Sequencing. PLoS ONE.

[37] Mancilla V.J., Mann A.E., Zhang Y., Allen M.S. (2021). The Adult Phenylketonuria (PKU) Gut Microbiome. Microorganisms.

[38] Martin M.S., Jacob-Dolan J.W., Pham V.T.T., Sjoblom N.M., Scheck R.A. (2024). The chemical language of protein glycation. Nat. Chem. Biol..

[39] Santos H.O., Penha-Silva N. (2022). Translating the advanced glycation end products (AGEs) knowledge into real-world nutrition strategies. Eur. J. Clin. Nutr..

[40] Wyse A.T.S., dos Santos T.M., Seminotti B., Leipnitz G. (2021). Insights from Animal Models on the Pathophysiology of Hyperphenylalaninemia: Role of Mitochondrial Dysfunction, Oxidative Stress and Inflammation. Mol. Neurobiol..

[41] Yılmaz B.K., Baykan A., Kardaş F., Kendirci M. (2023). Evaluation of the effect of obesity, dietary glycemic index and metabolic profiles on the cardiovascular risk in children with classical phenylketonuria. Mol. Genet. Metab..

[42] Cannet C., Pilotto A., Rocha J.C., Schäfer H., Spraul M., Berg D., Nawroth P., Kasperk C., Gramer G., Haas D. (2020). Lower plasma cholesterol, LDL-cholesterol and LDL-lipoprotein subclasses in adult phenylketonuria (PKU) patients compared to healthy controls: Results of NMR metabolomics investigation. Orphanet J. Rare Dis..

[43] Gürses Cila H.E., Dursun A., Vardar Acar N., Geçici N.N., Ayhan S., Oskay Halaçlı S., Özgül R.K. (2025). Endoplasmic reticulum stress pathways and cellular death mechanisms in patients with phenylketonuria. Mol. Biol. Rep..

[44] Matyash V., Liebisch G., Kurzchalia T.V., Shevchenko A., Schwudke D. (2008). Lipid extraction by methyl-tert-butyl ether for high-throughput lipidomics. J. Lipid Res..

