# Supplementary material for: Metabolomics and Lipidomics Explore Phenotype-Specific Molecular Signatures for Phenylketonuria
Source: Int J Mol Sci. 2025 Jul 25;26(15):7171. doi: 10.3390/ijms26157171 (PMC12346085; doi:10.3390/ijms26157171)
Supplement: Supplementary file 1 [file ijms-26-07171-s001.zip › ijms-3737803_SI1_proofreading.docx]

**Table S1. List and characteristics of patients included in the study.**

| **Patient**  **Number** | **Age  (years)** | **Gender** | **Clinical**  **Phenotype** | **BW  (kg)** | **Height   (cm)** | **BMI  (kg/m2)** | **Metabolic**  **Control** | **Median Phe**  **(µM)** | **Sample Collection Date Phe levels** |
| --- | --- | --- | --- | --- | --- | --- | --- | --- | --- |
| 1 | 11 | Female | PKU | 73,1 | 160 | 28,55 | No | 750.6 | 961.8 |
| 2 | 2 | Male | PKU | 11,3 | 80,8 | 17,3 | Yes | 148.3 | 229.4 |
| 3 | 13 | Male | PKU | 61,8 | 173 | 20,65 | Yes | 315.4 | 432.8 |
| 4 | 5 | Male | PKU | 21,25 | 117 | 16,07 | Yes | 145.3 | 79.9 |
| 5 | 2 | Female | PKU | 60 | 156 | 24,65 | Yes | 217.9 | 610.8 |
| 6 | 16 | Female | PKU | 49,2 | 149,5 | 22,01 | Yes | 309.9 | 487.3 |
| 7 | 9 | Male | PKU | 24,5 | 130,5 | 14.38 | Yes | 182.8 | 220.9 |
| 8 | 5 | Male | PKU | 23,9 | 118 | 17,16 | Yes | 336.6 | 262.7 |
| 9 | 14 | Male | PKU | 47,6 | 159,5 | 18,71 | Yes | 135.6 | 294.2 |
| 10 | 3 | Male | PKU | 15,7 | 101 | 15,39 | Yes | 213.7 | 287.5 |
| 11 | 18 | Male | PKU | 51.3 | 169 | 17,96 | Yes | 283.3 | 738.5 |
| 12 | 18 | Female | PKU | 40,6 | 154 | 17,12 | Yes | 199.1 | 61.7 |
| 13 | 4 | Male | PKU | 18,1 | 100,5 | 17.92 | Yes | 180.4 | 389.2 |
| 14 | 7 | Male | PKU | 23,4 | 115 | 17,69 | No | 577.5 | 742.1 |
| 15 | 2 | Female | PKU | 41,9 | 153 | 17,9 | No | 741.5 | 708.8 |
| 16 | 18 | Female | PKU | 66 | 147 | 30,54 | Yes | 342.6 | 281.5 |
| 17 | 18 | Male | PKU | 61 | 157 | 24,75 | Yes | 301.4 | 1360.1 |
| 18 | 18 | Female | PKU | 47,2 | 145,5 | 22,29 | No | 729.4 | 487.9 |
| 19 | 13 | Male | PKU | 50 | 152,5 | 21,45 | No | 955.2 | 331.1 |
| 20 | 18 | Female | PKU | 93,5 | 162 | 35,62 | No | 727.6 | 851.7 |
| 21 | 10 | Female | PKU | 58 | 146,5 | 27,02 | No | 620.4 | 457.6 |
| 22 | 4 | Female | PKU | 16,8 | 109,5 | 14,01 | Yes | 174.3 | 75.7 |
| 23 | 18 | Erkek | PKU | 43,7 | 135,5 | 23,8 | Yes | 216.7 | 846.2 |
| 24 | 10 | Female | PKU | 63 | 170,5 | 21,67 | No | 693.1 | 846.2 |
| 25 | 10 | Female | PKU | 28 | 132,5 | 15.94 | No | 632.5 | 262.1 |
| 26 | 3 | Female | PKU | 24,5 | 125 | 17,51 | Yes | 135.0 | 236.7 |
| 27 | 4 | Female | PKU | 13,94 | 96,7 | 14,91 | Yes | 119.9 | 515.7 |
| 28 | 4 | Male | PKU | 43 | 160 | 16,79 | Yes | 208.8 | 170.7 |
| 29 | 8 | Male | PKU | 19,3 | 104 | 17,84 | Yes | 154.4 | 294.2 |
| 30 | 15 | Male | PKU | 43,9 | 140 | 22,39 | Yes | 335.3 | 701.6 |
| 31 | 18 | Male | PKU | 74 | 163 | 27.85 | Yes | 266.9 | 370.4 |
| 32 | 10 | Male | PKU | 56,3 | 160 | 21,99 | Yes | 216.1 | 41.8 |
| 33 | 5 | Female | PKU | 35 | 141 | 17,6 | Yes | 148.3 | 282.7 |
| 34 | 9 | Male | PKU | 16,45 | 106,5 | 14,5 | Yes | 245.3 | 456.5 |
| 35 | 18 | Female | PKU | 52 | 154 | 21,92 | Yes | 332.3 | 1014.5 |
| 36 | 2 | Female | MILD HPA | 16.38 | 84.5 | 14.4 | No | 458.8 | 306.9 |
| 37 | 2 | Female | MILD HPA | 40.5 | 83 | 23.77 | Yes | 200.4 | 242.7 |
| 38 | 9 | Male | MILD HPA | 23 | 128 | 24.71 | Yes | 277.8 | 164.6 |
| 39 | 10 | Female | MILD HPA | 28.7 | 126.5 | 14.37 | Yes | 308.7 | 278.4 |
| 40 | 10 | Male | MILD HPA | 25.8 | 140 | 14.64 | Yes | 257.3 | 122.9 |
| 41 | 10 | Male | MILD HPA | 21.8 | 130 | 16.26 | No | 487.3 | 121.7 |
| 42 | 8 | Male | MILD HPA | 16.8 | 119 | 15.39 | Yes | 245.2 | 272.4 |
| 43 | 3 | Female | MILD HPA | 17.2 | 101 | 16.47 | Yes | 193.1 | 130.7 |
| 44 | 4 | Female | MILD HPA | 17.5 | 102.5 | 16.37 | Yes | 155.0 | 194.9 |
| 45 | 6 | Male | MILD HPA | 62.1 | 115 | 13.23 | No | 455.8 | 294.8 |
| 46 | 13 | Female | MILD HPA | 9.5 | 167 | 22.26 | Yes | 156.2 | 105.9 |
| 47 | 2 | Female | MILD HPA | 12.13 | 79 | 15.22 | Yes | 214.9 | 168.3 |
| 48 | 2 | Female | MILD HPA | 53 | 88 | 15.88 | Yes | 289.9 | 353.5 |
| 49 | 14 | Female | MILD HPA | 31.6 | 154.5 | 22.2 | Yes | 343.8 | 315.4 |
| 50 | 8 | Female | MILD HPA | 22 | 121 | 21.58 | No | 559.3 | 285.7 |
| 51 | 9 | Male | MILD HPA | 18.9 | 121.5 | 14.9 | Yes | 130.1 | 93.8 |
| 52 | 13 | Male | MILD HPA | 43 | 164 | 15.98 | Yes | 115.56 | 190.12 |
| 53 | 13 | Male | MILD HPA | 43 | 164 | 15.98 | Yes | 192.20 | 158.55 |
| 54 | 14 | Male | MILD HPA | 34.2 | 146 | 16.05 | Yes | 155.23 | 299.21 |
| 55 | 10 | Male | MILD HPA | 46 | 141 | 23.13 | Yes | 116.88 | 300.12 |
| 56 | 10 | Female | MILD HPA | 37 | 144 | 17.84 | Yes | 265.89 | 222.52 |
| 57 | 2 | Female | MILD HPA | 12.8 | 90.5 | 15.62 | Yes | 348.56 | 300.56 |
| 58 | 6 | Female | BH4 responsive HPA | 25 | 121,5 | 16,93 | Yes | 122.3 | 728.8 |
| 59 | 9 | Female | BH4 responsive HPA | 49 | 145 | 23,3 | Yes | 194.9 | 173.1 |
| 60 | 4 | Male | BH4 responsive HPA | 15,5 | 98 | 16,13 | Yes | 272.4 | 242.1 |
| 61 | 11 | Male | BH4 responsive HPA | 33,65 | 145 | 15,14 | Yes | 222.8 | 174.9 |
| 62 | 14 | Female | BH4 responsive HPA | 49,95 | 159,5 | 19,63 | Yes | 249.4 | 181.6 |
| 63 | 11 | Male | BH4 responsive HPA | 43,25 | 142 | 21,44 | Yes | 132.0 | 171.3 |
| 64 | 6 | Male | BH4 responsive HPA | 24,5 | 116,5 | 18,05 | Yes | 294.8 | 247.6 |
| 65 | 6 | Male | BH4 responsive HPA | 20 | 121,5 | 13,55 | Yes | 120.5 | 168.9 |
| 66 | 2 | Male | BH4 responsive HPA | 7,12 | 60,5 | 19,45 | No | 535.7 | 851.7 |
| 67 | 5 | Female | BH4 responsive HPA | 19,5 | 112 | 15,54 | Yes | 121.7 | 75.7 |
| 68 | 4 | Female | BH4 responsive HPA | 26,9 | 100,5 | 26,63 | Yes | 143.5 | 166.5 |
| 69 | 11 | Male | BH4 responsive HPA | 31,2 | 137 | 16,62 | Yes | 280.9 | 301.4 |
| 70 | 13 | Female | BH4 responsive PKU | 48,1 | 160 | 18.78 | Yes | 144.1 | 104.1 |
| 71 | 15 | Female | BH4 responsive HPA | 94 | 164 | 34.94 | Yes | 122.55 | 155.20 |
| 72 | 12 | Male | BH4 responsive HPA | 51 | 146 | 23.92 | Yes | 120.25 | 140.21 |
| 73 | 13 | Female | BH4 responsive PKU | 48.1 | 160 | 18.78 | Yes | 122.88 | 145.32 |

| **Table S2.** List and characteristics of healthy pediatric plasma samples included in the study. | | |
| --- | --- | --- |
| **Control Number** | **Age** | **Gender** |
| K1 | 1 | Female |
| K2 | 1 | Male |
| K3 | 2 | Female |
| K4 | 3 | Female |
| K5 | 6 | Male |
| K6 | 4 | Male |
| K7 | 5 | Female |
| K8 | 6 | Male |
| K9 | 10 | Female |
| K10 | 10 | Male |
| K11 | 9 | Male |
| K12 | 8 | Female |
| K13 | 12 | Female |
| K14 | 14 | Female |
| K15 | 11 | Male |
| K16 | 13 | Male |
| K17 | 16 | Male |
| K18 | 16 | Female |
| K19 | 17 | Male |
| K20 | 15 | Female |

**Table S3.** **MRM conditions of amino acids of the JASEM AA kit**

| Compound Name | Precursor Ion (m/z) | Product Ion (m/z) | FV^1^ (V) | CE^2^ (V) | Polarity |
| --- | --- | --- | --- | --- | --- |
| 2-Aminoadipic acid | 162.0 | 98 | 90 | 10 | Positive |
| 2-Aminobutyric acid | 104.2 | 58.3 | 80 | 4 | Positive |
| 3-Aminoisobutyric acid | 104.1 | 86.2 | 100 | 2 | Positive |
| 1-Methylhistidine | 170.1 | 124.1 | 100 | 10 | Positive |
| 3-Methylhistidine | 170.1 | 126.2 | 120 | 10 | Positive |
| Alanine | 90.2 | 44.2 | 80 | 4 | Positive |
| Arginine | 175.2 | 70.2 | 110 | 20 | Positive |
| Argininosuccinic acid | 291.0 | 70.2 | 140 | 30 | Positive |
| Asparagine | 133.1 | 74.2 | 70 | 10 | Positive |
| beta-Alanine | 90.1 | 72.2 | 80 | 2 | Positive |
| Carnosine | 227.1 | 110.1 | 110 | 22 | Positive |
| Citrulline | 176.2 | 159.3 | 80 | 3 | Positive |
| Cystathionine | 223.0 | 134.0 | 100 | 8 | Positive |
| Cystine | 241.1 | 74.2 | 100 | 24 | Positive |
| Ethanolamine | 62.1 | 44.2 | 80 | 4 | Positive |
| gamma-Aminobutyric acid | 104.0 | 87.1 | 80 | 6 | Positive |
| Glutamic acid | 148.1 | 84.2 | 80 | 12 | Positive |
| Glutamine | 147.1 | 84.2 | 80 | 12 | Positive |
| Glycine | 76.2 | 30.1 | 80 | 1 | Positive |
| Histidine | 156.1 | 110.1 | 100 | 8 | Positive |
| Hydroxylysine | 163.1 | 128.1 | 90 | 6 | Positive |
| Isoleucine | 132.2 | 69.2 | 100 | 14 | Positive |
| Leucine | 132.2 | 43.3 | 100 | 24 | Positive |
| Lysine | 147.1 | 84.2 | 80 | 12 | Positive |
| Methionine | 150.1 | 104.1 | 80 | 4 | Positive |
| Ornithine | 133.2 | 70.3 | 80 | 14 | Positive |
| Phenylalanine | 166.1 | 120.1 | 80 | 6 | Positive |
| Proline | 116.2 | 70.2 | 90 | 12 | Positive |
| Sarcosine | 90.1 | 44.2 | 90 | 8 | Positive |
| Serine | 106.2 | 60.2 | 80 | 4 | Positive |
| Taurine | 126.1 | 44.3 | 110 | 14 | Positive |
| Threonine | 120.2 | 74.2 | 80 | 4 | Positive |
| trans-4-Hydroxyproline | 132.2 | 68.2 | 90 | 20 | Positive |
| Tryptophan | 205.1 | 188.1 | 80 | 1 | Positive |
| Tyrosine | 182.1 | 165 | 80 | 1 | Positive |
| Valine | 118.2 | 72.2 | 80 | 4 | Positive |

^1^ FV: Fragmentor voltage,^2^ CE: Collision energy

**Figure S1. Box-Plots of measured amino acids by quantitative LC-MS/MS analyses**


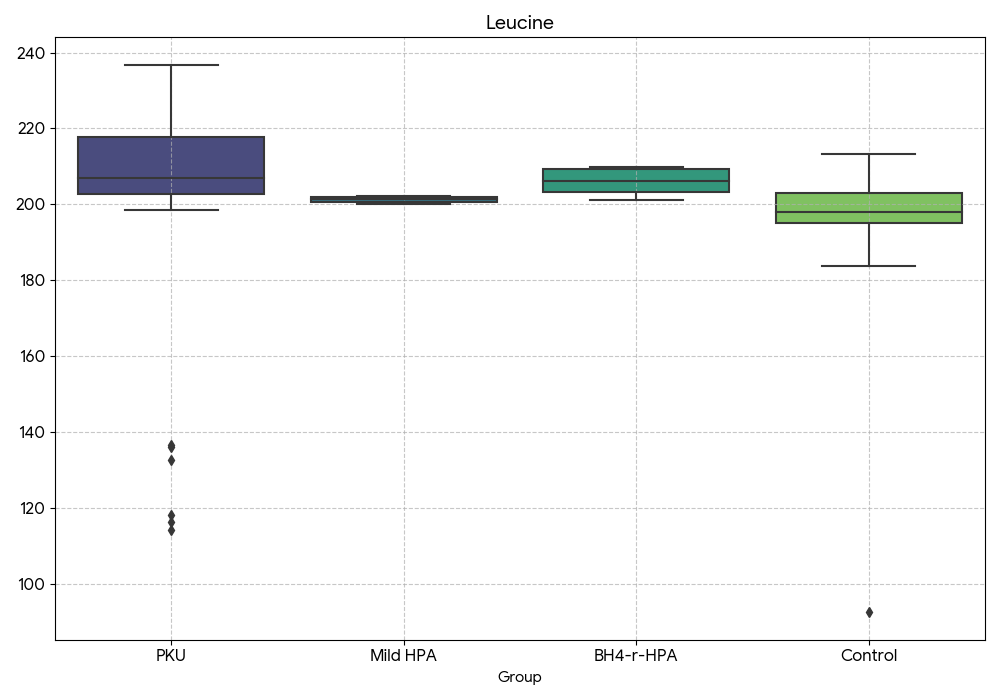


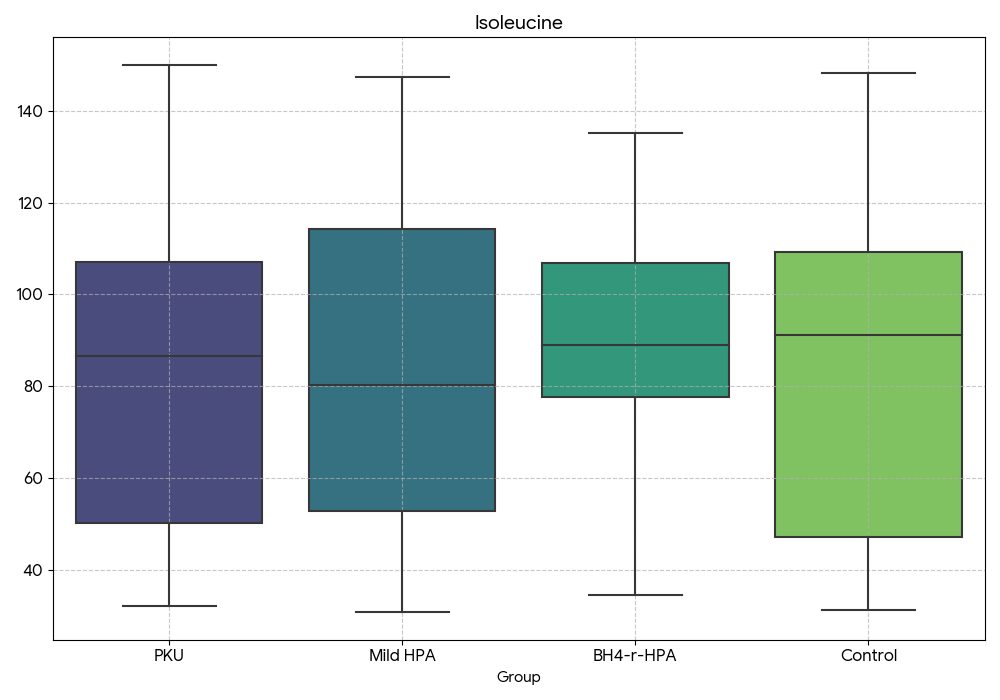


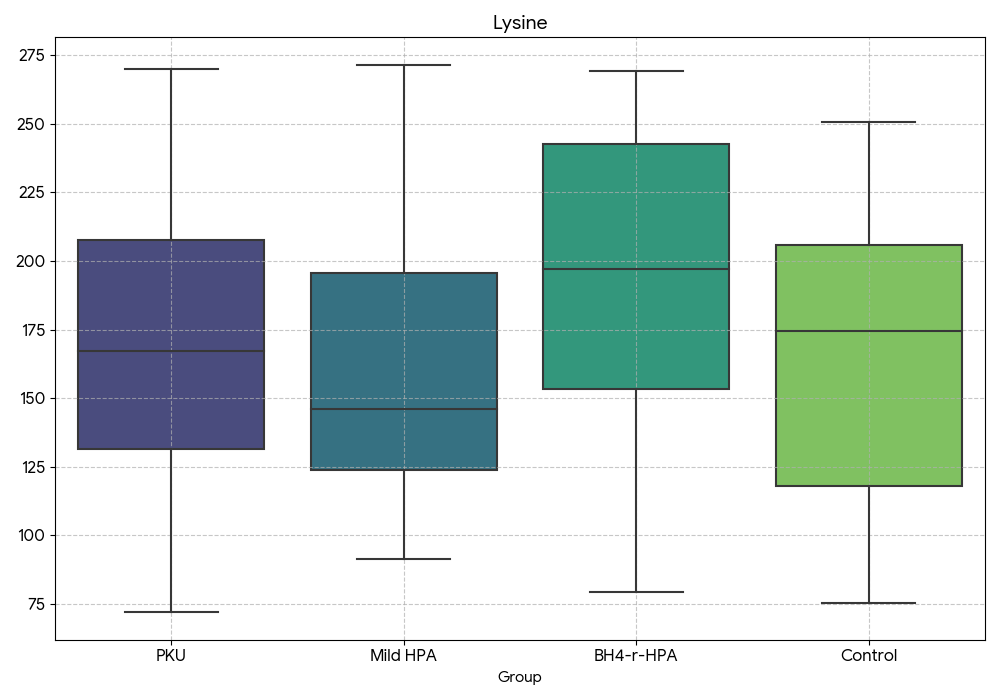


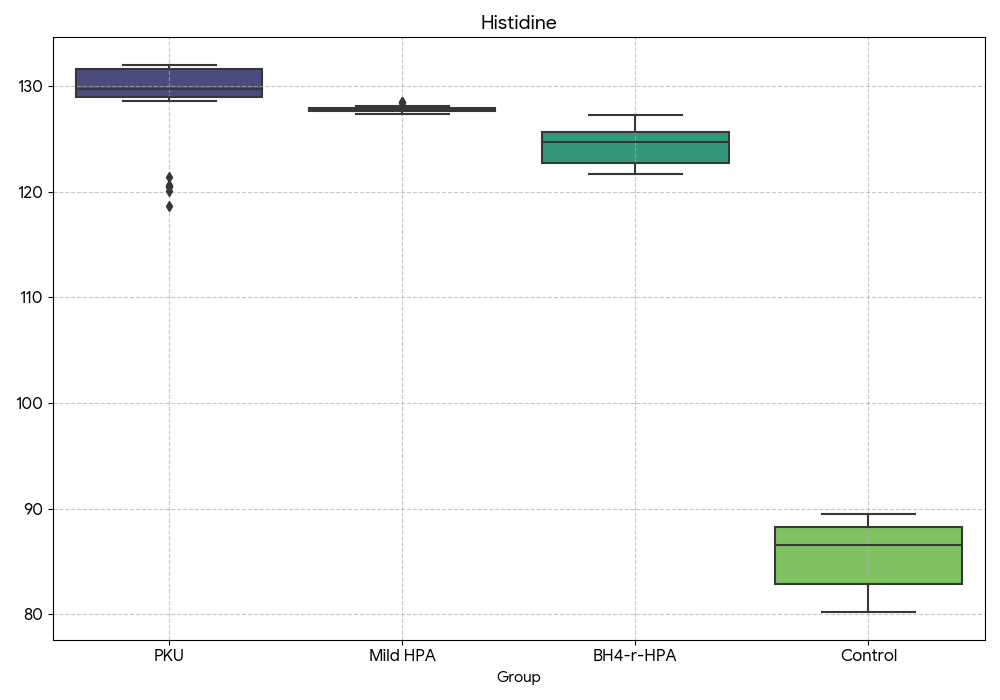


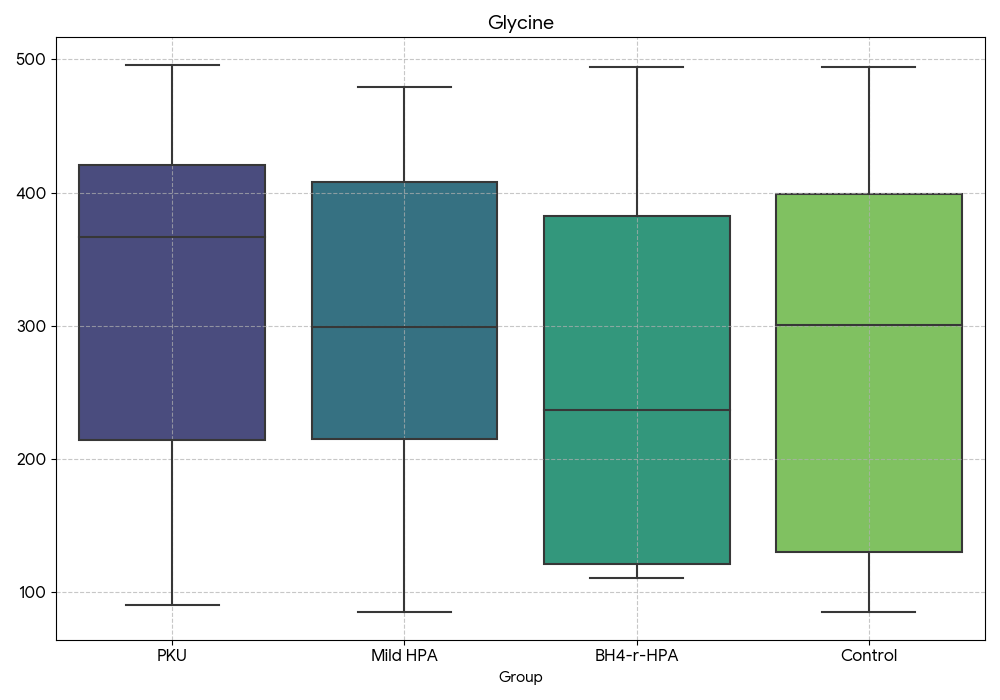


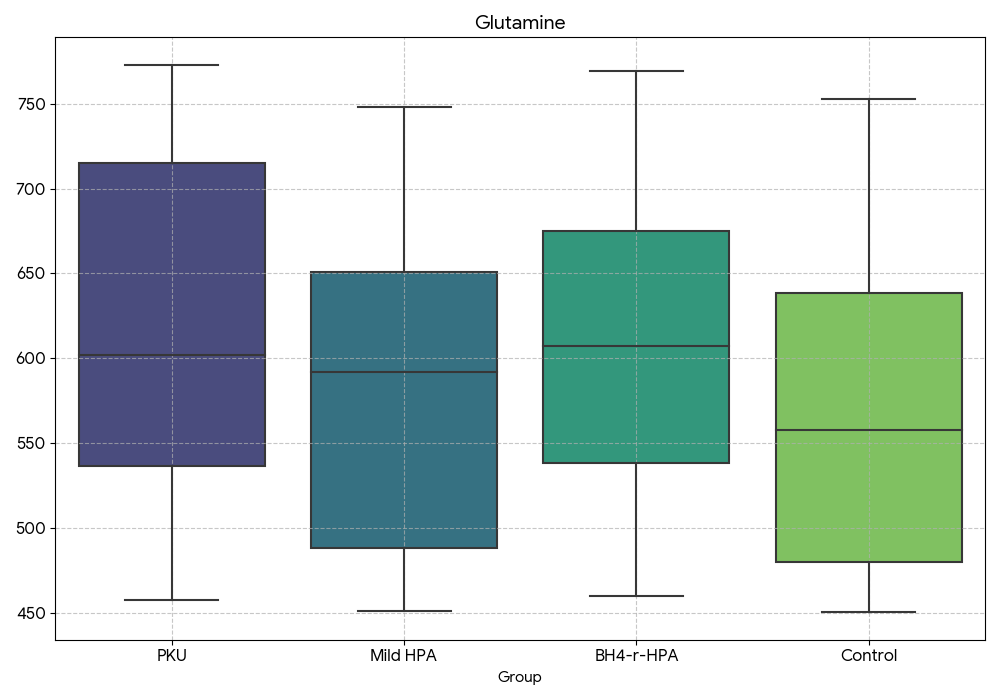


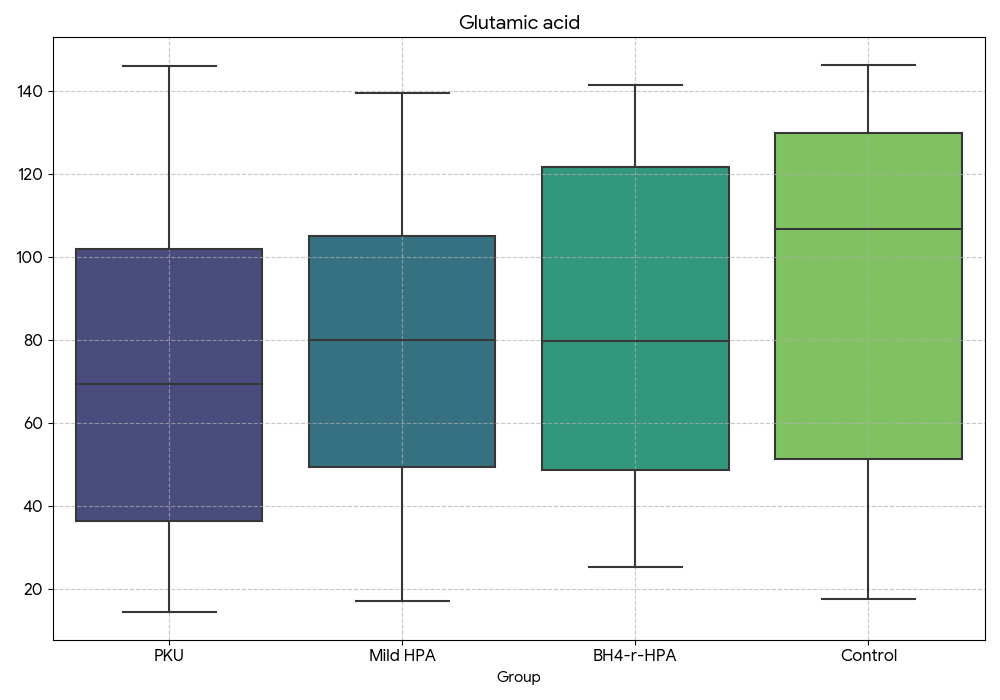


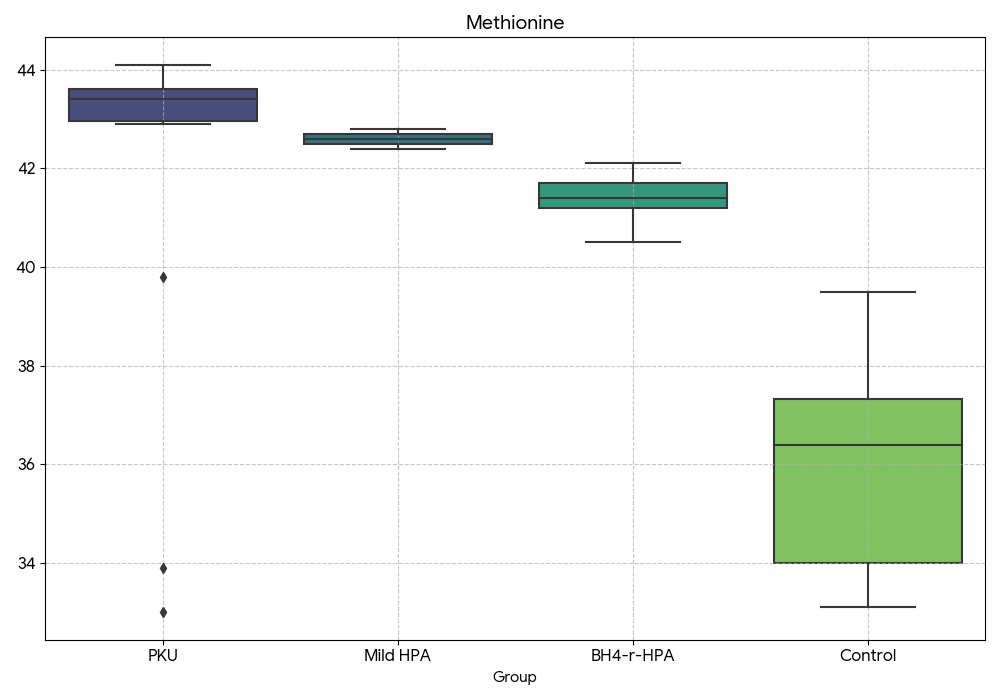


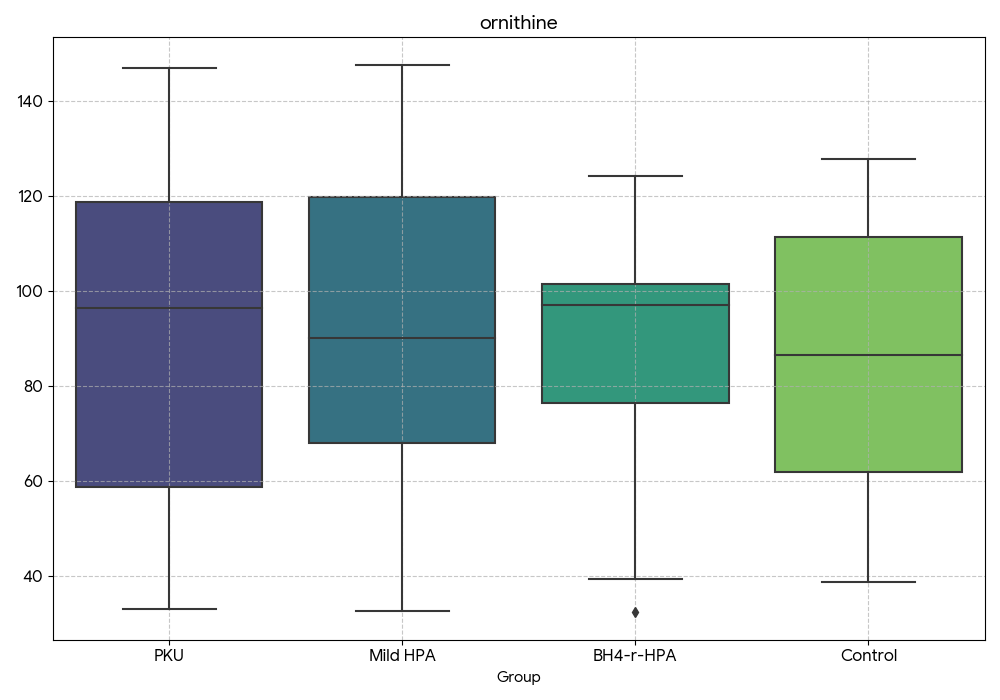


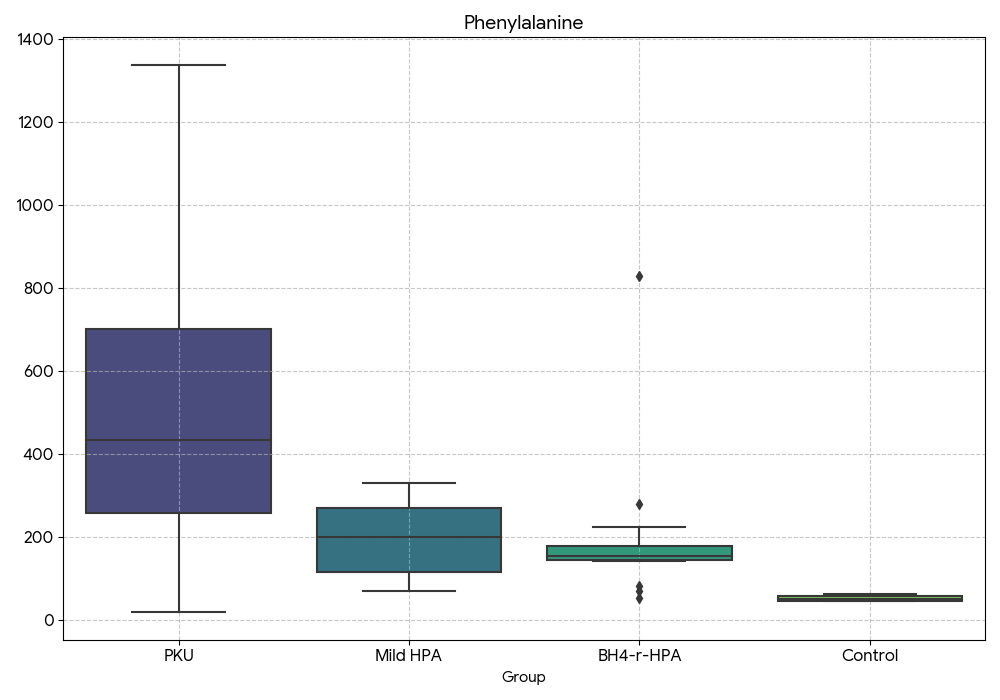


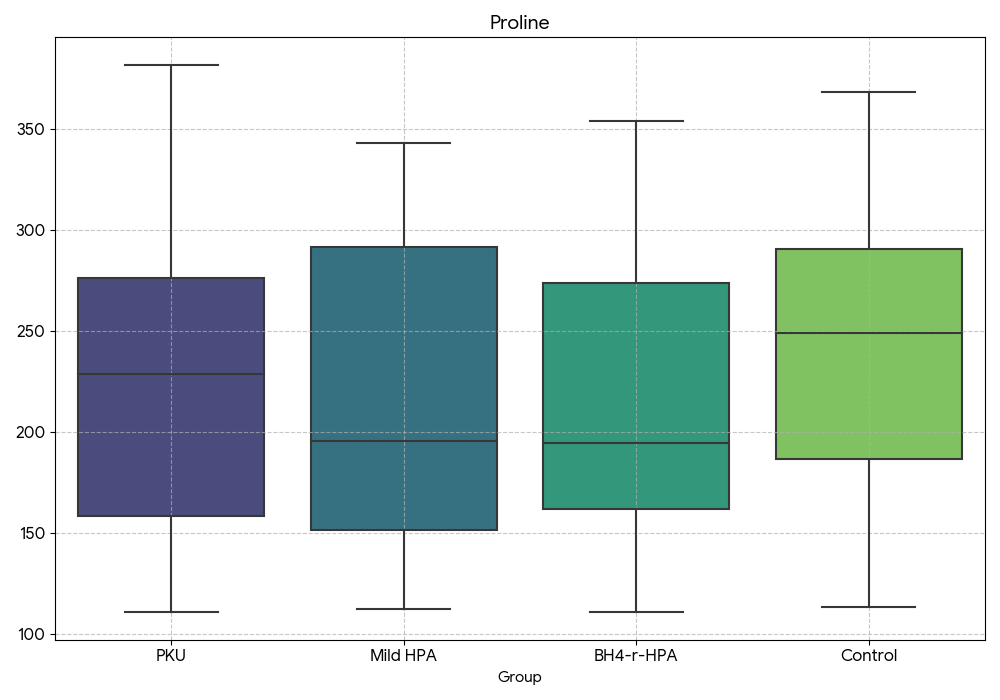


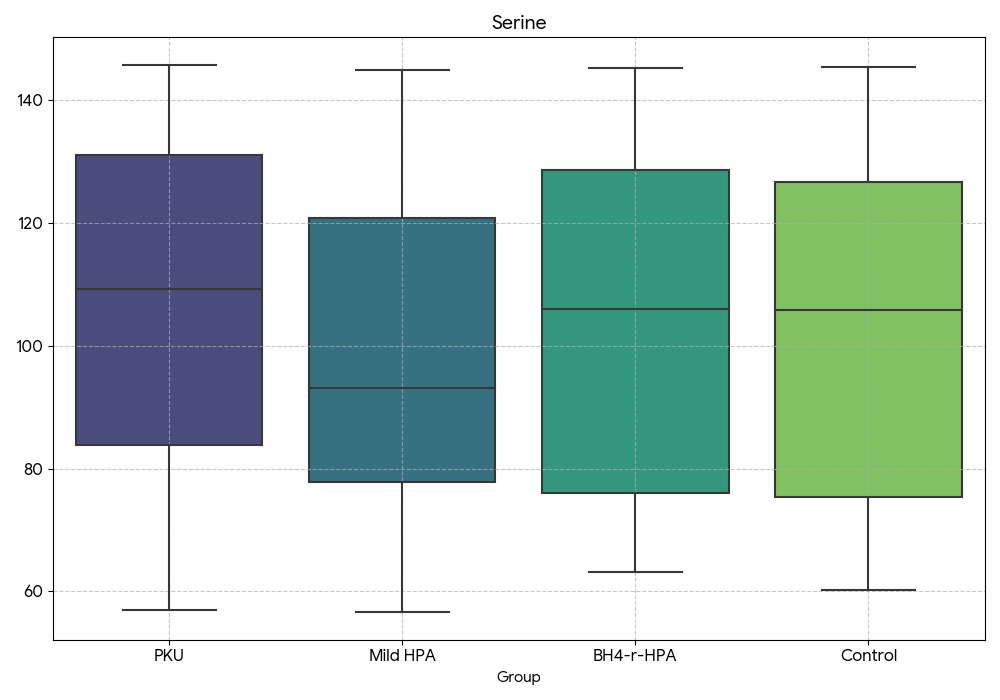


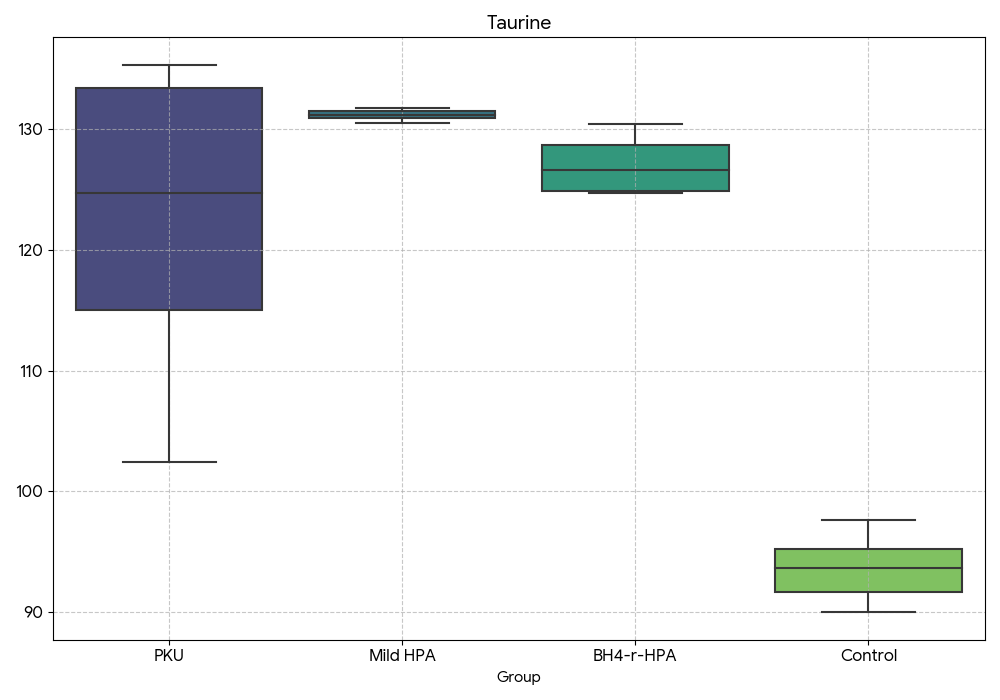


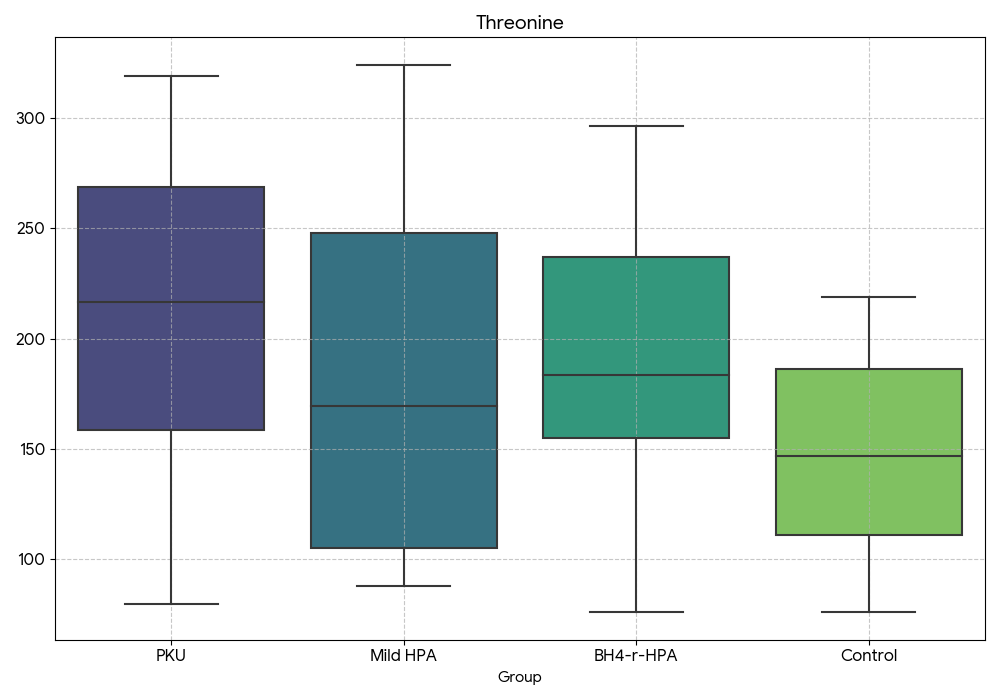


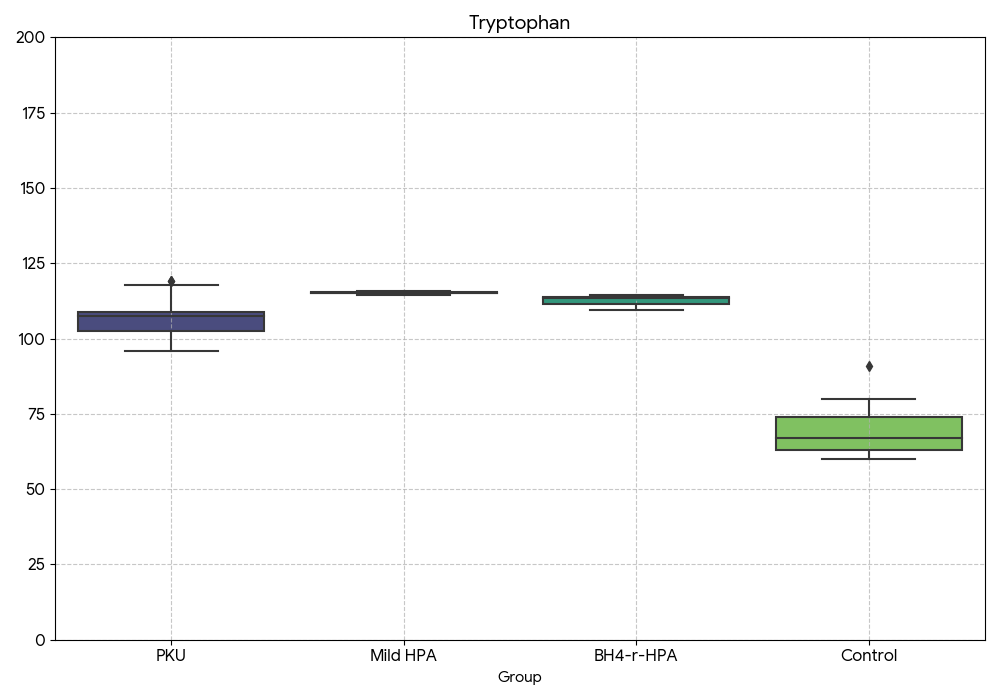


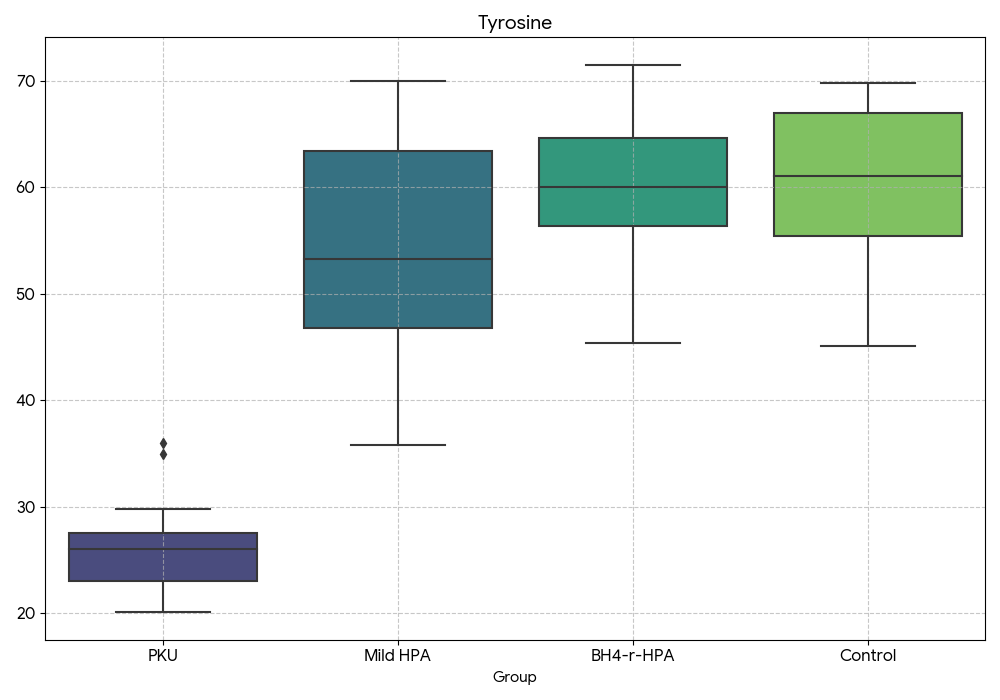


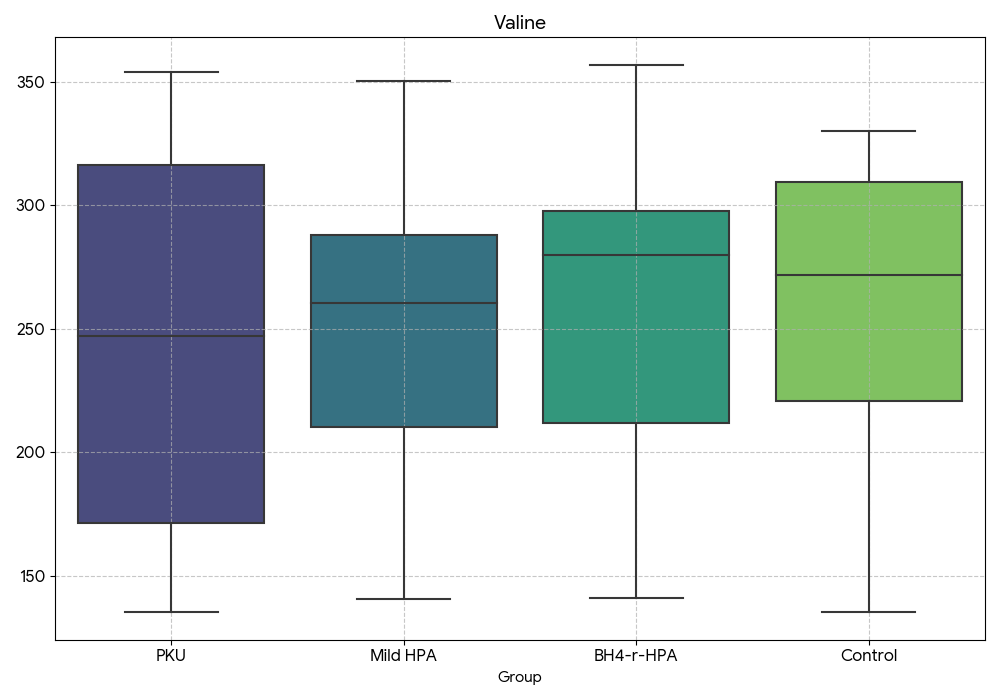


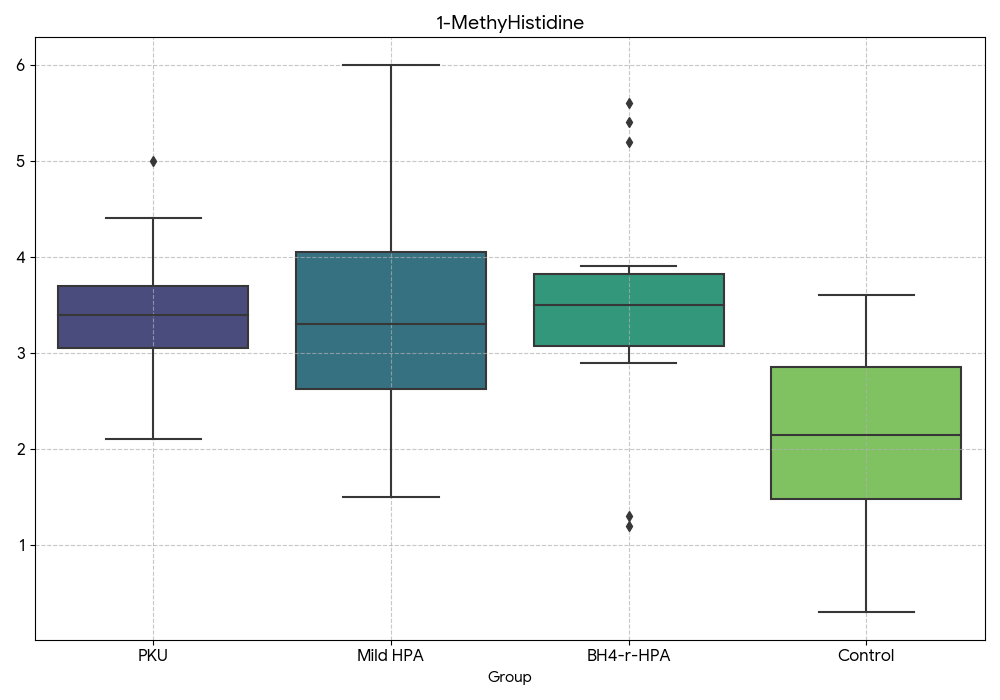


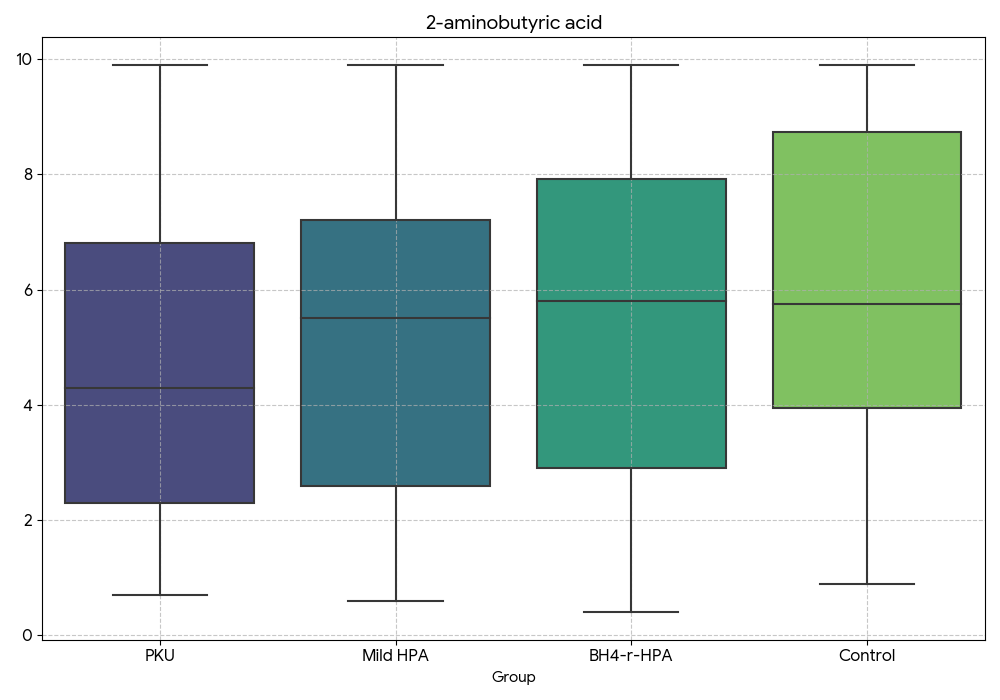


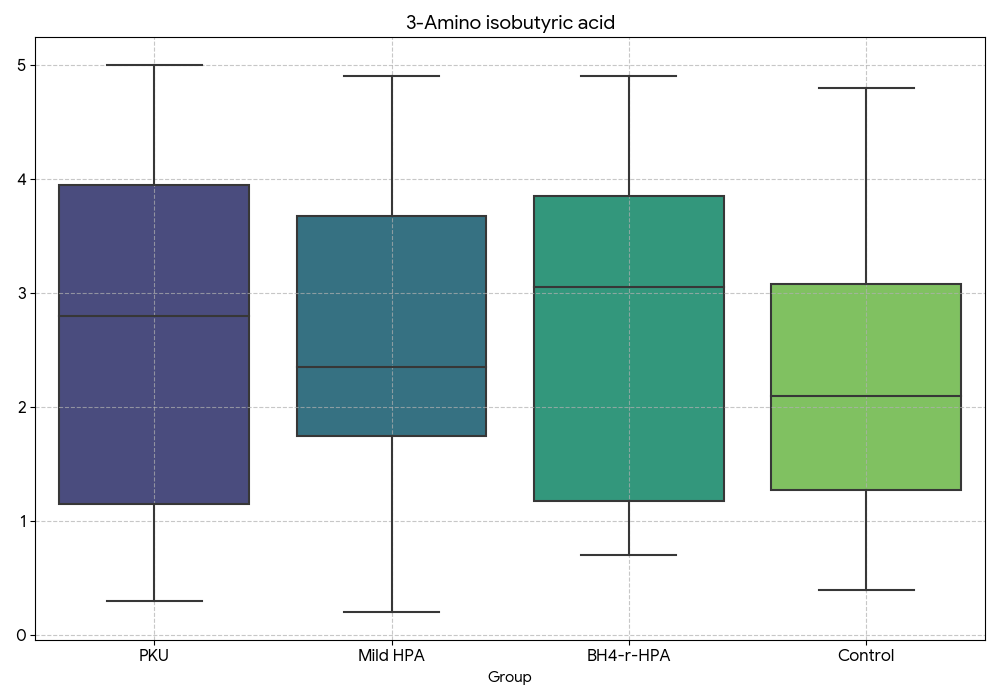


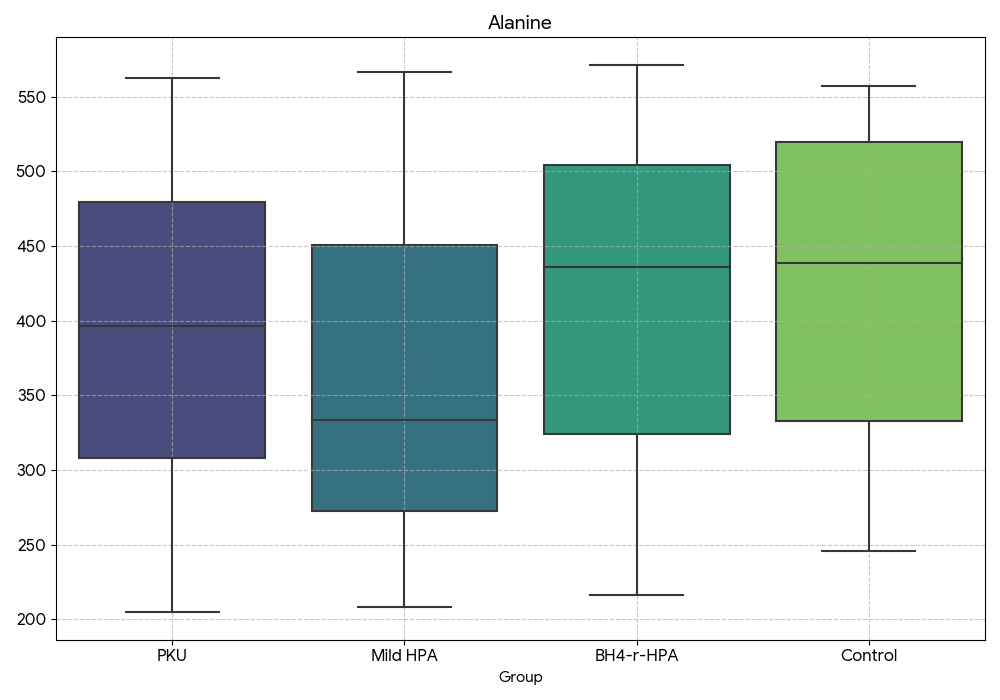


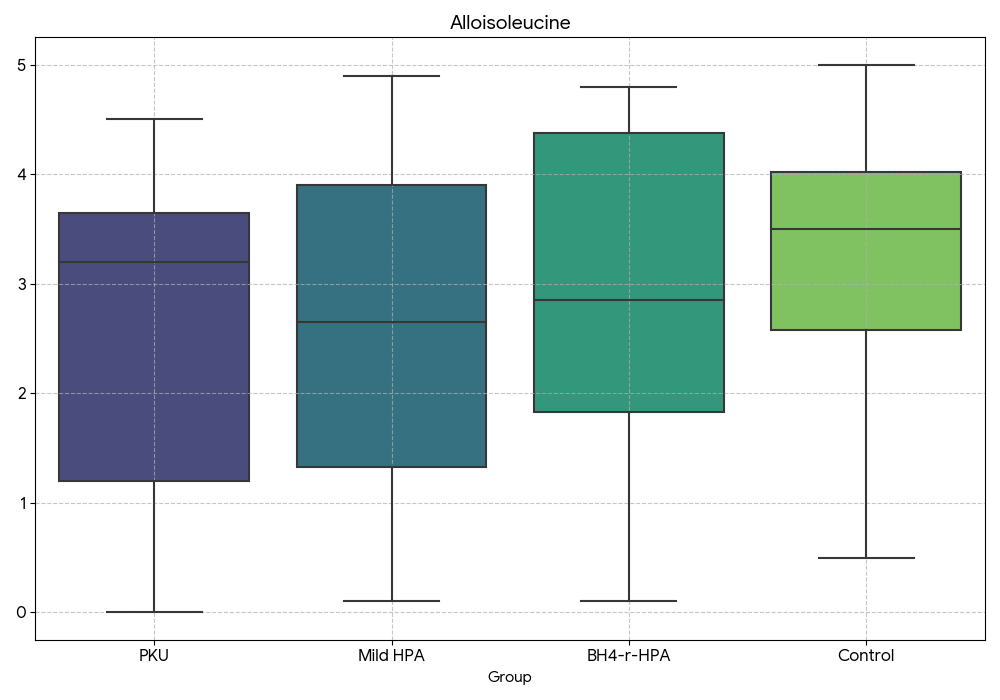


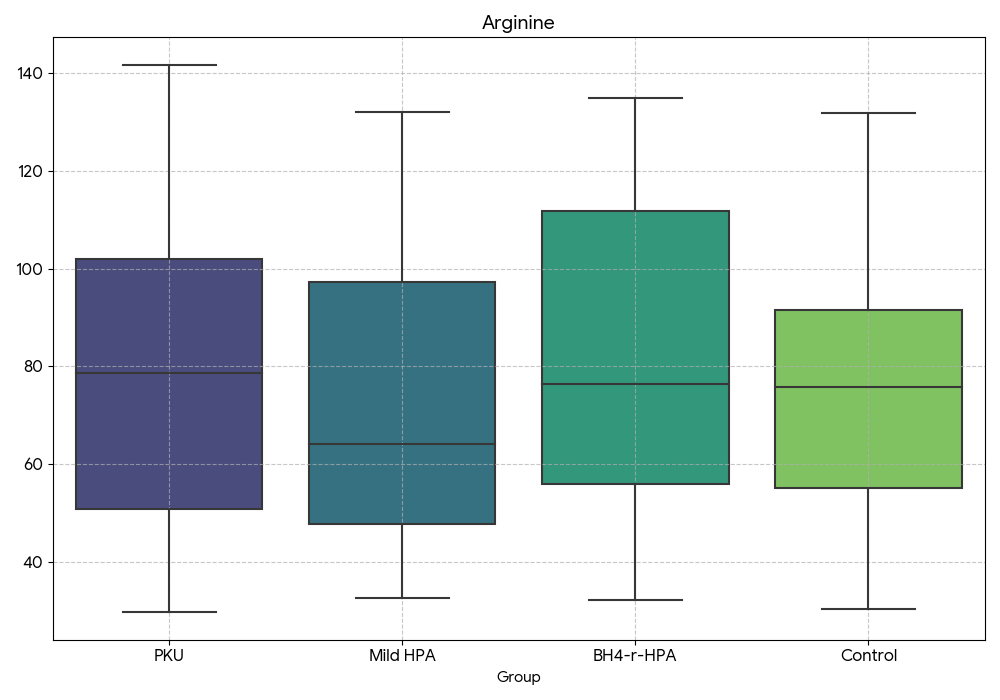


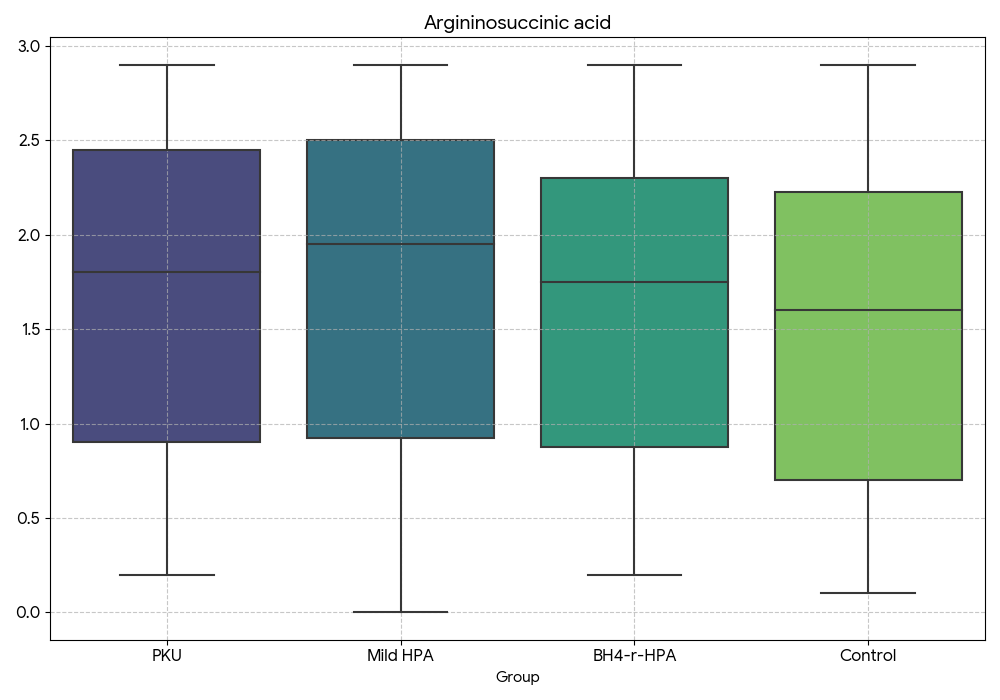


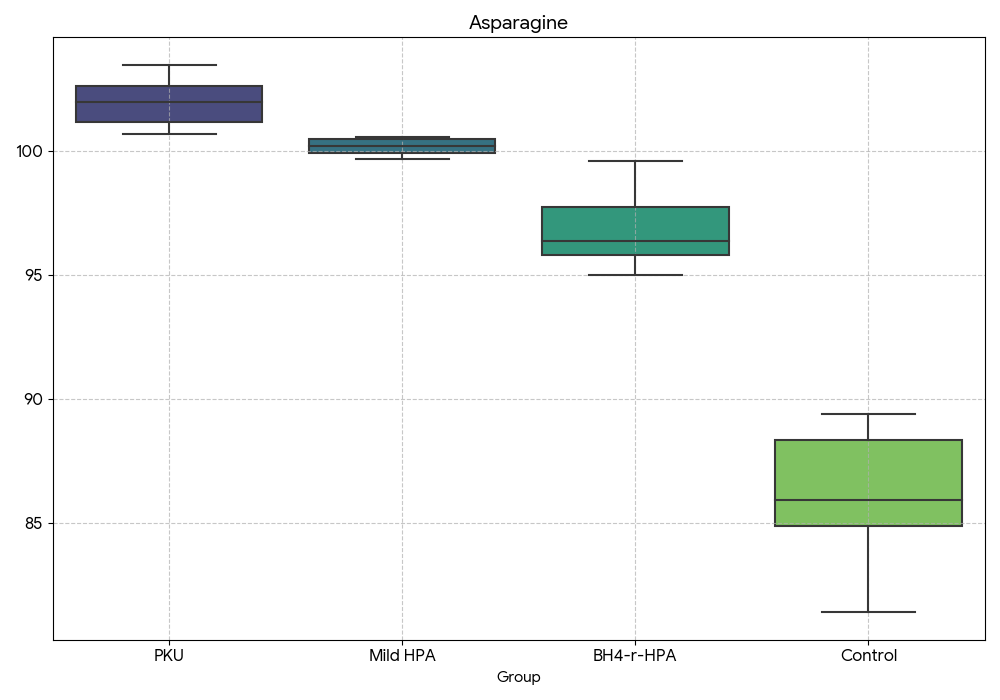


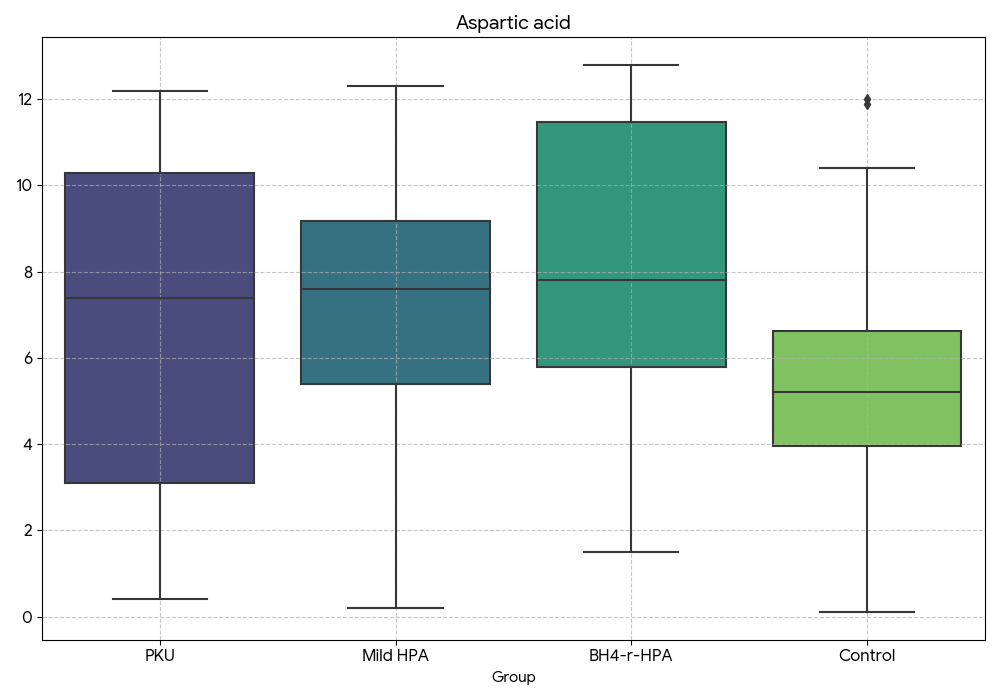


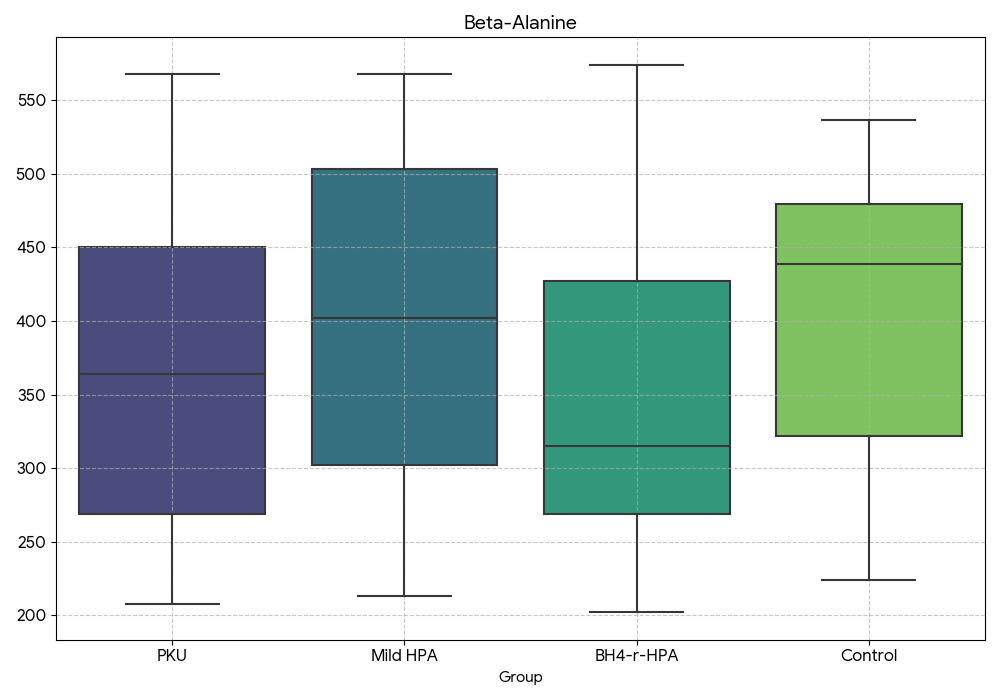


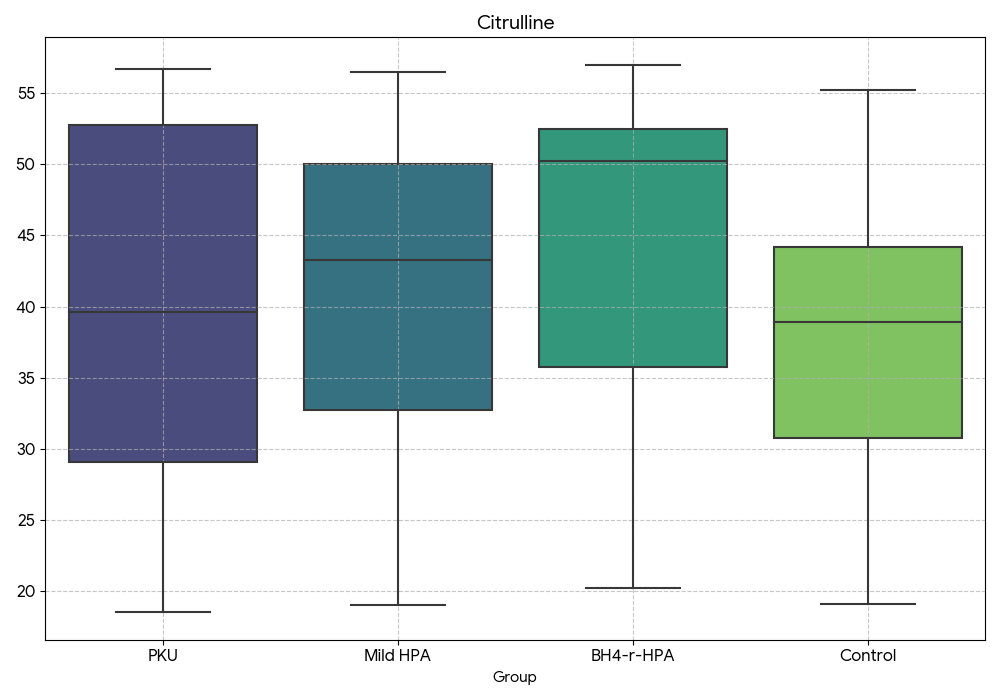


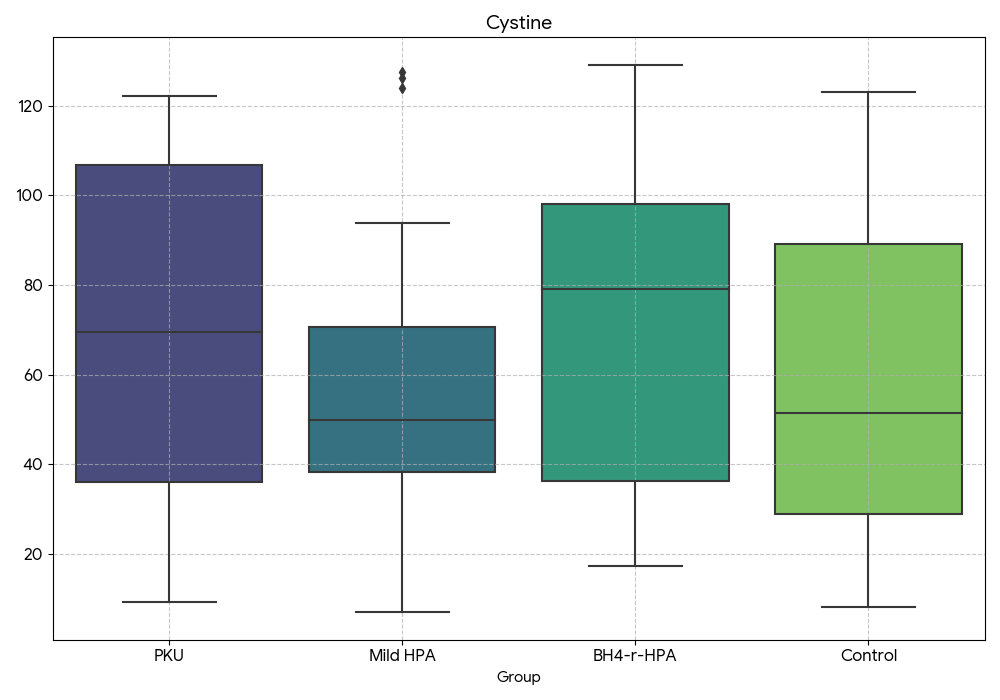


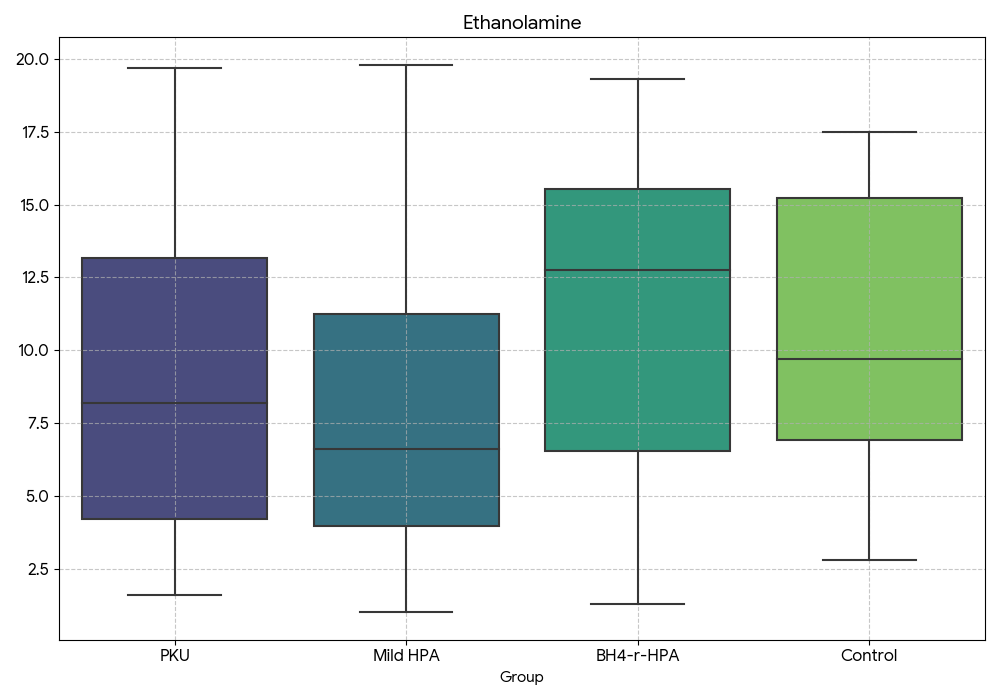


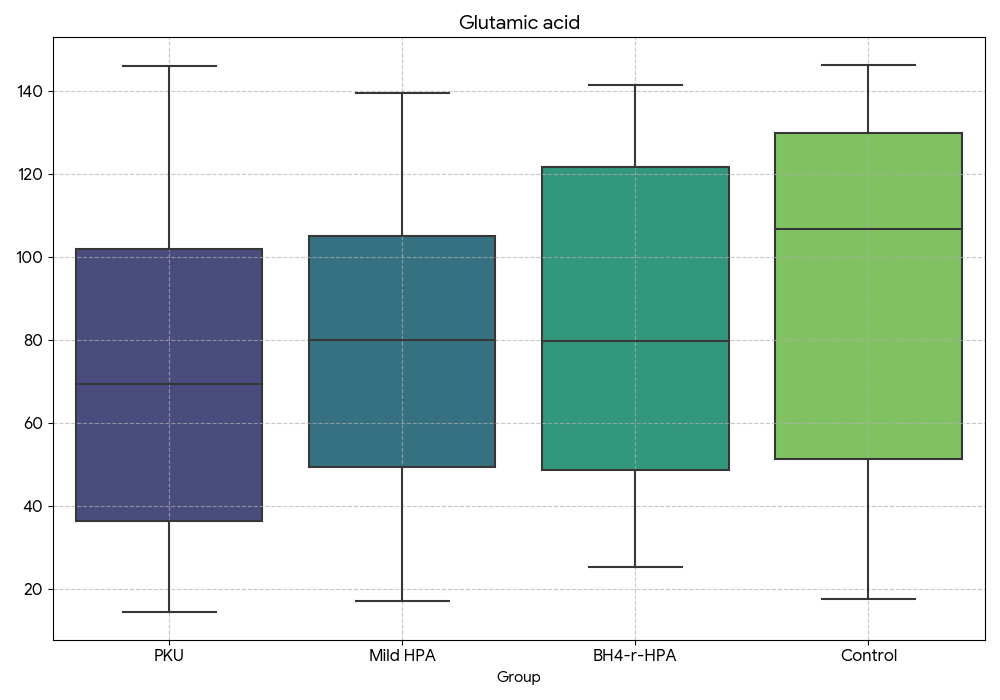


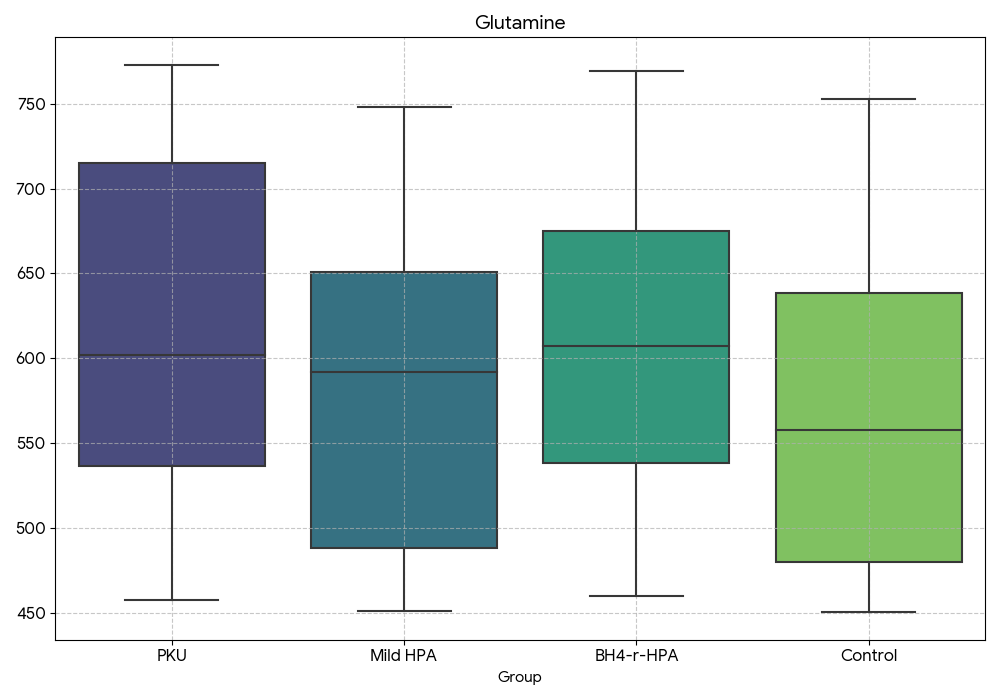


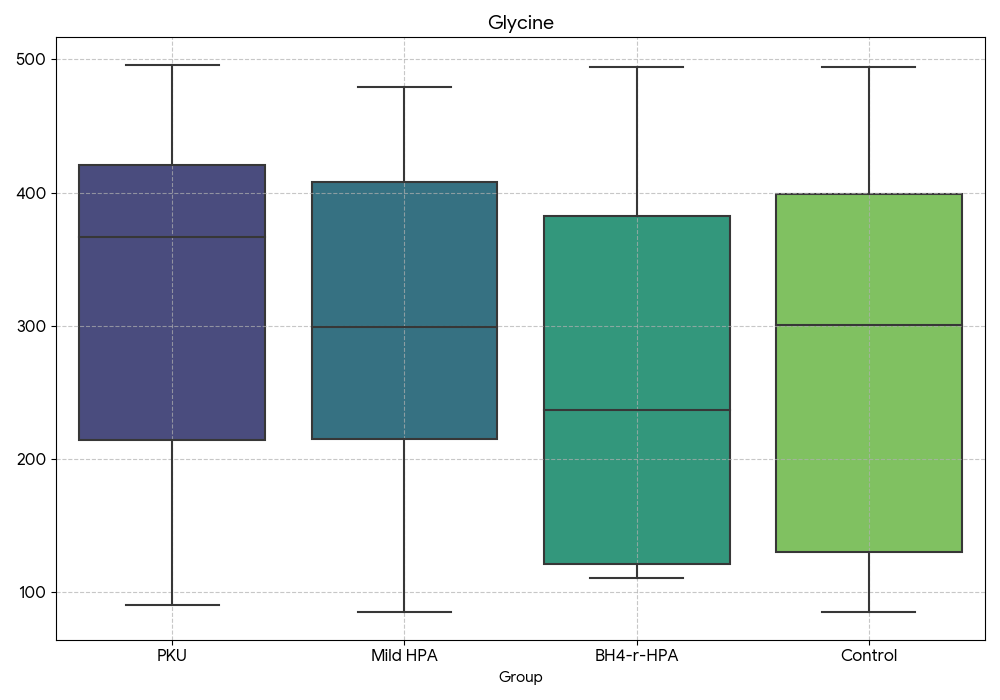


**Figure S2.** **PCA analysis (left) and OPLS-DA analysis modelling parameters(right) for A) PKU B) HPA C) BH4-r-HPA**

**
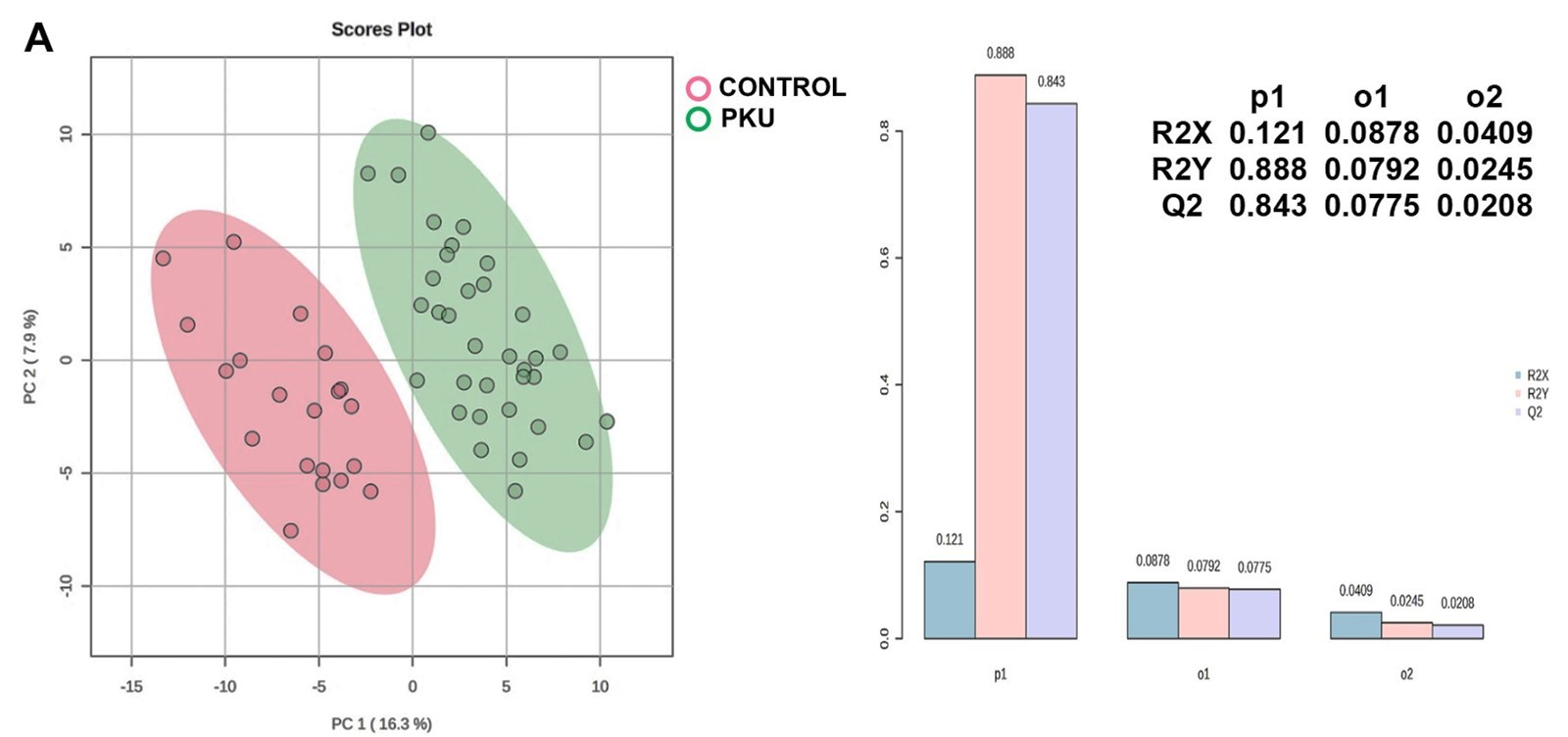
**

**
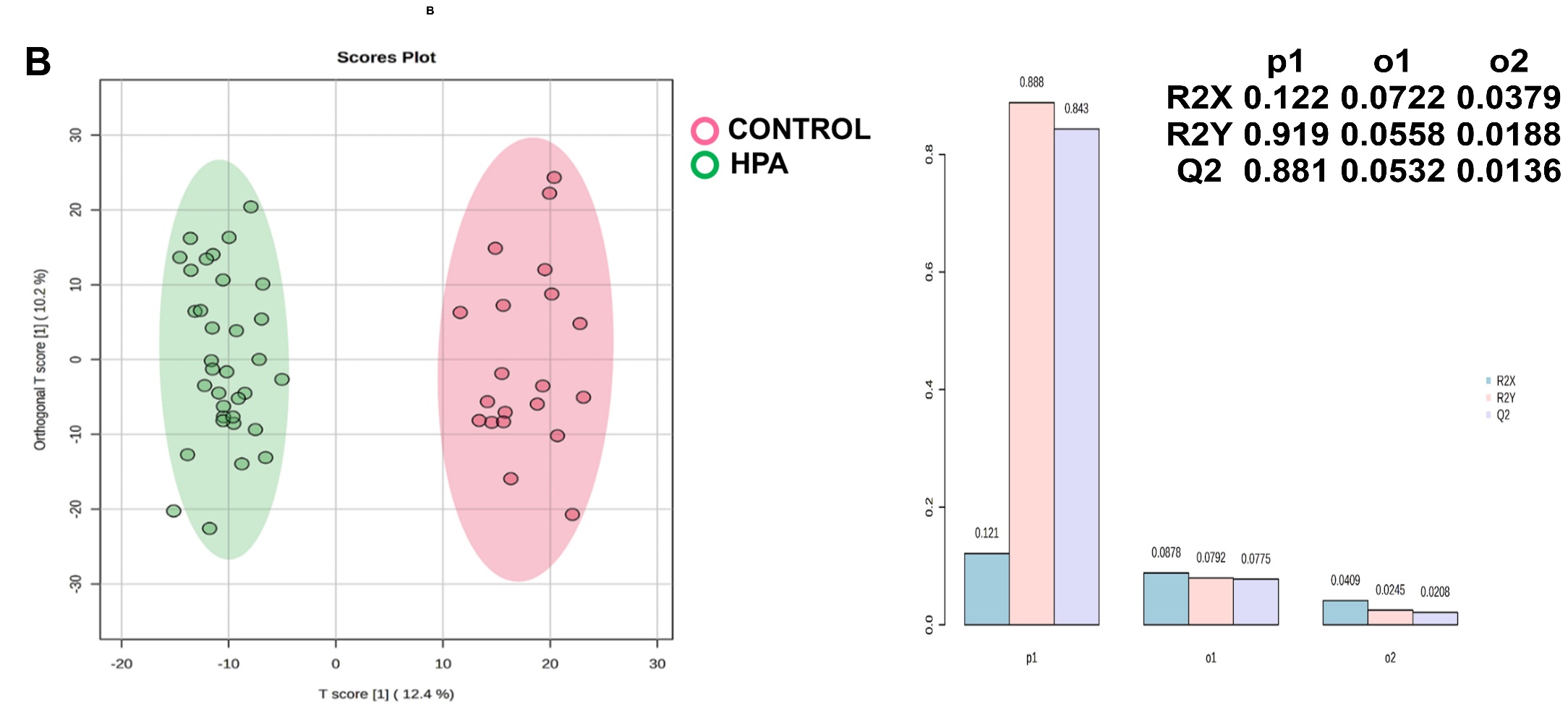
**

**
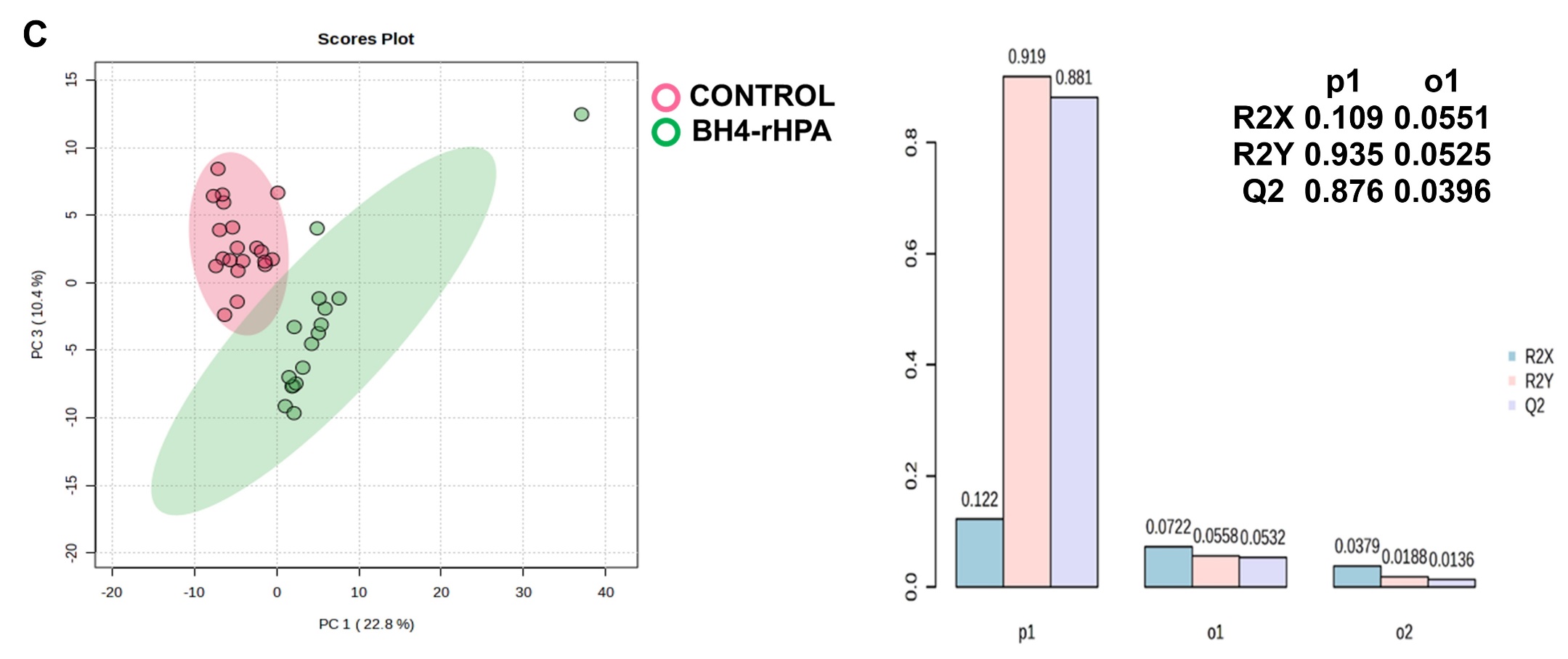
**

**Figure S3. Volcano Plot for the differential metabolites of PKU against controls**


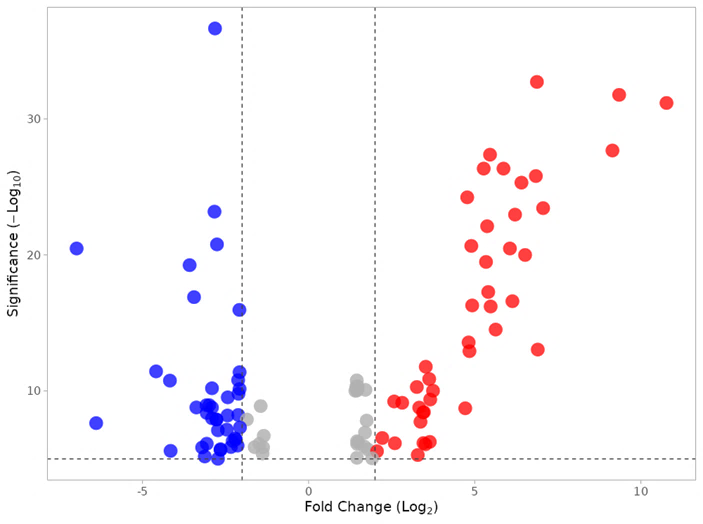


**Figure S4. Volcano Plot for the differential metabolites of HFA against controls**


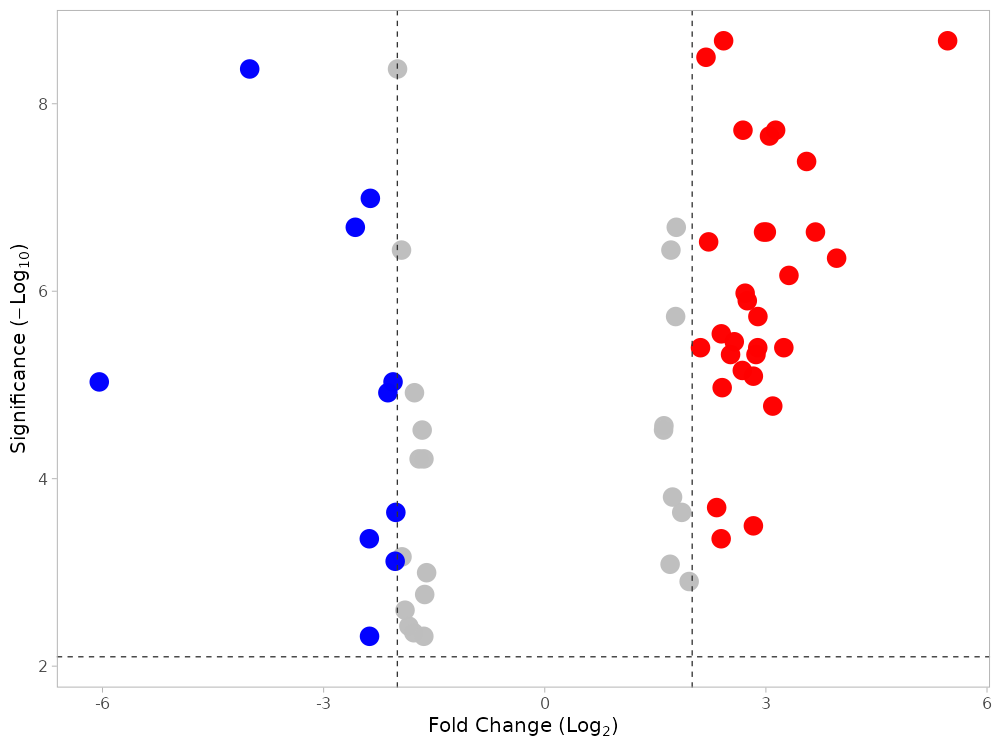


**Figure S5. Volcano Plot for the differential metabolites of BH4-r-HFA against controls**


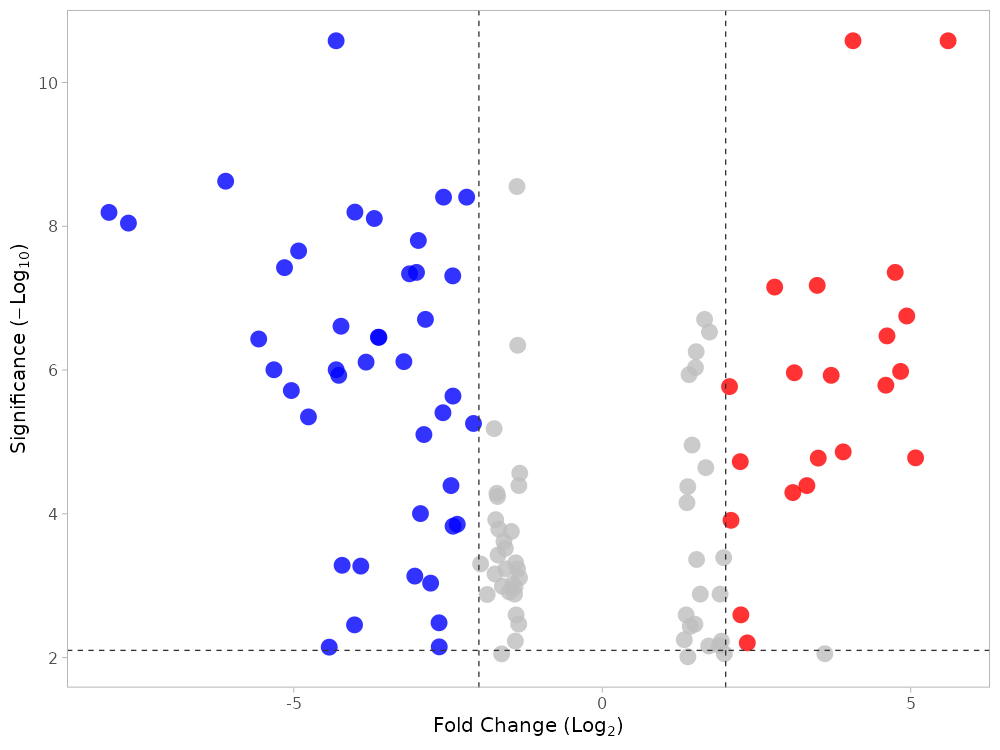


**Figure S6.** **OPLS-DA analysis modelling parameters for A) CKPKU versus MILD HPA B) CKPKU versus BH4R-MILD HPA C) BH4-r-MILD HPA**

| 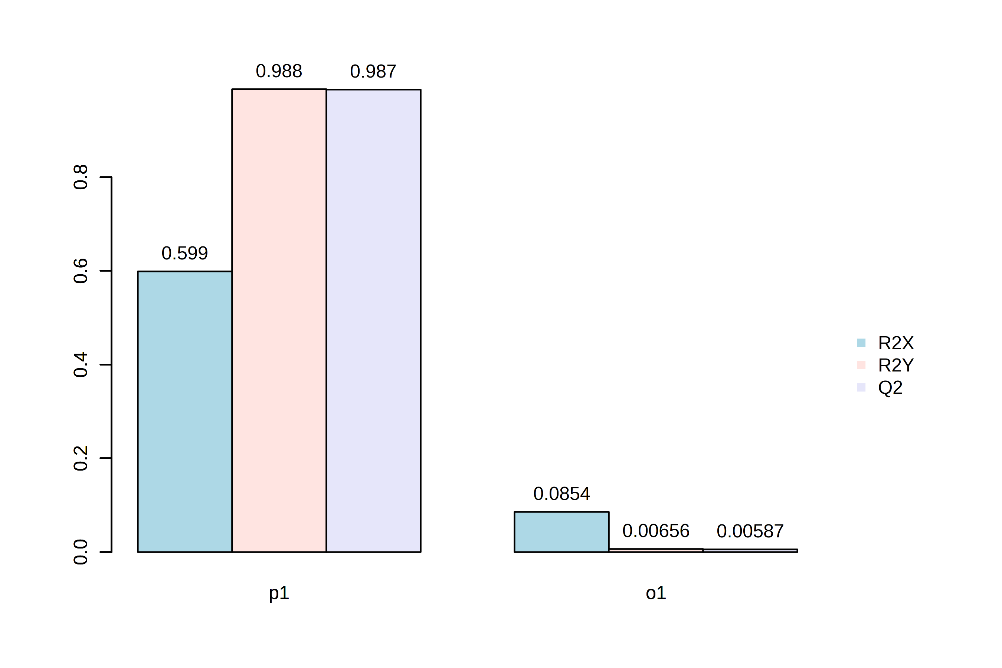  p1 o1  R2X 0.599 0.0854  R2Y 0.988 0.00656  Q2 0.987 0.00587  **A** |
| --- |

| 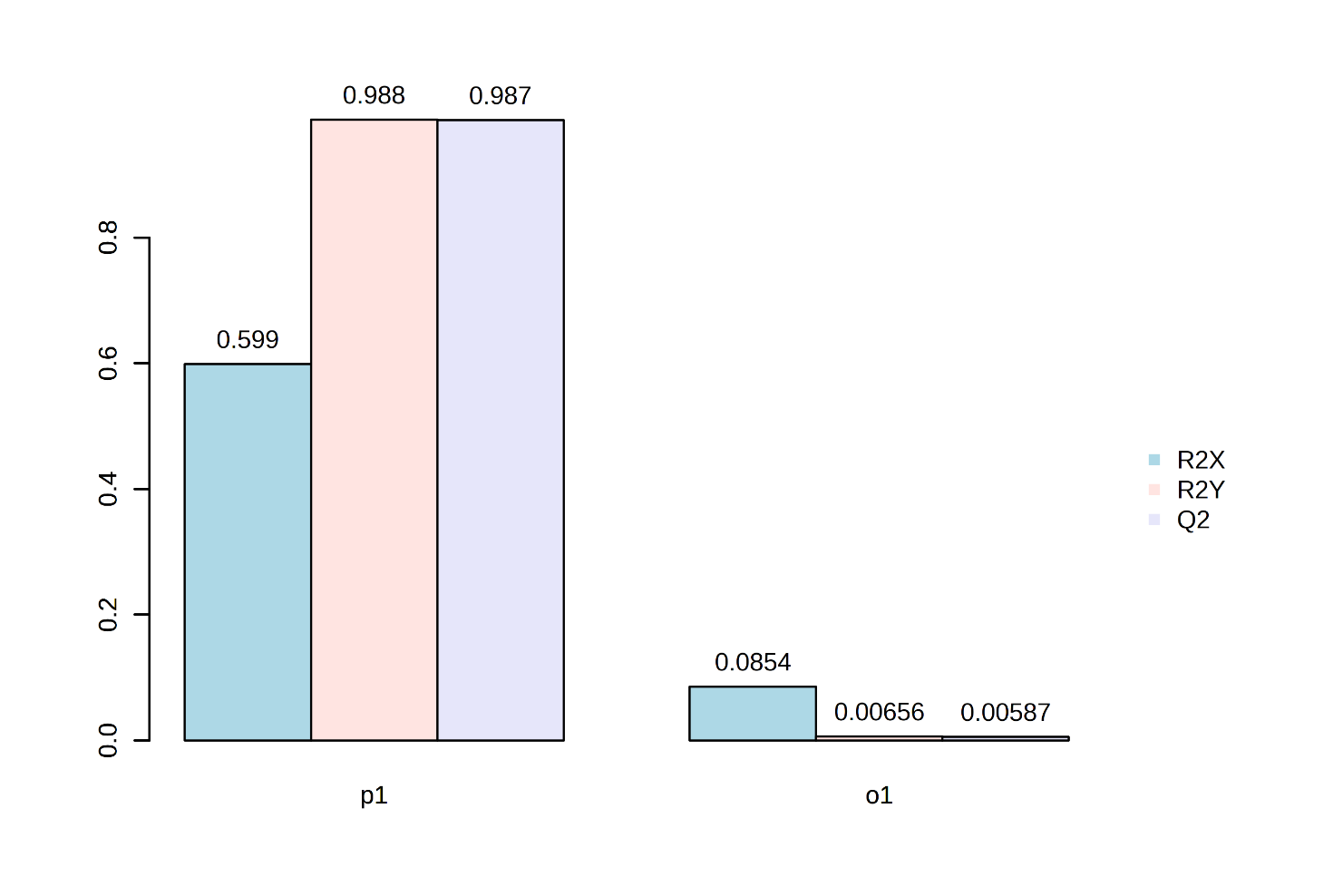   \|  \| p1 \| o1 \| \| --- \| --- \| --- \| \| R2X \| 0.609 \| 0.0429 \| \| R2Y \| 0.997 \| 0.00188 \| \| Q2 \| 0.996 \| 0.000692 \|   **B** |
| --- | --- | --- | --- | --- | --- | --- | --- | --- | --- | --- | --- | --- |

| 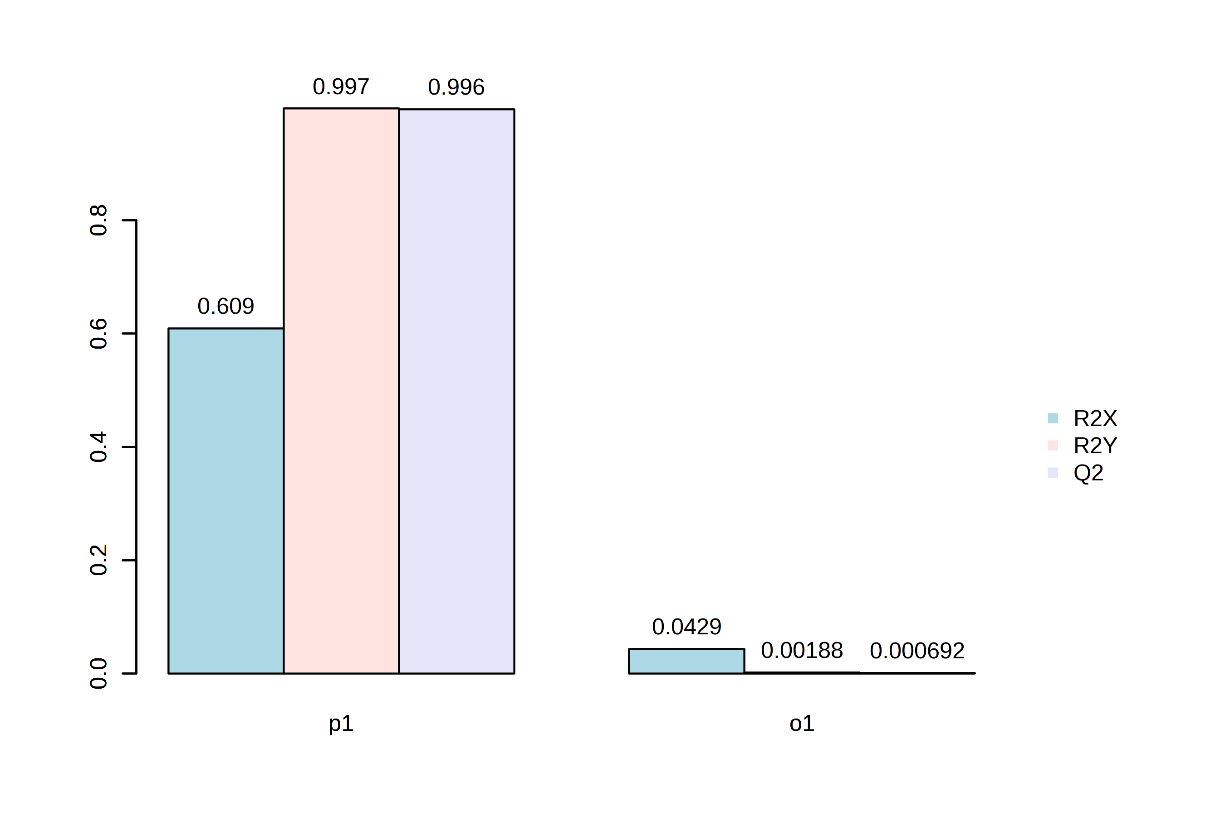   \|  \| p1 \| o1 \| \| --- \| --- \| --- \| \| R2X \| 0.609 \| 0.0429 \| \| R2Y \| 0.997 \| 0.00188 \| \| Q2 \| 0.996 \| 0.000692 \|   **C** |
| --- | --- | --- | --- | --- | --- | --- | --- | --- | --- | --- | --- | --- |


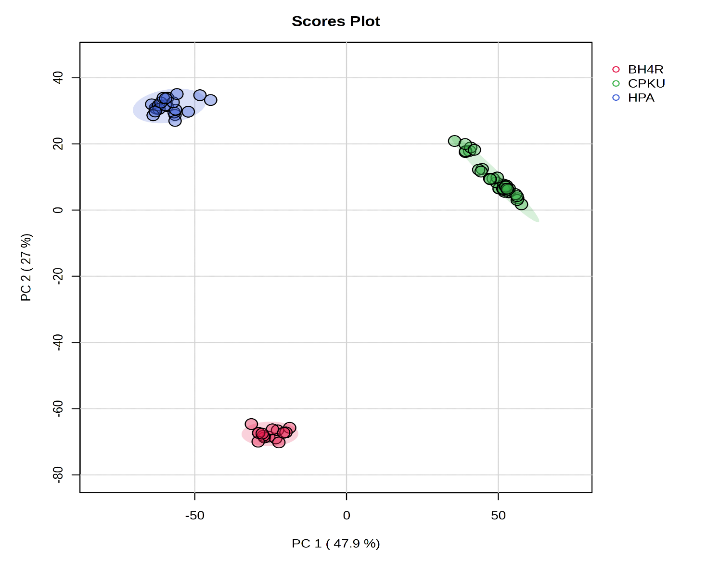


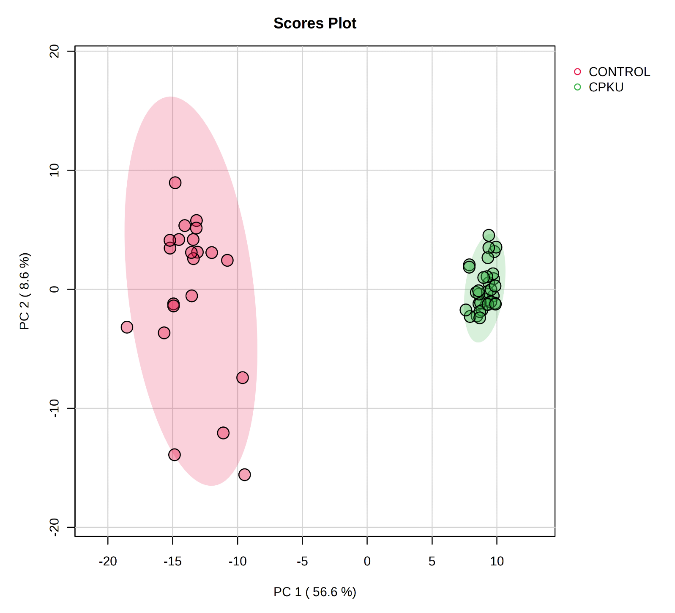

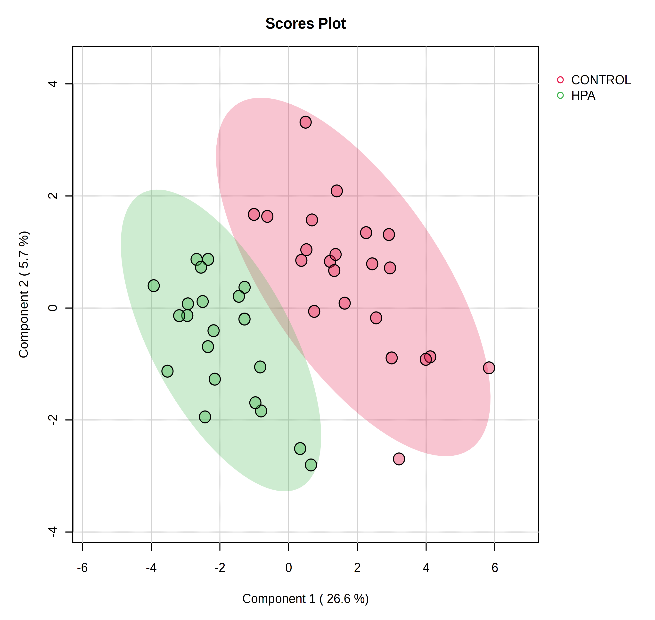

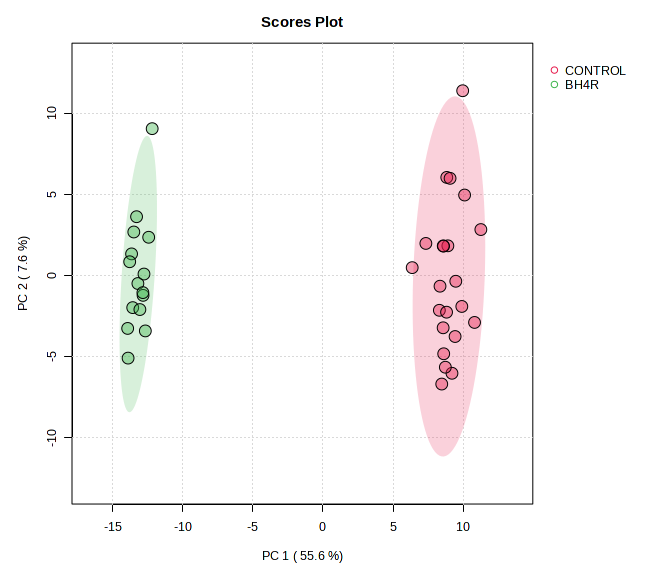
**Figure S7.** **PCA Analysis of 3 group comparison and comparison of each clinical phenotype with respect to each other**

**
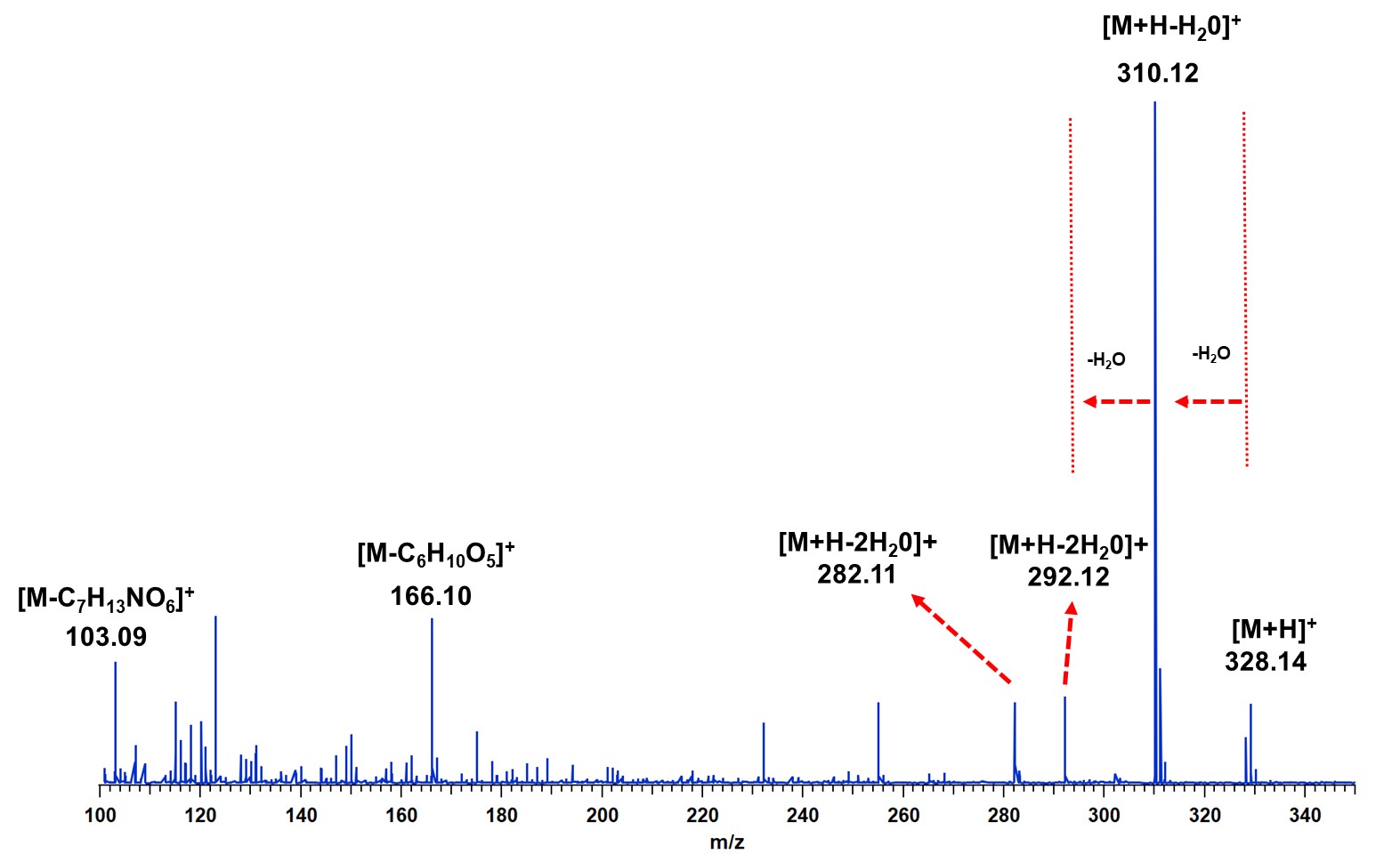
**

**Figure S8. LC-MS/MS fragmentation spectrum of the mass selected m/z=328.14 peak at CE=25 V**

**Figure S9. ROC plots and box-plots of the top 15 metabolites with high VIP scores for CKPKU samples**

**N-(1-Deoxy-1-fructosyl) phenylalanine**

| 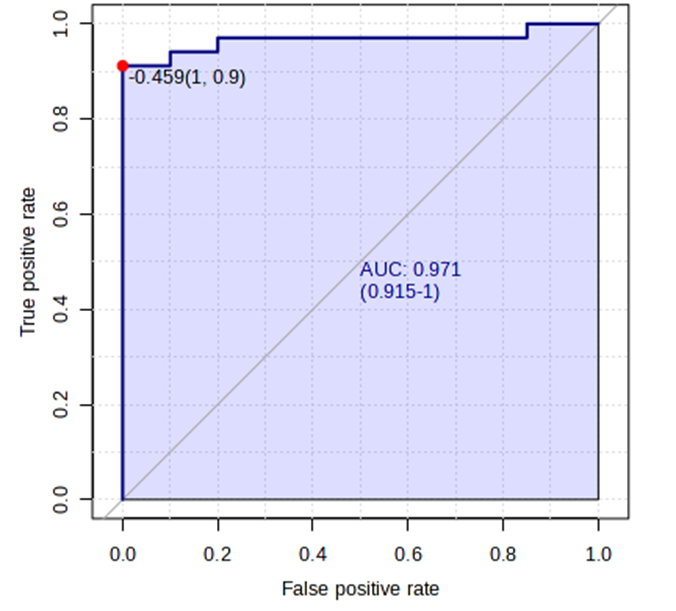 | 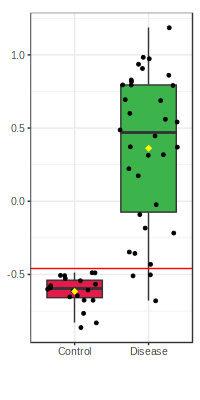 |
| --- | --- |

**Phenylalanine**

| 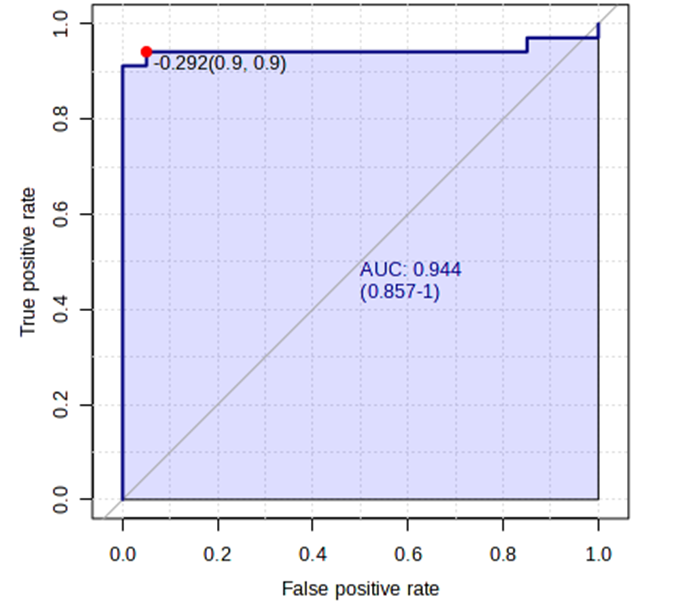 | 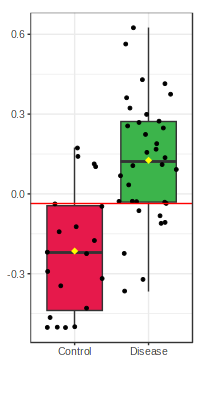 |
| --- | --- |

**Phenyllactic**

| 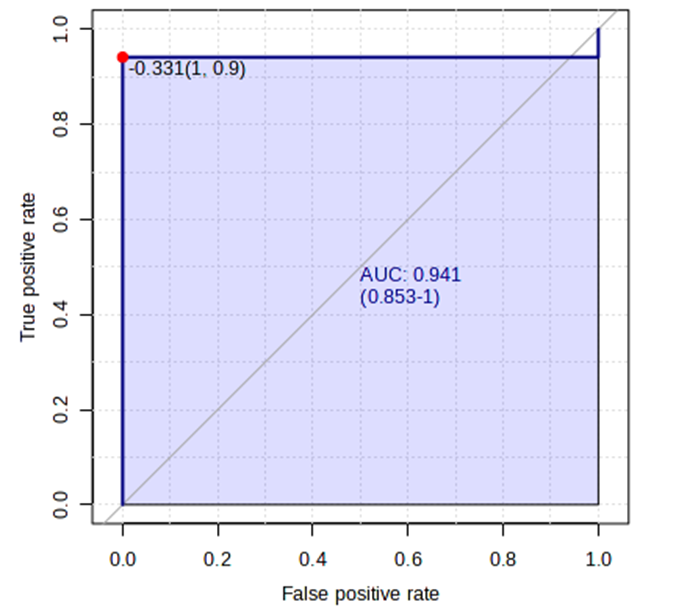 | 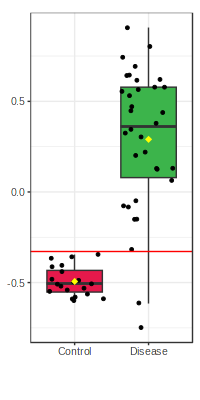 |
| --- | --- |

**Glycyl-Histidine**

| 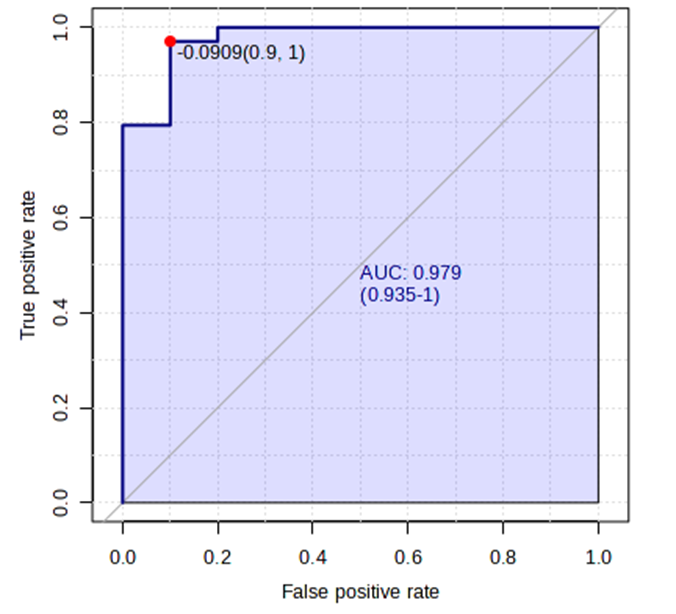 | 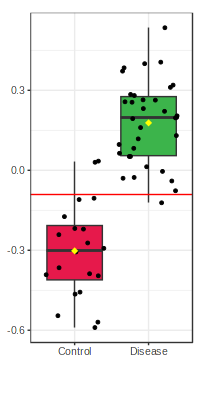 |
| --- | --- |

**Glutamylproline**

| 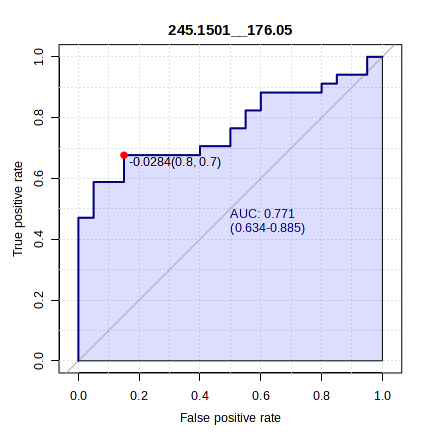 | 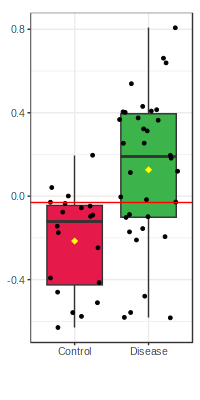 |
| --- | --- |

**Sphingosine-1-Phosphate**

| 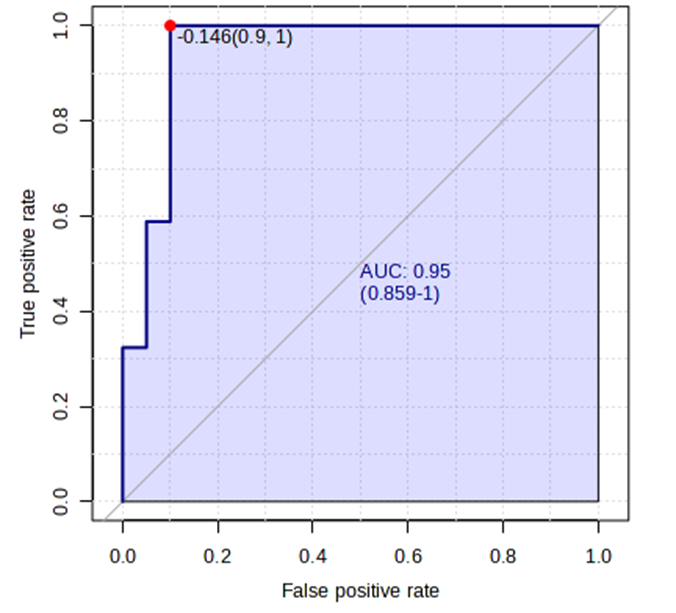 | 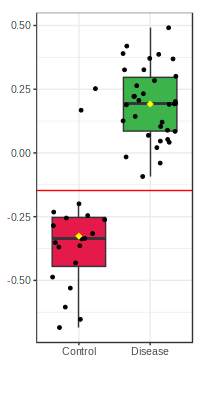 |
| --- | --- |

**Cinnamic acid**

| 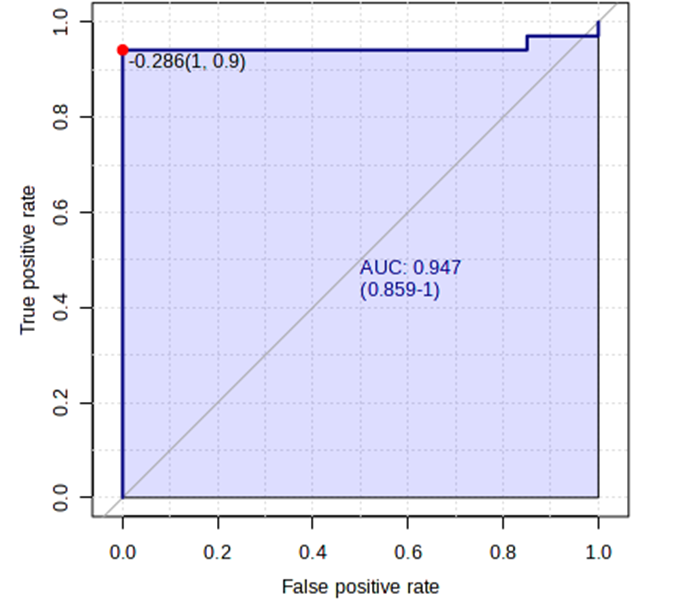 | 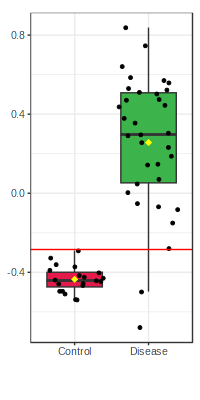 |
| --- | --- |

**L-Kynurenine**

| 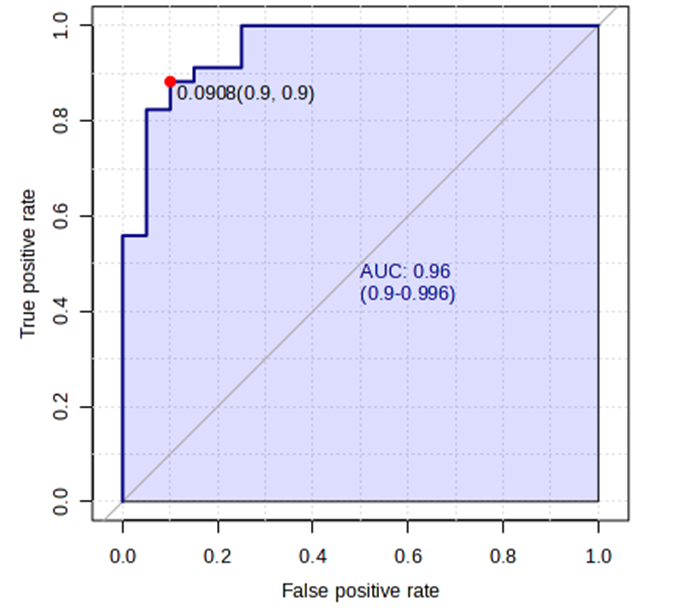 | 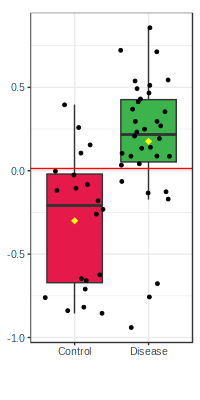 |
| --- | --- |

**Styrene**

| 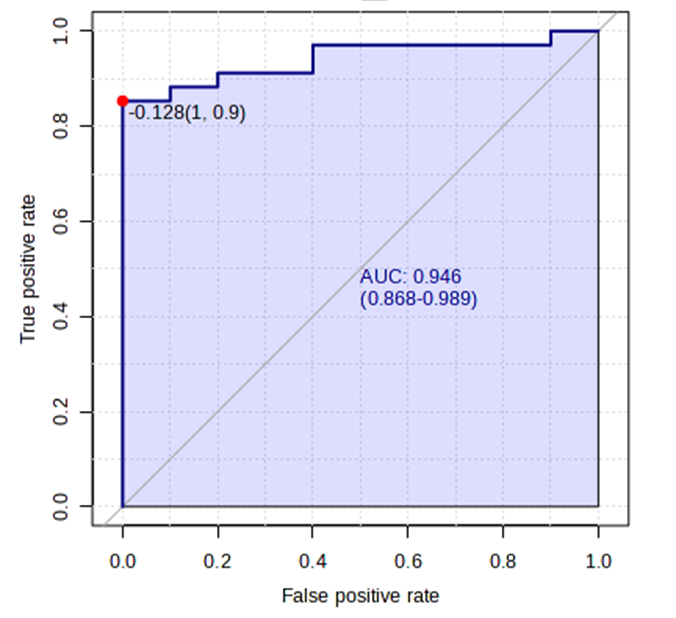 | 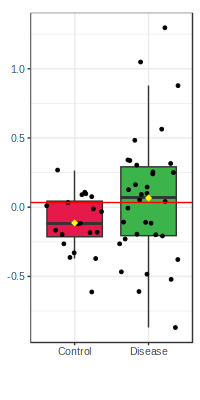 |
| --- | --- |

**Phenylpyruvic acid**

| 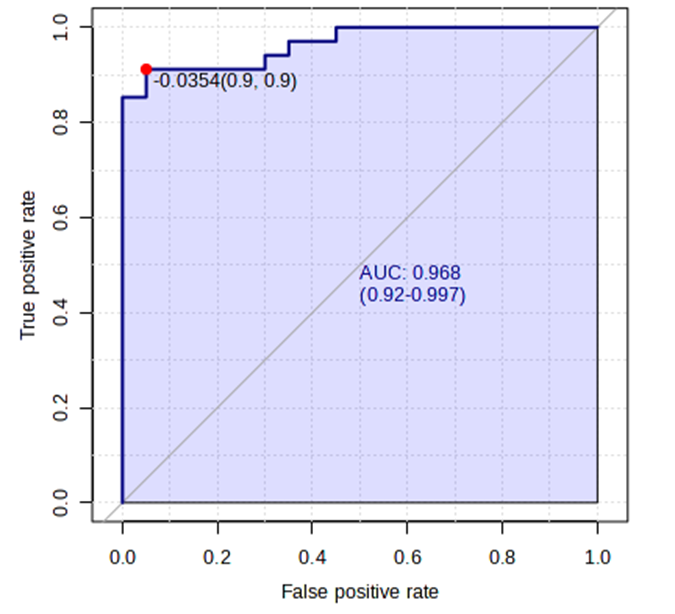 | 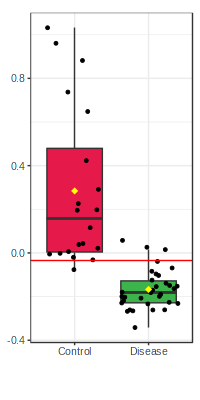 |
| --- | --- |

**Phenylalanyltryptophan**

| 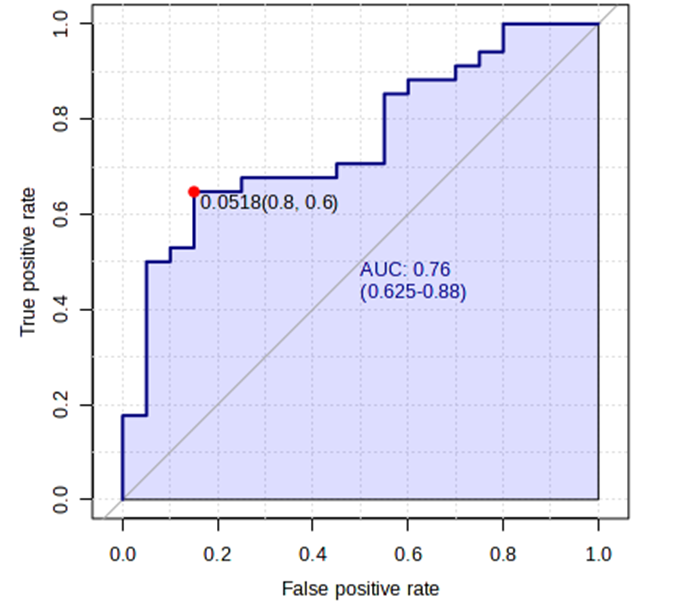 | 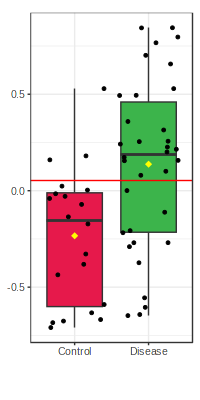  , |
| --- | --- |

**Eicosapentaenoic acid**

| 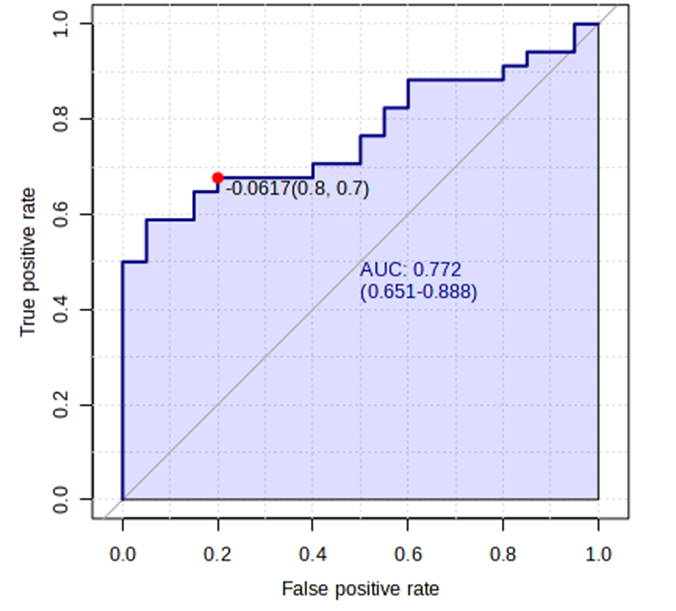 | 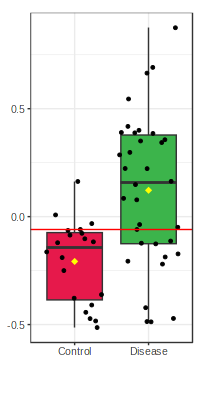 |
| --- | --- |

**Linolenyl carnitine**

| 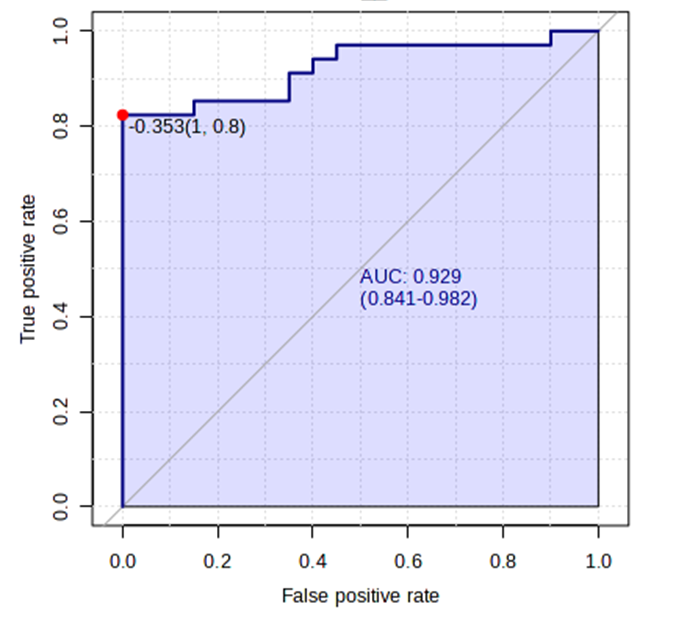 | 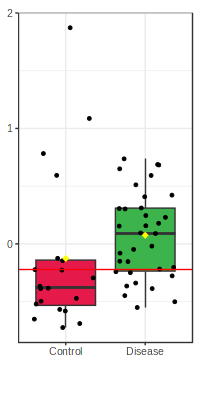 |
| --- | --- |

**Galabiosylceramide (d18:1/16:0)**

| 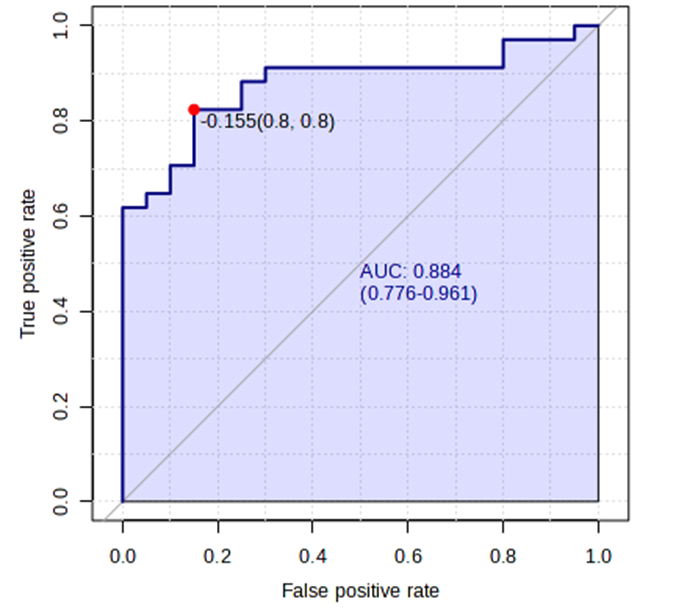 | 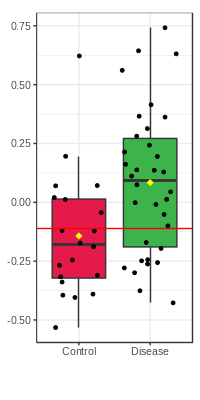 |
| --- | --- |

**Gamma-Glutamyltryosine**

| 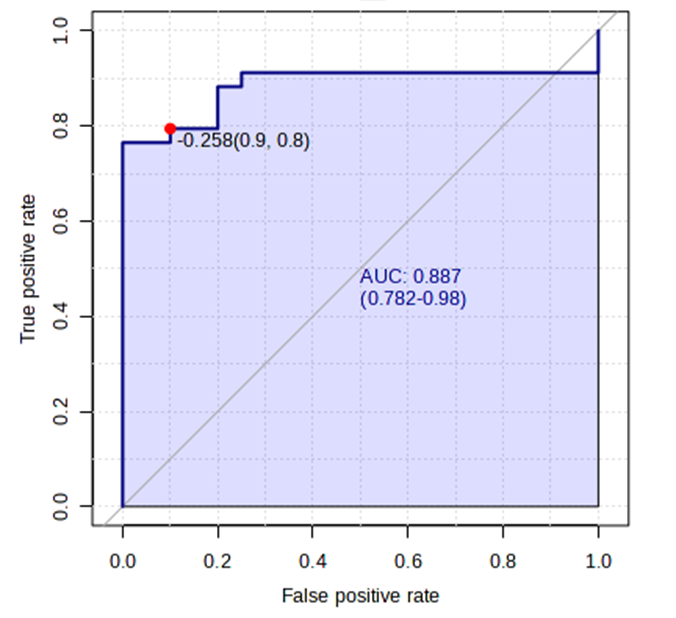 | 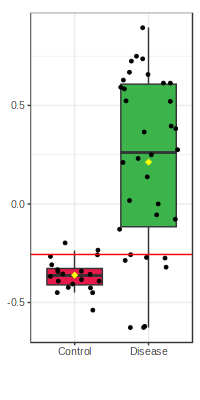 |
| --- | --- |

**Figure S10. ROC plots and box-plots of the top 15 metabolites with high VIP scores MILD HPA samples**

**N-(1-Deoxy-1-fructosyl) phenylalanine**

| 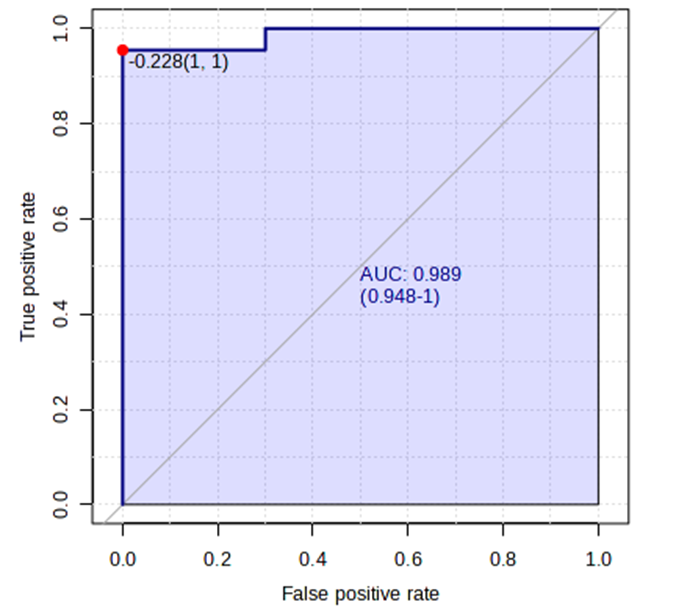 | 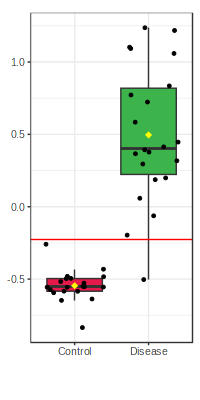 |
| --- | --- |

**Phenylalaninephenylalanine**

| 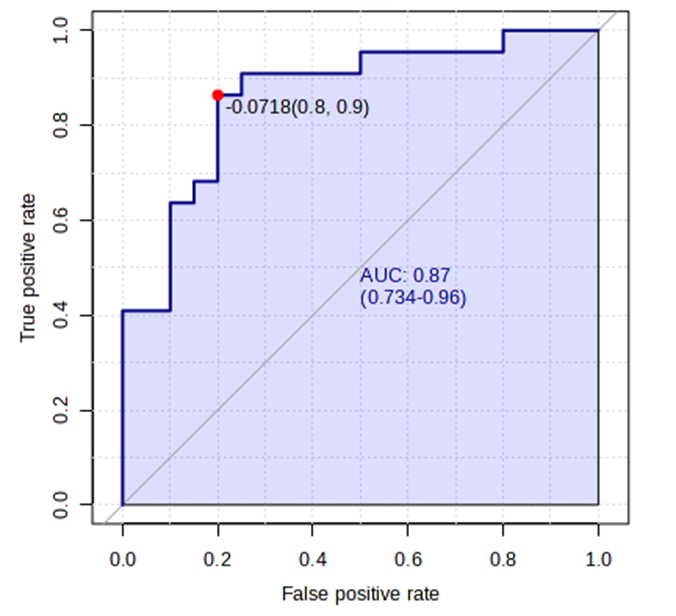 | 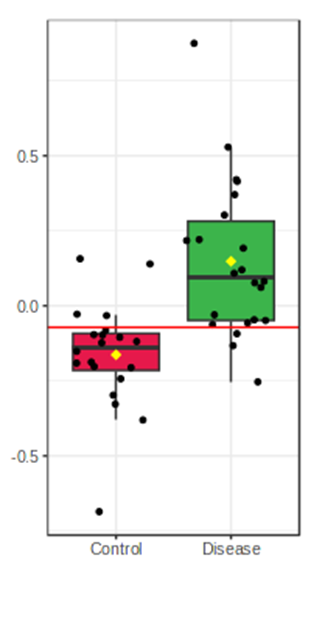 |
| --- | --- |

**Phenylalanine**

| 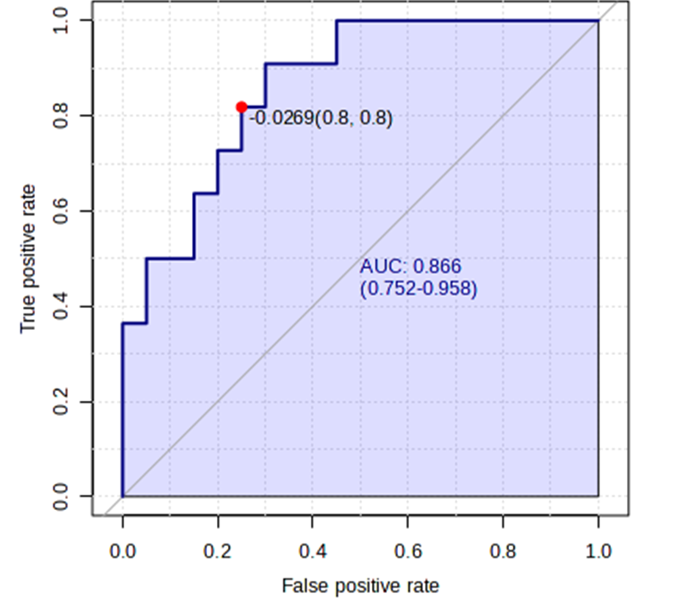 | 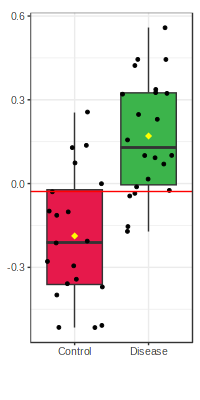 |
| --- | --- |

**Gamma-Glutamyltyrosine**

| 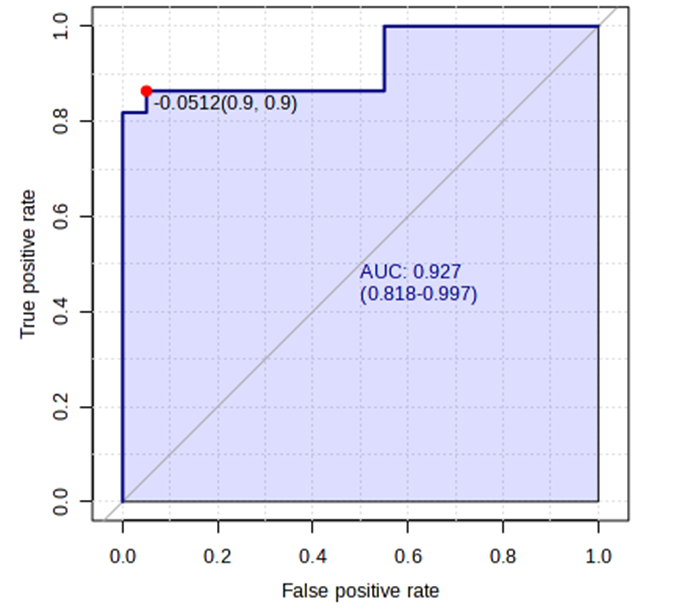 | 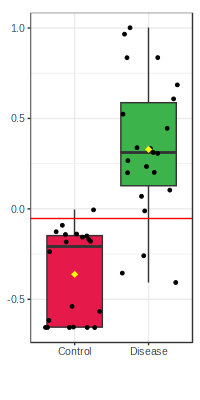 |
| --- | --- |

**5-Phenylpyruvic acid**

| 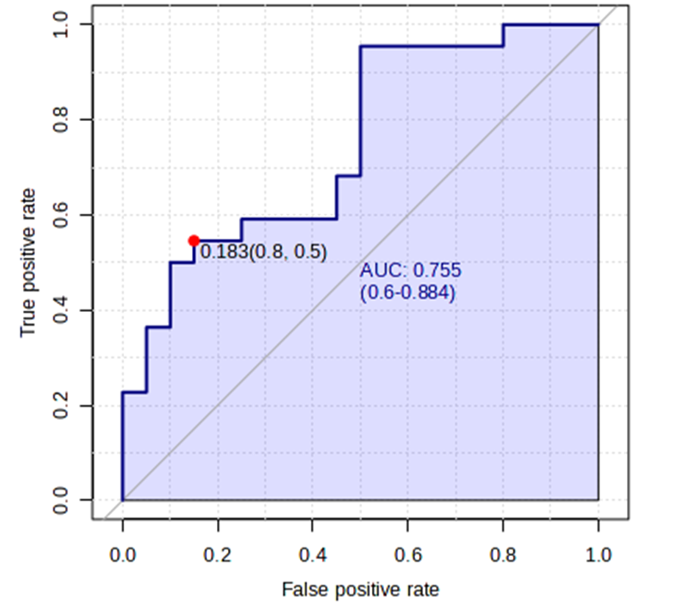 | 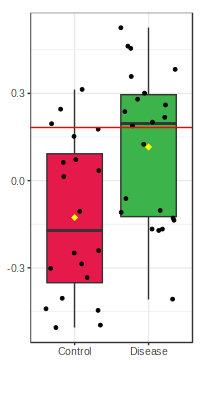 |
| --- | --- |

**Tyrosine**

| 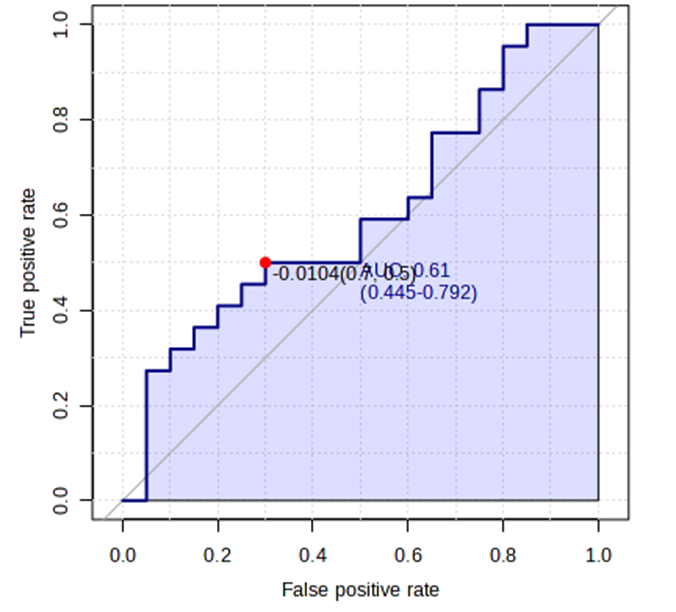 | 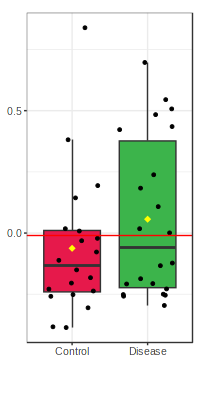 |
| --- | --- |

**Hydrocinnamic acid**

| 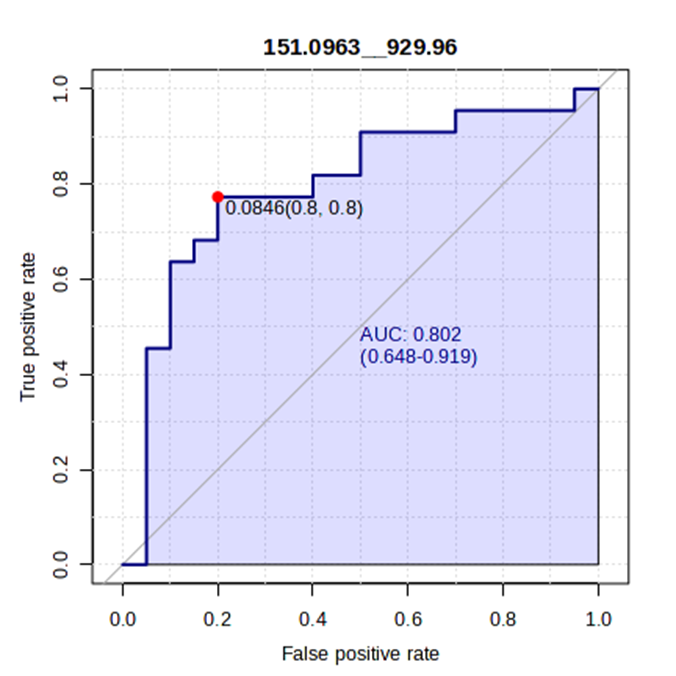 | 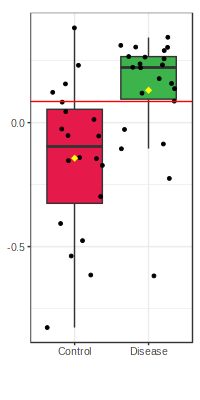 |
| --- | --- |

Cinnamic acid

| 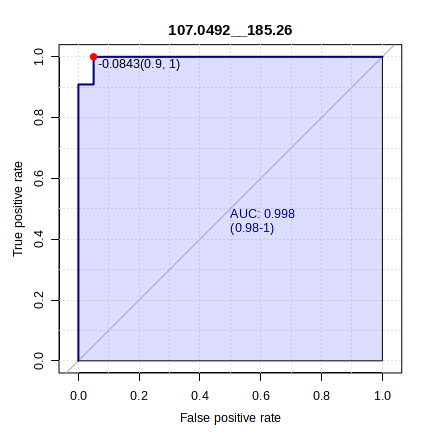 | 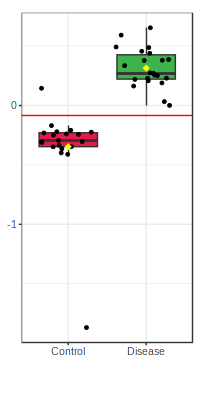 |
| --- | --- |

**Arginylleucine**

| 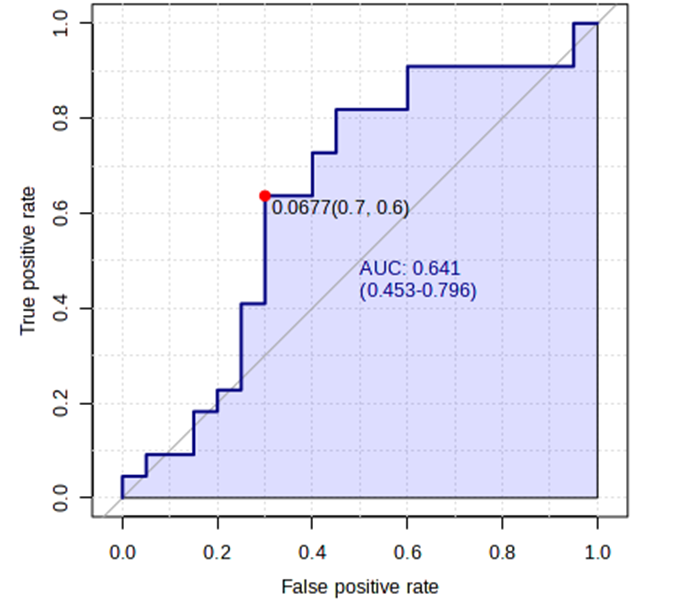 | 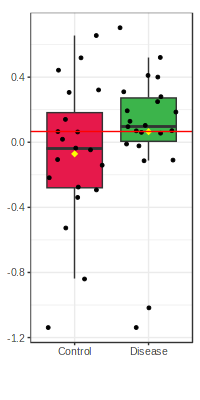 |
| --- | --- |

Indole-3-Carboxylica acid

| 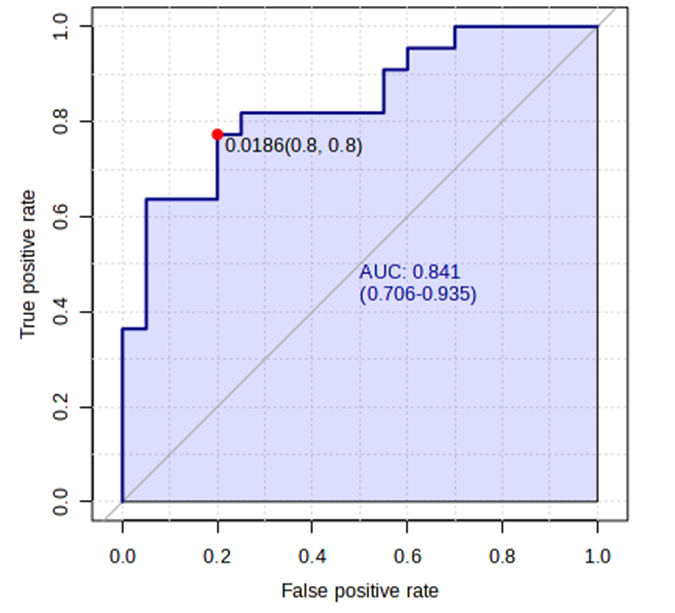 | 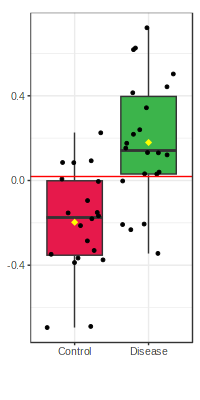 |
| --- | --- |

**2-Phenylbutyric acid**

| 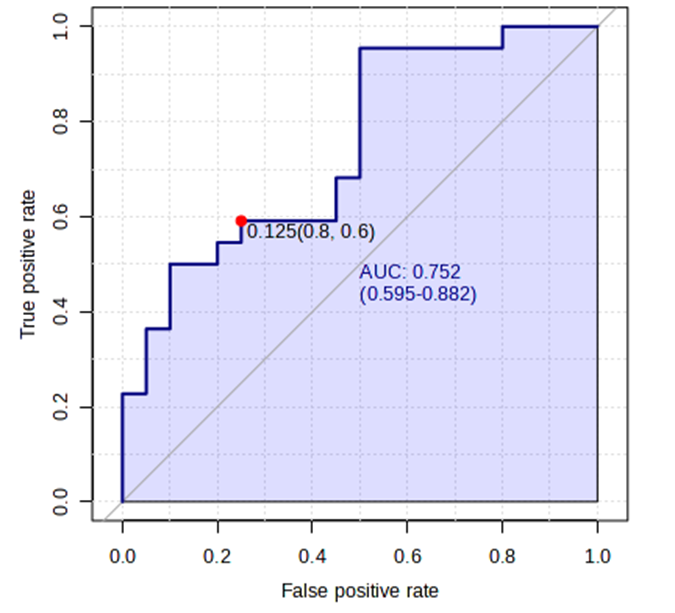 | 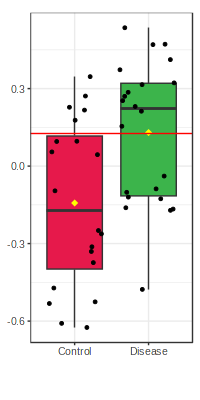 |
| --- | --- |

| 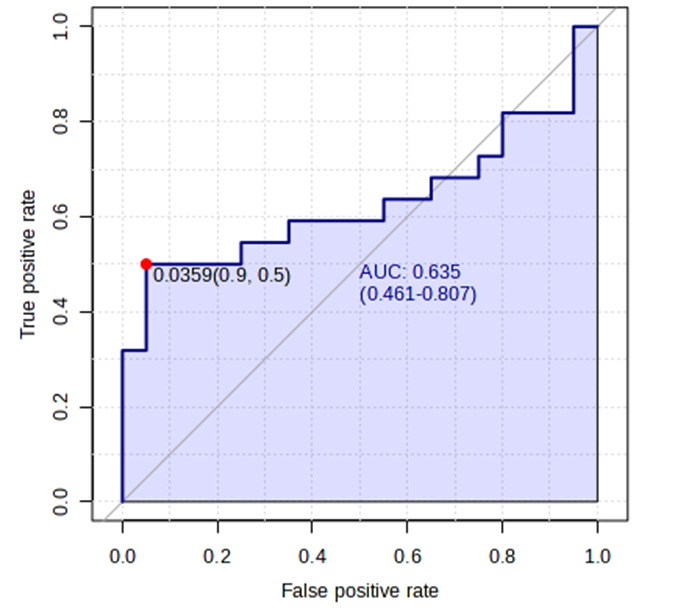 |  |
| --- | --- |

**Glutamylproline**

|  |  |
| --- | --- |

**Valine**

|  |  |
| --- | --- |

**Sphingosine 1-phosphate**

|  |  |
| --- | --- |
